# Machine Learning–Based Cognitive Assessment With The Autonomous Cognitive Examination: Randomized Controlled Trial

**DOI:** 10.2196/67446

**Published:** 2025-07-30

**Authors:** Calvin Howard, Amy Johnson, Sheena Baratono, Katharina Faust, Joseph Peedicail, Marcus Ng

**Affiliations:** 1 Center for Brain Circuit Therapeutics, Brigham & Women’s Hospital, Harvard Medical School, 60 Fenwood RoadBoston, MA, 02215, United States; 2 Department of Neurology, Brigham & Women’s Hospital, Harvard Medical School, Boston, MA, 02215, United States; 3 Klinik für Neurologie mit Experimenteller Neurologie, Charité – Universitätsmedizin Berlin, Corporate Member of Freie Universität Berlin and Humboldt-Universität zu Berlin, Berlin, 10117, Germany; 4 Clinician Investigator Program, Postgraduate Medical Education, University of Manitoba, Winnipeg, Manitoba, R3E 3P4, Canada; 5 Faculty of Science, University of Manitoba, , Winnipeg, Manitoba, R3T 2M8, Canada; 6 Department of Neurosurgery, Charité – Universitätsmedizin Berlin, Corporate Member of Freie Universität Berlin and Humboldt-Universität zu Berlin, Berlin, Germany; 7 Section of Neurology, Department of Internal Medicine, University of Manitoba, Winnipeg, Manitoba, R3A 1R9, Canada; 8 Graduate Program in Biomedical Engineering, University of Manitoba, Winnipeg, Manitoba, R3T 5V6, Canada

**Keywords:** neurology, cognitive test, neuropsychology, cognition, autonomous cognitive examination, cognitive examination, machine learning, ML, dementia, ACoE, cognitive assessment, attention, memory, language, fluency, visuospatial function

## Abstract

**Background:**

The rising prevalence of dementia necessitates a scalable solution to cognitive assessments. The Autonomous Cognitive Examination (ACoE) is a foundational cognitive test for the phenotyping of cognitive symptoms across the primary cognitive domains. However, while the ACoE has been internally validated, it has not been externally validated in a clinical population, and its ability to render accurate appraisals of cognition is unknown. Further, it is unclear if these phenotypic assessments are useful in clinical tasks such as screening patients with and those without impairments.

**Objective:**

The objective of this study is to validate the ability of the ACoE to reliably phenotype cognition and to act as a screening examination relative to standard paper-based tests.

**Methods:**

To compare the evaluations of the ACoE to established paper-based tests, 46 patients with neurological disorders were enrolled in a randomized crossover study and received either the ACoE or a standard paper-based cognitive test. Patients received either the Addenbrooke Cognitive Examination-3 (ACE-3; n=35) or the Montreal Cognitive Examination (MoCA; n=11). We evaluated 3 primary metrics of the ACoE’s performance relative to paper-based tests: (1) interrater reliability of overall cognitive scores, (2) interrater reliability of cognitive domain scores, and (3) ability to classify patients similarly to paper-based tests.

**Results:**

The ACoE’s overall cognitive assessments were significantly reliable (ICC [intraclass correlation coefficient]=0.89; *P*<.001). Each cognitive domain’s assessments were also significantly reliable, including attention (ICC=0.74; *P*_FWE_<.001), language (ICC=0.89; *P*_FWE_<.001), memory (ICC=0.91; *P*_FWE_<.001), fluency (ICC=0.74; *P*_FWE_<.001), and visuospatial function (ICC=0.78; *P*_FWE_<.001). The ACoE was also able to successfully diagnose patients similarly to both paper-based tests (area under the receiver operating characteristic curve=0.96; *P*_FWE_<.001).

**Conclusions:**

In this study, we evaluated if the ACoE could reliably phenotype cognitive symptoms relative to the assessments of established standard paper-based cognitive assessments. We found that the ACoE reliably phenotypes patient cognition, which can be used to screen patients. In the future, these cognitive phenotypes may be used to diagnose specific etiologies.

## Introduction

Current projections estimate 150 million patients living with dementia worldwide by 2050, with 57 million as of 2019 [1]. The aging population presents a considerable diagnostic challenge. This challenge has resulted in diagnostic timelines requiring 3 years or longer from symptom onset [2-6] with a large portion of patients with dementia remaining undiagnosed [2,3,7,8].

Digital cognitive assessments (DCAs) offer a potential method to address the diagnostic challenge and help improve diagnostic timelines [9-11]. Among other things, 2 factors that influence the utility of DCAs at a population level are accessibility and generalizability [11,12].

To increase accuracy, some DCAs may sacrifice a degree of accessibility to better control testing conditions [13,14]. Often this means DCAs achieve their exceptional performance by requiring specific hardware [15-21] in-office expert administrators [13,15,16,18-20,22-24] or by limiting variability by using mouse-and-keyboard questionnaires [15,25-28]. Eliminating these requirements could improve accessibility for nonaffluent, rural, or patients with cognitive impairment.

Other DCAs may sacrifice some generalizability to focus on a specific disease. These DCAs often use disease-specific examination maneuvers [15,18,22,23,29-31] or disease-specific algorithms [32-37] with both approaches meant to maximize detection of specific diseases. By taking a step backward from highly focused assessments, providing a thorough cognitive examination may improve generalization.

We previously developed Autonomous Cognitive Examination (ACoE) to improve accessibility and generalizability. The ACoE uses various machine learning algorithms to provide a thorough assessment of cognition in a naturalistic and remote assessment [38,39]. However, it has not been clinically validated, and its utility is unknown.

Here, we evaluate the validity of the ACoE. We compare the ACoE against a comprehensive test, the Addenbrooke Cognitive Examination-3 (ACE-3) [40,41] and a ubiquitously used test, the Montreal Cognitive Assessment (MoCA) [42]. First, we compare the reliability of the ACoE to phenotype overall cognition and cognitive symptoms compared to the ACE-3. We then evaluate the ability of the ACoE’s phenotypic output to achieve similar screening results as the ACE-3 and MoCA.

## Methods

### Study Design

A 2-period double crossover randomized controlled study was used. The double-crossover study design mitigates learning bias and has been previously shown to improve statistical power [43]. Patients were randomized in a 1:1 ratio to receive either the ACoE or paper-based test first, then returned 1‐6 weeks later to receive the other test. Intertest duration was limited to 1‐6 weeks to control for time-, medication-, or pathology-related cognitive changes to balance for learning bias while also minimizing disease or mental state between tests [37]. Only patients receiving the ACE-3 were randomized to enable comparison of the cognitive evaluation of the ACoE to the ACE-3 (Figure S1 in Multimedia Appendix 1). No changes were made to the study design after initiation.

Patients with and without cognitive complaints were recruited. The inclusion criterion was English fluency and being 18 years and older. English fluency was evaluated by the attending clinician. Exclusion criteria were acute medical conditions contributing to the cognitive state, acute psychiatric disorders contributing to the cognitive state, delirious states, or disabilities restricting ability to use screens, disabilities restricting the ability to receive visual and auditory instructions or developmental delay. This trial follows CONSORT (Consolidated Standards of Reporting Trials) guidelines (the CONSORT-EHEALTH [Consolidated Standards of Reporting Trials of Electronic and Mobile Health Applications and Online Telehealth] checklist is provided in Checklist 1).

### Participants

Our study cohort comprised patients from neurology clinics across the Health Sciences Centre, University of Manitoba. Participants who had previously consented to be contacted for research were contacted by study staff by phone for enrollment. Our overall cohort (n=46) is composed of patients receiving both the ACE-3 and MoCA, with each group receiving the ACoE. To understand how the ACoE performs across a range of cognition and age states, we recruited patients ranging from healthy controls to probable Alzheimer disease, and a range of ages spanning 33-82 years. Further details are available (Table 1). The patients receiving the ACE-3 were randomized into 2 arms, with further details available for each arm (Table S1 in Multimedia Appendix 1).

An additional validation cohort of older patients from the Health Sciences Center, University of Manitoba, was recruited in a nonrandomized fashion. These patients (n=20) were 65 years and older with an age range spanning 67-86 years of age, and received the MoCA as well as the ACoE in a nonrandomized fashion.

**Table 1.** Patient characteristics split by paper test received.

Characteristic	ACE-3^a^	MoCA^b^
Average age (years), median (IQR)	45.3 (46)	61.7 (65)
Age categories (years), n (%)		
25 years and younger	0 (0)	0 (0)
25‐45	19 (54)	2 (18)
45‐65	12 (34)	4 (36)
65 years and older	4 (11)	5 (46)
Sex, n (%)		
Male	17 (48)	5 (46)
Female	18 (52)	6 (44)
Ethnicity, n (%)		
White	19 (54)	7 (64)
Indigenous	5 (14)	3 (27)
Indian	4 (11)	1 (9)
Filipino	3 (9)	0 (0)
African	1 (3)	0 (0)
Eastern European	3 (9)	0 (0)
Education, n (%)		
Less than secondary	2 (6)	4 (36)
Secondary	27 (77)	6 (66)
Postsecondary	5 (14)	1 (8)
Graduate or professional	1 (3)	0 (0)
Employment status, n (%)		
Unemployed	11 (31)	3 (27)
Employed	24 (69)	8 (73)
Diagnosis, n (%)		
Neurologically healthy	11 (31)	5 (46)
Mild cognitive impairment	7 (20)	2 (18)
Probable Alzheimer disease	3 (9)	4 (36)
Epilepsy	14 (40)	0 (0)
Total, n (%)	35 (76)	11 (24)

a ACE-3: Addenbrooke’s Cognitive Examination-3.

b MoCA: Montreal Cognitive Assessment.

### Patient Recruitment

Patients indicating interest in clinical research were contacted by study team members via phone. Interested patients were screened for inclusion and exclusion criteria and enrolled. This study was not blinded. At the first clinic visit, patients were again screened for inclusion or exclusion criteria by a physician.

### Study Sample Size

Power analysis for this study was based on two separate analyses: (1) sample required to have powered assessment reliability of cognitive phenotyping, and (2) sample required to have powered assessment of screening performance.

To assess the reliability of phenotyping, the intraclass correlation coefficient (ICC) is the primary metric used in the validation of tools [44], including cognitive examinations [21,45,46]. Previous statistical analysis has been performed to evaluate the amount of participants required to achieve powered analysis using the ICC in human research [47]. The power analysis published in this study demonstrates that 35 participants are required to achieve 80% statistical power. The 3 variables dictating the power analysis were number of observations (k), minimum ICC (ρ), and confidence interval precision (ω). Values were chosen according to our study design. Patients received 2 separate observations (ACoE observation and ACE-3 observation), setting k to 2. We chose a minimum acceptable ICC of 0.80, setting ρ to 0.80. Finally, we chose maximum potential confidence interval half-widths of 0.15, setting ω to 0.15.

To assess the screening performance, the area under the receiver operating characteristic curve (AUROC) is the measurement of choice. To assess the sample size required for a powered analysis of the AUROC, we used the Hanley and MacNeil [47] formula. We targeted 80% power. The required sample size for the AUROC varies with test performance. If a test has an AUROC of 0.70, 16 positive and negative cases are required, which decreases to 8 at an AUROC of 0.80, and 2 at an AUROC of 0.90. We recruited a total of 16 positive and negative cases to account for the range of potential ACoE AUROC values.

### Test Administration

Patients were tested in a quiet environment by a physician trained in cognitive examination. Caregivers were allowed to join but could not participate in the examination. A trained physician examiner directly administered the examination, evaluated responses, and summated scores.

Paper-based tests were administered via standard paper forms and evaluated using their established scoring guideline. The ACE-3 is composed of 19 questions spanning the domains of language, executive function, memory, and visuospatial function [40]. Language is separated into “language” and “fluency” on the ACE-3 for relevance to Parkinson disease. The ACE-3 total score ranges from 0‐100, with 100 representing a maximum function. The MoCA is another ubiquitous test which briefly evaluates language, executive function, memory, and visuospatial function with 13 questions [42]. The total score of the MoCA ranges from 0‐30, with 30 representing maximum function.

The ACoE was administered by a touchscreen and microphone-equipped tablet. The ACoE administered itself to the patient without interference or prompting from the examiner. ACoE responses were automatically scored and summated.

### ACoE Evaluation of Cognition

**Figure 1.**
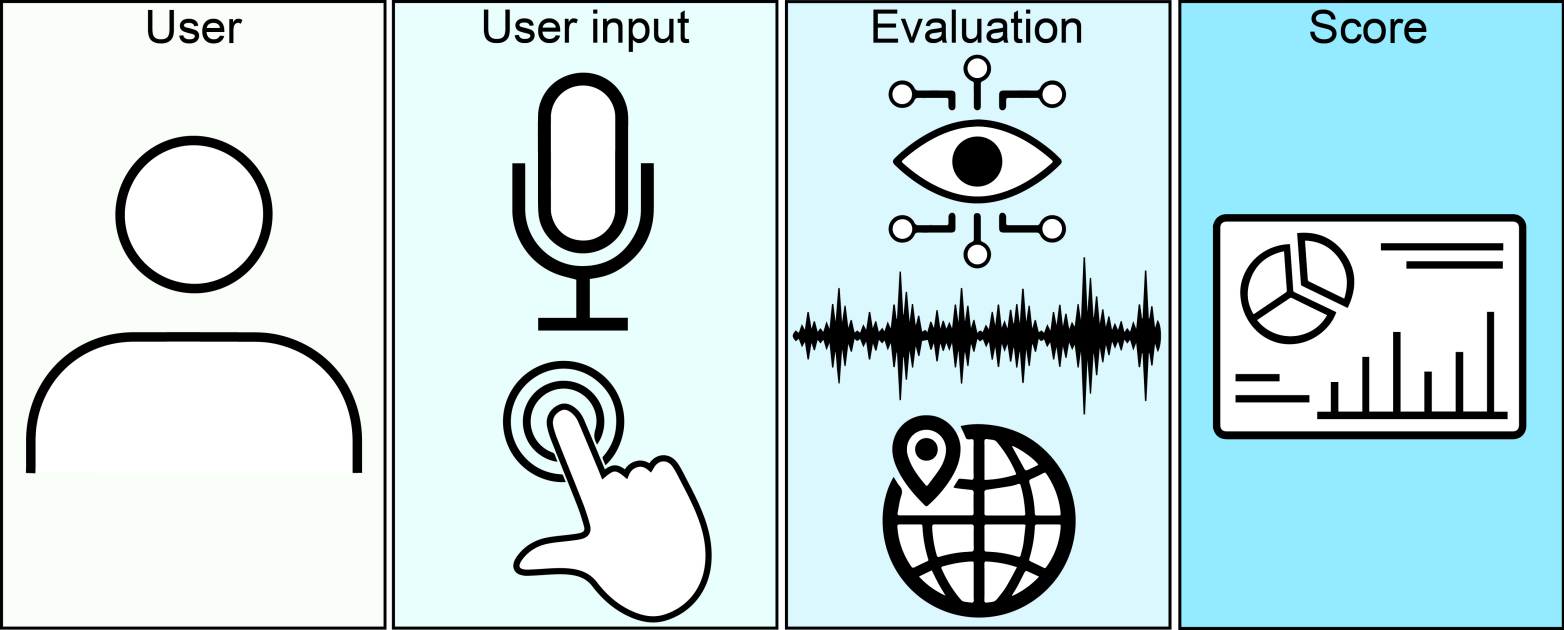
The components of the ACoE (Autonomous Cognitive Examination). The examination begins with an individual user, either alone or accompanied by a caregiver. User inputs are primarily via microphone and touchscreen, although mouse and keyboard are allowed. User inputs are evaluated using a host of 79 algorithms. These algorithms span 3 primary domains: computer vision for the evaluation of drawings, natural language processing for the evaluation of speech, and expert algorithms to evaluate all other inputs, such as geolocating a patient to verify orientation to space and time. Scores output by the individual evaluation algorithms are summated, providing the final total score as well as the scores for cognitive domains of memory, language, fluency, visuospatial, and attention.

The ACoE receives user input via any hardware device with an internet connection, microphone, and touchscreen. The ACoE questions are answered via microphone and touchscreen, although a keyboard and mouse can be used. The user proceeds through an examination from end to end, which automatically administers voiced instructions with closed captioning (Figure 1).

The ACoE consists of 19 questions within the primary domains of memory, language, fluency, visuospatial, and executive function (also referred to as attention). Attention comprises executive function and actual attention. This classification system of the cognitive functions is modeled after the ACE-3 [40]. A table of each question and associated primary cognitive domain is available (Table S2 in Multimedia Appendix 1).

For each question, the patient is given unrestricted time to answer. The patient receives specific instructions both visually and by audio at the start of each question. The instructions were allowed to be repeated up to 3 times, but no further assistance was provided. 13 questions were answered with speech, 3 with touchscreen inputs of drawing or manipulating on-screen objects, 2 with drop-down menus, and 1 with typing.

At the end of the examination, total score and cognitive domain scores are calculated. There are 5 memory questions totaling 26 points, 1 fluency question totaling 14 points, 7 language questions totaling 40 points, 3 executive questions totaling 18 points, and 3 visuospatial questions totaling 16 points (Table 2). The total score is 100 points.

**Table 2.** Breakdown of Autonomous Cognitive Examination scoring.

Cognitive domain	Number of questions	Total score
Overall	19	100
Memory	4	26
Language	8	40
Fluency (language subdomain)	1	14
Executive function	3	18
Visuospatial function	3	16

### ACoE Algorithms to Evaluate Patient Input

A unique algorithm was developed for each of the 19 primary questions, including subquestions (Table S3-S21 in Multimedia Appendix 1). This resulted in 76 unique algorithms, which have been previously described (Figure 1) [38,39,48,49]. In brief, each algorithm corresponds to how one question on the ACE-3 is scored and attempts to estimate the scoring of the ACE-3 for the corresponding question. These span 3 primary algorithmic domains: computer vision, natural language processing, and expert algorithms.

The 3 computer vision algorithms enable the testing of visuospatial drawing tasks. For example, the overlapping infinity copy, cube copy, and clock drawing test evaluated by SketchNet, a custom convolutional neural network created for the ACoE [49]. These are involved in visuospatial function evaluation. The computer vision algorithms are responsible for assessing 8/100 ACoE points.

The 48 natural language processing algorithms evaluate spoken answers, speech quality, sentence structure, and word pronunciation. The tasks these are responsible for are immediate recall, mental arithmetic, delayed word recall, phonemic list generation, semantic list generation, semantic memory, sentence writing, word repetition, sentence repetition, naming, reading aloud, counting, identifying, and partially obscured objects for simultanagnosia. These are involved in the evaluation of memory, language, executive, and visuospatial tasks. The natural language processing algorithms are responsible for 68/100 ACoE points.

The 25 expert algorithms allow the evaluation of complex tasks which do not easily fit into common machine learning approaches. Examples include an algorithm to evaluate a patient’s orientation to their location in space by combining natural language processing with geolocation. Similar algorithms are used to assess orientation to time, ability to follow on-screen commands, recognize objects, or recall with prompting. These are involved in the evaluation of memory, language, executive, and visuospatial tasks. The questions requiring expert algorithms compose 24/100 ACoE points.

### ACoE Deployment

**Figure 2.**
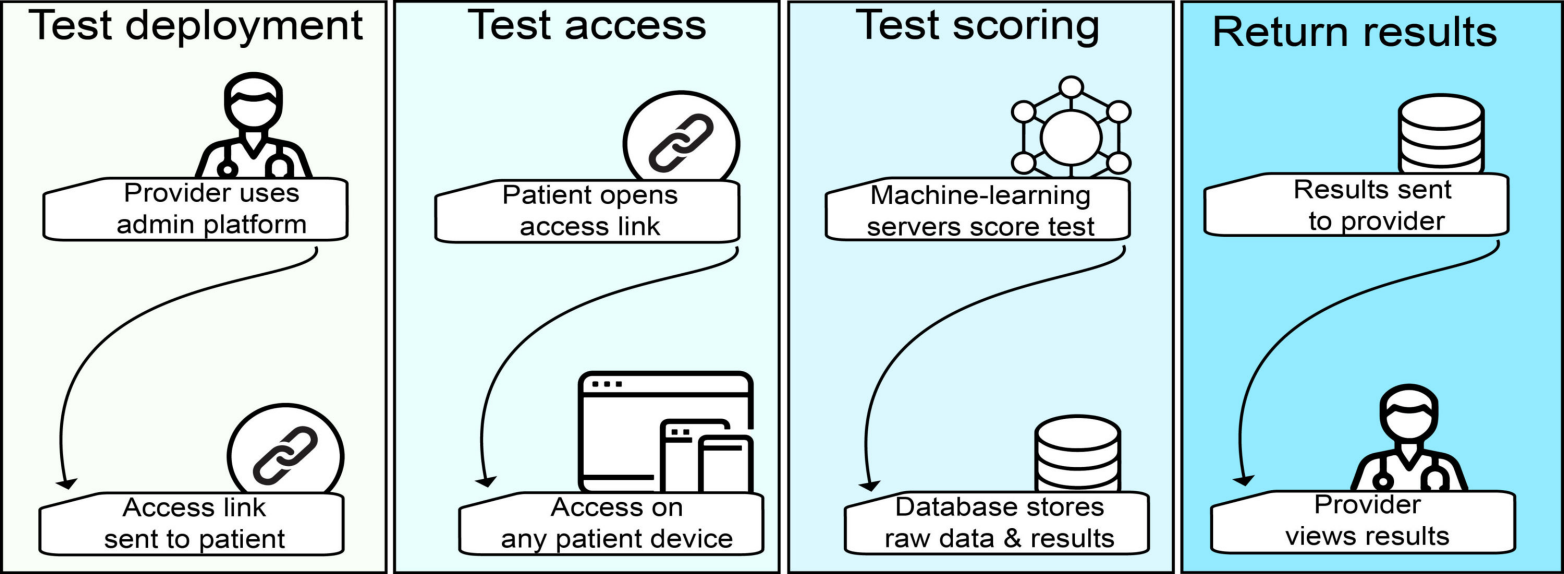
Use of the Autonomous Cognitive Examination (ACoE). Test Deployment: the testing process is initiated by the health care provider. The administrative platform is used to generate an access link for a patient and is sent to the patient. Patients can also access ACoE links directly for self-assessment. Test Access: patients click the access link to access the examination, which runs on all devices. Test Scoring: raw patient responses are sent to the scoring server. The scoring server receives the patient files, preprocesses them, and administers the evaluation algorithms to them. Summated patient scores are then stored in a database. Return Results: summary results, broken down by cognitive domain and overall performance, are sent to the administrative platform so the health care provider can view the patient’s results.

The ACoE is hosted on Amazon Web Services to provide accessibility to roughly 75% of the globe. The ACoE leverages a cloud-based format to enhance accessibility for patients, allow physicians to test patients regardless of their location, and provide secure storage of data. Patients access the ACoE through links that are sent to them by an ACoE administrator. Each link is specific to the given patient and becomes inactive after use. Each link is validated upon use, and invalid links are rejected access. The patient then receives the ACoE, and results are sent via encryption to the scoring server, which is stored on a private subnet. The scoring server then returns patient scores in an encrypted manner to the administrative user’s database. This allows the clinician using the ACoE’s administrative platform to view patient results as they are completed. The raw files for each patient are stored in an anonymized format on an encrypted and private database. The ACoE and administrator platform are Health Information Privacy Protection Act compliant. A diagrammatic representation of the process is provided (Figure 2).

### Evaluation of Cognitive Phenotyping Reliability

To evaluate the ability of the ACoE to reliably phenotype overall cognition and cognitive subdomains, the ICC was used to compare the similarity of scores between each patient’s ACoE and ACE-3 scores. To evaluate systematic deviances in group-level scores, we compared the central tendency of ACoE versus ACE-3 score with Wilcoxon signed-rank testing, a paired evaluator of the median.

### Evaluation of Diagnostic Reliability

A receiver operating characteristic was constructed comparing the ACoE classifications to ACE-3 (n=35) and MoCA (n=11) classifications. AUROC was calculated as a metric of reliability in diagnosis. Youden J was calculated to derive the optimal classification threshold, enabling a direct comparison of thresholds between ACoE and ACE-3 [50]. To assess the confidence with which a classification of impairment can be made, bootstrapped sensitivity and specificity were calculated at all ACoE scores. Specifically, patients were resampled with replacement 10,000 times, which is a reliable method of generating CIs [51,52]. Labels of cognitively impaired versus intact were made by an expert clinician in conjunction with the ACE-3 and MoCA established cutoffs, 26/30 on the MoCA and 83/100 on the ACE-3 [40,42,53].

### Statistics

All analyses were performed in Python. Central tendency, correlation, normality, and general linear model analyses were performed with Statsmodels. Power analyses were performed in accordance with established techniques, using a nomogram for ICC and a Python implementation [47]. ICC was calculated with the Pingouin package [54].

Spearman correlation was used for ordinal data. Paired Wilcoxon tests were used for ordinal data and when normality was violated as measured by the Shapiro-Wilk test. Multiple comparisons were corrected with Bonferroni correction.

A total of 2 methods of intraclass correlation were used. The 2-way random effects model is commonly used to evaluate agreement between clinical evaluations and scales and is what we use to derive our primary ICC results [44]. We focus on evaluating the consistency of the 2 raters, the ACoE and the ACE-3. To perform sensitivity analyses, we use the more conservative one-way random effects model [44].

A multivariate regression (ordinary least squares) was used to relate independent variables age, cognitive status, ethnicity, sex, educational status, and randomization group to the dependent variable: ACoE score. These variables were selected to specifically evaluate the effect of known demographic variables on cognitive test scores.

An adjustment factor was developed to account for the effect of patient age on their ACoE score derived from the multivariate regression relating age to ACoE score (Equation S1 in Multimedia Appendix 1). The coefficient of age from this formula can be used to adjust ACoE scores for age (Equation S2 in Multimedia Appendix 1).

### Ethical Considerations

The study has been conducted in accordance with the ethical standards. This study was conducted in accordance with ethical standards as described in the 1964 Declaration of Helsinki and its subsequent amendments. Approval was achieved by the Research Ethics Board of the Health Sciences Center, the University of Manitoba (#HS25666) for human participants research. Informed consent was achieved for the assessment of each patient and further analysis of results. Patients or their caregivers provided written consent at the first clinic visit. All signed institutional review board–approved consent forms. Substitute decision makers were included in the consent process of patients with cognitive impairment. All data were anonymized and deidentified. Patients were not compensated for involvement in this study.

## Results

### Recruitment Results

**Figure 3.**
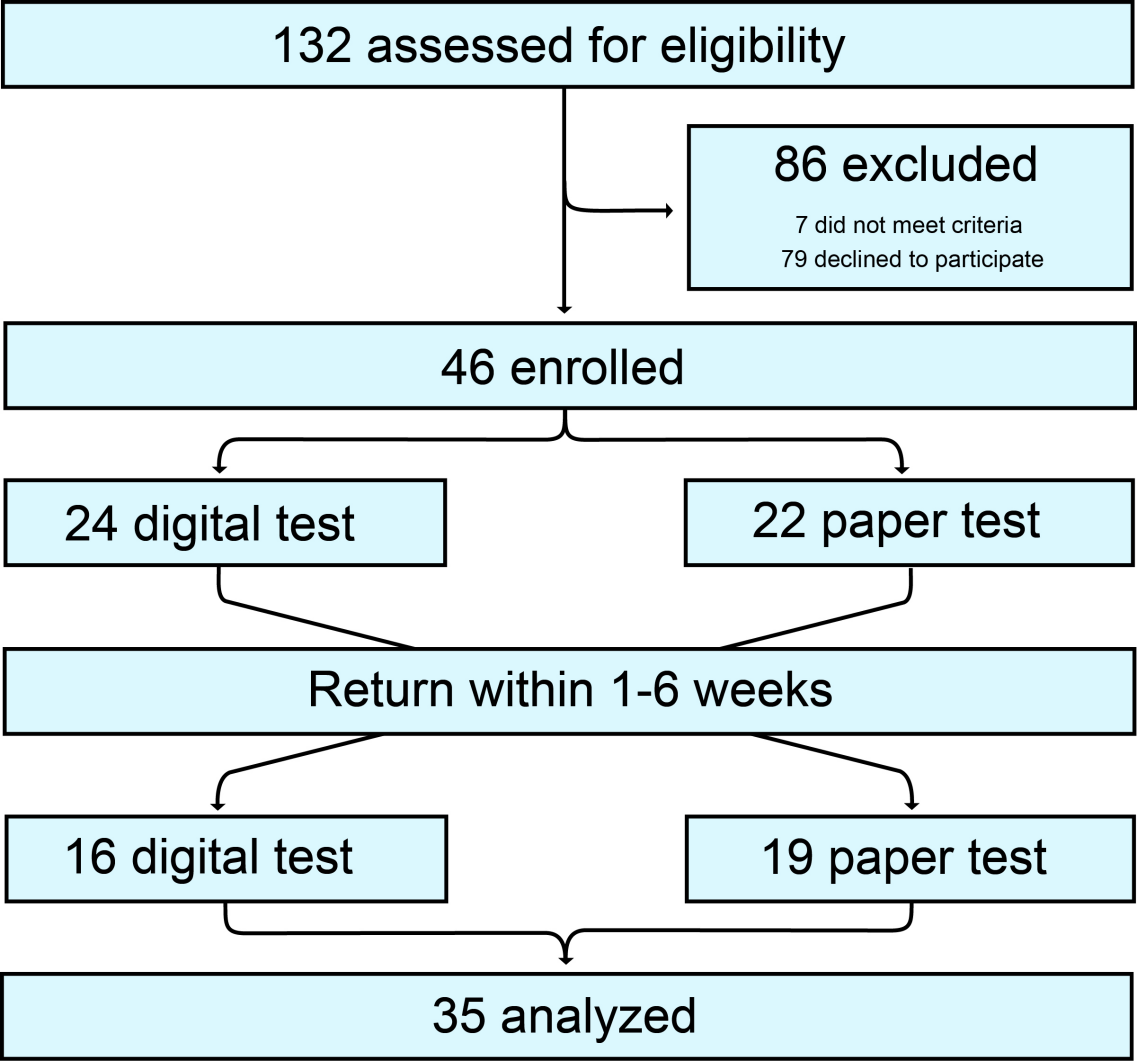
CONSORT (Consolidated Standards of Reporting Trials) flow diagram of crossover trials. In total, 132 patients were evaluated for enrollment. Then, 86 patients were excluded, 46 patients were enrolled in the trial, with 24 randomized to receive the Autonomous Cognitive Examination first and 22 were randomized to receive the Addenbrooke Cognitive Examination-3 first. Subsequently, each arm then crossed over and received the other test. In total, 11 patients were lost to follow-up, and 35 patients completed the study and were assessed.

A total of 132 patients were assessed for eligibility. In total, 79 patients denied enrollment, and 7 did not meet the inclusion criteria. Around 46 patients were randomized, with 24 patients randomized to receive the ACoE first (Group 1) and 22 to receive the ACE-3 first (Group 2). Around 11 patients were lost to follow-up. A total of 35 patients completed the study and were analyzed (Figure 3). There were no adverse outcomes reported. The characteristics of each arm are available (Table S1 in Multimedia Appendix 1).

We next evaluated for appropriate randomization. Cognitive scores were not significantly different between the 2 arms (Wilcoxon test, *P*=.59; Multimedia Appendix 1). Nor were there significant differences in the number of patients in each arm (chi-square, *P*=.46).

### Reliability of the ACoE

**Figure 4.**
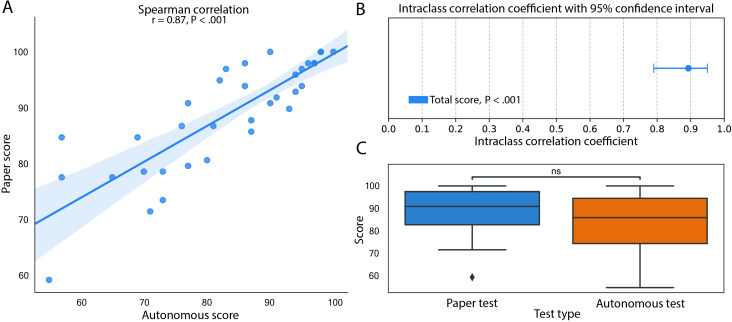
The ACoE reliably assesses overall cognition. (**A**) Spearman correlation of ACoE to ACE-3 demonstrates high correlation of patient scores between the tests (Rho=0.87, *P*<.001). (**B**) The ACoE reliably rates patients similarly to the ACE-3 (ICC=0.91, *P*<.001). 95% CI of the intraclass correlation coefficient is presented. (**C**) Wilcoxon test of ACoE relative to the ACE-3 demonstrates there is no significant difference between the median scores. Scores are presented as percent.

We first evaluated the overall correlation of the ACoE’s assessment of cognition to the ACE-3’s assessment (Figure 4A). There was a significant positive correlation between the 2 tests (Spearman Rho=0.87; *P*<.001). To more conservatively assess how well each test administered scores on a patient-by-patient basis, we investigated the interrater reliability (Figure 4B). We found the ACoE and ACE-3 had significant interrater reliability (ICC=0.89, 95% CI 0.79‐0.95; *P*_FWE_<.001). We tested the reliability of this result using the most conservative ICC analysis, and found the agreement was still significant (*P*_FWE_<.001).

To assess for significant bias in overall scoring, we compared the median score of the ACoE and ACE-3 (Figure 4C). Distributions were found to be nonnormal (Shapiro-Wilk, *P*=.01), and nonparametric testing found no significant difference between test scores (Wilcoxon Test, *P*=.05). Reliability tests also revealed no difference between the means when tested parametrically (*t*_45_ test, *P*=.89).

### Reliability of Cognitive Domain Assessment

**Figure 5.**
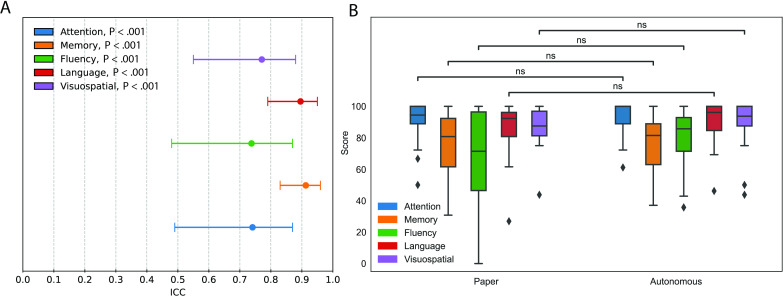
The Autonomous Cognitive Examination (ACoE) reliably assesses cognitive domains. (**A**) Reliability of ACoE cognitive domain evaluations ranges from high to very high as measured by ICC. 95% CIs are presented with each ICC. (**B**) The Wilcoxon test demonstrates no significant difference between central tendencies of cognitive domains between either test. Scores are presented as percent.

Next, we investigated the reliability of each cognitive domain’s assessment (Figure 5A). The ACoE was reliable for all cognitive domains, including attention (ICC=0.74; *P*<.001), language (ICC=0.89; *P*_FWE_<.001), memory (ICC=0.91; *P*_FWE_<.001), fluency (ICC=0.74; *P*_FWE_<.001), and visuospatial function (ICC=0.78; *P*_FWE_<.001). To test the robustness of these results, we used the most conservative form of the ICC and assessed each domain again. All results remained significant (*P*_FWE_<.001).

We next compared the distribution of cognitive domain scores between the 2 tests (Figure 5B). Neither ACoE nor ACE-3 cognitive domain scores were normally distributed (Shapiro-Wilk, *P*=.02). Nonparametric testing of the medians revealed no significant differences between ACoE and ACE-3 in all domains, including attention (Wilcoxon Test, *P*_FWE_=.71), memory (Wilcoxon Test, *P*_FWE_=.37), visuospatial function (Wilcoxon Test, *P*_FWE_=.59), fluency (Wilcoxon Test, *P*_FWE_=.34), and language (Wilcoxon Test, *P*_FWE_=.60). Sensitivity testing with parametric evaluation again revealed no differences in attention (*t_45_* test, *P*=.32), memory (*t_45_* test, *P*_FWE_=.48), visuospatial function (*t_45_* test, *P*_FWE_=.62), fluency (*t_45_* test, *P*_FWE_=.51), and language (*t_45_* test, *P*_FWE_=.76). Means, SEs, and medians are available in the supplements (Table S22 in Multimedia Appendix 1).

### Reliability of Underlying Algorithms

We next evaluated if the algorithms used to enable naturalistic completion of the exam were reliable (Figure S3 in Multimedia Appendix 1). We found the 3 primary algorithms were reliable, including computer vision (ICC=0.67, *P*_FWE_<.001), natural language processing (ICC=0.86, *P*_FWE_<.001), and the expert algorithms (ICC=0.82; *P*_FWE_<.001). Results were stable after repetition with the more conservative ICC, including computer vision (*P*_FWE_=.015), natural language processing (*P*_FWE_<.001), and expert algorithms (*P*_FWE_<.001).

Nonparametric testing revealed no biases in overall scoring of the algorithms, including computer vision (Wilcoxon test*; P*_FWE_=.26), natural language processing (Wilcoxon test; *P*_FWE_=.44), and the expert algorithms (Wilcoxon test; *P*=.68). Sensitivity testing with parametric evaluation also demonstrated no difference in natural language processing (*t*_45_ test; *P*=.45), computer vision (*t*_45_ test; *P*_FWE_=.39), nor the expert algorithms (*t*_45_ test; *P*_FWE_=.84) and their paper-based counterparts (Table S23 in Multimedia Appendix 1). Each of the underlying 19 questions was also assessed individually, and no significant differences in ACoE nor ACE-3 score were detected (Table S24 in Multimedia Appendix 1).

### Identifying and Adjusting Covariates Influencing ACoE Scores

To assess accessibility, we evaluated if any patient demographic factors were associated with worse ACoE performance. Controlling for cognitive status, we performed a series of multivariate regressions relating each demographic variable to ACoE scores (Figure S4 in Multimedia Appendix 1). Only age was significantly negatively related to ACoE score (β_age_=−0.22; *P*_FWE_=.004). Interestingly, age did not interact with cognitive status to compound the effect of either upon ACoE score (β_interaction_=−0.08; *P*_FWE_=.21). This was specific to the ACoE, as age was not significantly related to ACE-3 score, nor was any other demographic variable (Figure S5 in Multimedia Appendix 1).

### Statistically Removing Age Bias

Given age was associated with worse performance, regardless of cognitive status, the ACoE demonstrated a slight bias against older individuals (Figure S6A in Multimedia Appendix 1). We next evaluated if it is possible to remove this bias from patient scores post hoc by accounting for the effect of age. After regressing cognitive status out of ACoE scores (Figure S6B in Multimedia Appendix 1), age alone was significantly negatively correlated with ACoE scores (*r*=−0.37; *P*<.001). Then, we adjusted each patient’s score for their age and repeated the analysis (Figure S6C in Multimedia Appendix 1). The adjusted scores removed the bias of age (*r*=0.001; *P*=.95).

### ACoE Classifies Patients Similarly to Paper-Based Tests

**Figure 6.**
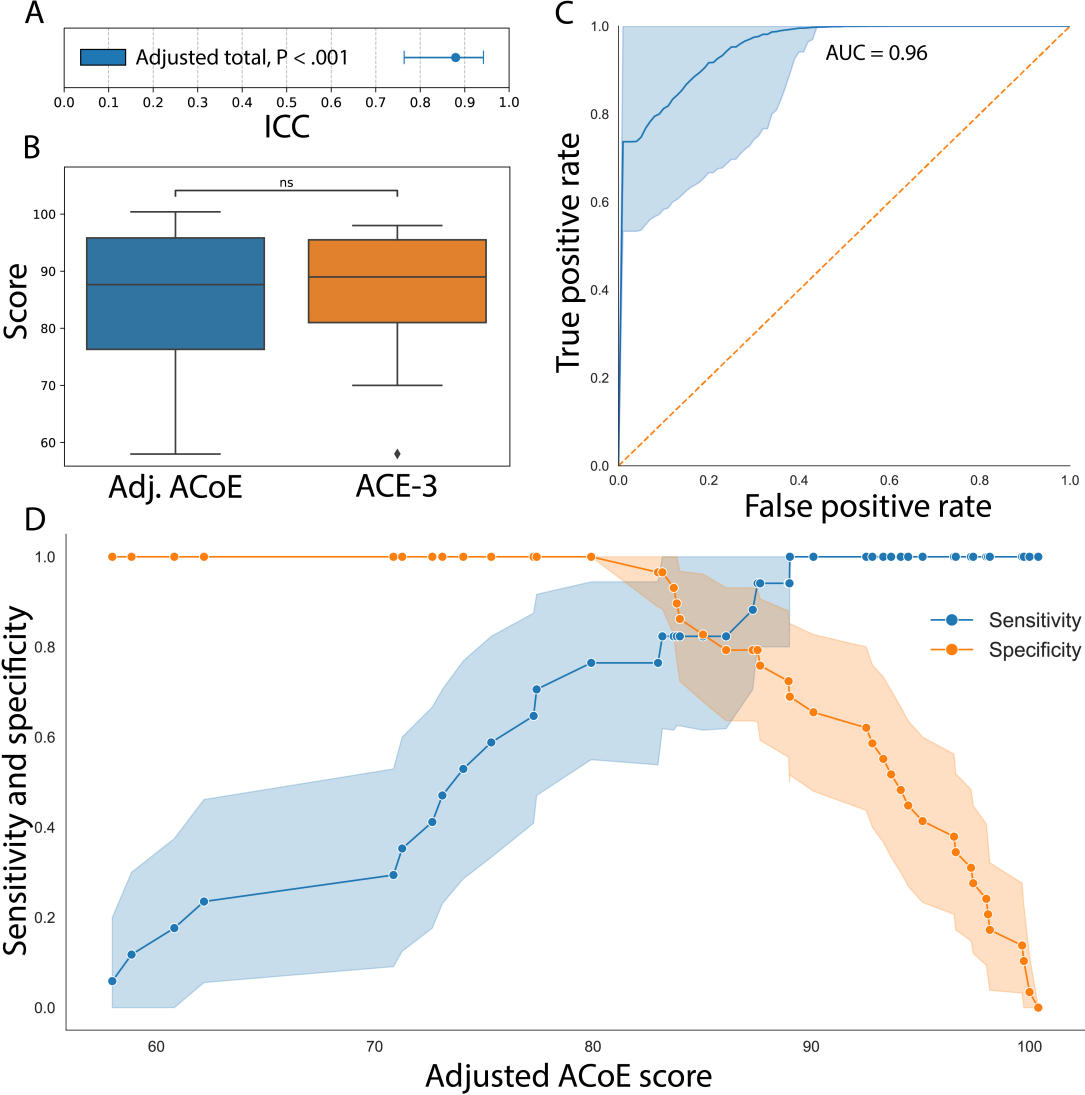
The age-adjusted Autonomous Cognitive Examination (ACoE) is reliable. (A) Reliability of age-adjusted ACoE Scores remains high. The intraclass correlation coefficient (ICC) of the age-adjusted ACoE score to patients with the ACE-3 (Addenbrooke Cognitive Examination-3) score (n=35) is 0.88 (95% CI 0.77-0.95). (B) There is no systematic bias between age-adjusted ACoE scores and ACE-3 scores. Test of medians between age-adjusted ACoE score and ACE-3 revealed no difference (*P*=.78). (C) The ACoE maintains diagnostic consistency with paper-based tests (ACE-3 and MoCA). The area under the receiver operating characteristic curve (AUC) of the age-adjusted ACoE is 0.96 compared to MoCA and ACE-3. (D) Score-specific sensitivity and specificity of the ACoE. Below a score of 83%, the ACoE achieves a specificity of 1.0 (95% CI 1.0-1.0). Above a score of 89%, the ACoE achieves a sensitivity of 1.0 (95% CI 1.0-1.0).

Next, we assess the reliability of the age-adjusted ACoE. First, we replicate the evaluation of overall cognitive score reliability (Figure 6A). The reliability between the adjusted ACoE and ACE-3 was significant (ICC=0.88, *P*_FWE_<.001). This was robust to the more conservative evaluation of ICC (*P*_FWE_<.001). The reliability within each cognitive domain remained significant, regardless of which ICC was used (*P*_FWE max_<.001).

We also repeated the evaluation of the distributions of the 2 tests (Figure 6B). Nonparametric testing revealed no significant differences (Wilcoxon test; *P*=.36). Sensitivity evaluation with parametric tests also revealed no significant difference (*t*_45_ test; *P*=.61). There were no significant differences between cognitive domain distributions, regardless of parametric or nonparametric testing (*P*_min_=.31).

Finally, we compare the overall classification adjudicated by the ACoE (Figure 5C), compared with the ACE-3 and an additional validation cohort of patients who took the MoCA (n=11). First, an ROC was constructed to compare ACoE classification to ACE-3 and MoCA classifications, demonstrating an AUC of 0.96 (*P*<.001). Youden J revealed the binary classification optimal threshold was 83%, the same diagnostic cutoff as the ACE-3 [40]. The AUCs were exceptional for both the ACE-3 alone (AUC=0.98; *P*_FWE_<.001) or the MoCA alone (AUC=0.91; *P*_FWE_<.001).

To better understand how ACoE classification accuracy varies across possible scores, we constructed sensitivity-specificity curves (Figure 6D). We find under an age-adjusted ACoE score of 83%, the test achieves high specificity (specificity=1.0, 95% CI 1.0‐1.0; *P*<.001). Above an age-adjusted score of 89%, the test achieves a high sensitivity (specificity=1.0, 95% CI 1.0‐1.0; *P*<.001). This was repeated for positive and negative predictive values, which again confirmed 83% was the threshold for detecting impairment (Figure S7 in Multimedia Appendix 1). Control analysis using the unadjusted ACoE score revealed similarly significant diagnostic ability and sensitivity or specificity (Figure S8 in Multimedia Appendix 1).

Finally, to evaluate the robustness of the ACoE in an older patient population, we investigated the performance of the age-adjusted ACoE in 20 patients of 65 (mean 70, SD 3.2) years and older. The MoCA scores and ACoE scores from these patients were related (Figure S9 in Multimedia Appendix 1), demonstrating a high correlation between their age-adjusted ACoE scores and MoCA scores (Rho=0.93; *P*<.001).

## Discussion

### Validation of the ACoE

In this study, we find the ACoE can reliably phenotype overall cognition like the ACE-3 [55], the cognitive subdomains like the ACE-3, as well as the overall screening classifications of both the ACE-3 and MoCA. This suggests the ACoE may evaluate overall cognition and come to similar conclusions as manual administration of the ACE-3 and MoCA [53]. This supports the external validity of the ACoE, but its utility in specific diseases remains to be studied.

### Accessibility of the ACoE

In developing the ACoE, we aimed to improve accessibility. However, this introduced adaptations requiring it to be automated, remotely administered, microphone-based, and touchscreen-based. These changes introduce considerable differences compared with paper-based examinations, where examiners control administration and scoring. Indeed, we find that our increased use of technology has resulted in a slight bias against older patients. Given this was unrelated to cognition, we suspect it is due to the effect of decreasing technological skills with age [56].

While we could not achieve perfect accessibility for older adults, we did develop a post hoc statistical adjustment for age. While we find no further bias among demographic variables after this adjustment, it is unlikely the test is perfectly accessible given well-known influences of demographics and even paper-based cognitive testing [57-60]. Further areas for improvement will become clear with more testing in a larger sample size on more patients with cognitive impairment.

### Application of the ACoE

We developed the ACoE to provide a general assessment of cognition, in keeping with standard tests used in current clinical practice. The ACoE performs standard cognitive exam maneuvers and applies a standard evaluation using machine learning. The purpose is to provide a clinical tool which can support physicians in getting familiar and interpretable cognitive examinations in a more scalable manner, supporting their workflows [61].

However, the ACoE does have the capacity to automate diagnoses. With the standard cognitive information we have in this study, we can identify objectively patients with cognitive impairment. The ACoE generates 3 potential classifications: confidently impaired (<83%), indeterminate (84‐88%), and confidently unimpaired (>83%). Further studies will inform how useful these are in aiding patient triaging, fast-tracking patients with cognitive impairment, or offloading patients with cognitive unimpairment.

### Future Applications

The ACoE has a wealth of data across a range of cognitive examination maneuvers and cognitive domains. While we have developed algorithms to provide a standard appraisal of this information, the future of the ACoE will be developing specialized algorithms to diagnose specific diseases. For example, our speech recognition software allows us to track not only what words a patient says, but how they say them, with implications for the aphasias and apraxias of speech [33,62]. Combination with subject-level neuroimaging will also enable the relation of disease location to cognitive symptoms, which we suspect may improve diagnostic yield [63,64].

The ACoE is a foundational model for cognitive phenotyping. While we have demonstrated here that these cognitive phenotypes can be used to screen patients, future work will need to develop specific algorithms to assist etiological diagnosis.

### Limitations

The ACoE and this study have several important limitations. First, while this study was designed to appropriate sample size by power analysis, there is a wide range of dementing etiologies and patient demographic strata. The ACoE will require further evaluation in specific etiologies, large cohorts of specific age groups, and large cohorts of patients with broad demographics.

Second, the utility of the ACoE in specific etiologies is not known. The study at hand demonstrates that the ACoE is able to reliably assess cognition across a range of diseases and cognitive status. However, in states of severe impairment such as late-stage neurodegenerative diseases, the ACoE may not be reliable. It is important to emphasize that further studies are necessary to demonstrate utility in specific diseases.

Finally, there are several other technological limitations of the ACoE. The microphone and touchscreen capabilities require patients to be able to speak with relative fluency and have reasonable manual dexterity. In conditions such as stroke, the ACoE may not be applicable. Further, the ACoE is currently only available in English, which limits cross-cultural applications. We intend to address these limitations in the future with subsequent versions of the ACoE.

### Conclusion

Here we present the ACoE, a foundational model for cognitive phenotyping. The ACoE screens patients similarly to established paper-based cognitive examinations and can discriminate between patients with cognitive impairments and those without. The scalable, remote, and automated nature of the ACoE has been developed to aid in large-scale screening and potential use in public health. In the future, additional algorithms will be developed for the ACoE to enable the detection of etiologic diagnoses.

## Supplementary material

10.2196/67446 Multimedia Appendix 1 Additional material.

10.2196/67446 Checklist 1 CONSORT-EHEALTH (Consolidated Standards of Reporting Trials of Electronic and Mobile Health Applications and Online Telehealth) checklist.

## Abbreviations

ACE-3: Addenbrooke Cognitive Examination-3
ACoE: Autonomous Cognitive Examination
AUROC: area under the receiver operating characteristic curve
CONSORT: Consolidated Standards of Reporting Trials
CONSORT-EHEALTH: Consolidated Standards of Reporting Trials of Electronic and Mobile Health Applications and Online Telehealth
DCA: Digital cognitive assessment
ICC: intraclass correlation coefficient
MoCA: Montreal Cognitive Assessment
