# Supplementary material for: Machine Learning–Based Cognitive Assessment With The Autonomous Cognitive Examination: Randomized Controlled Trial
Source: J Med Internet Res. 2025 Jul 30;27:e67446. doi: 10.2196/67446 (PMC12310151; doi:10.2196/67446)
Supplement: Multimedia Appendix 1 [file jmir-v27-e67446-s001.docx]

Table S1. **Randomized Controlled Trial Cohort Characteristics.**

| Characteristic | Group 1 | Group 2 |
| --- | --- | --- |
| Average age (years), median (IQR) | 46.3 (46) | 44.2 (39) |
| Age categories (years), N (%) |  |  |
| Under 25 | 0 (0) | 0 (0) |
| 25-45 | 9 (47.5) | 10 (62) |
| 45-65 | 9 (47.5) | 3 (18) |
| Over 65 | 1 (5) | 3 (18) |
| Sex, N (%) |  |  |
| Male | 8 (42) | 9 (56) |
| Female | 11 (58) | 7 (44) |
| Ethnicity, N (%) |  |  |
| White | 12 (64) | 7 (43) |
| Indigenous | 3 (16) | 2 (13) |
| Indian | 1 (5) | 3 (18) |
| Filipino | 1 (5) | 2 (13) |
| African | 1 (5) | 0 (0) |
| Eastern European | 1 (5) | 2 (13) |
| Education, N (%) |  |  |
| Less than Secondary | 0 (0) | 2 (12.5) |
| Secondary | 15 (79) | 12 (75) |
| Post-Secondary | 3 (16) | 2 (12.5) |
| Graduate or Professional | 1 (5) | 0 (0) |
| Employment Status, N (%) |  |  |
| Unemployed | 7 (37) | 4 (25) |
| Employed | 12 (63) | 12 (75) |
| Diagnosis, N (%) |  |  |
| Neurologically Healthy | 5 (26) | 6 (38) |
| Mild Cognitive Impairment | 4 (21) | 3 (18) |
| Probable Alzheimer Disease | 2 (11) | 1 (6) |
| Epilepsy | 8 (42) | 6 (38) |

Table S2. **Exam characteristics.** Each of the 19 primary questions is listed on the left, with the specific content of the question and the cognitive domain it is attributable to.

| Question Number | Maneuver Name | Maneuver Description | Domain |
| --- | --- | --- | --- |
| 1 | Copy & Draw | Object copy and clock drawing. | Visuospatial |
| 2 | Immediate 3-word recall | Immediate recall of 3 words. | Attention |
| 3 | Serial 7’s | Serial subtraction of 7 from 100. | Attention |
| 4 | Delayed 3-word recall | Delayed recall of 3 words. | Memory |
| 5 | Phonemic & semantic list generation | Phonemic and semantic list generation. | Fluency |
| 6 | Immediate 7-word recall | Immediate recall of 7 words. | Memory |
| 7 | Semantic recall | Recall of semantic information. | Memory |
| 8 | Commands | Command-based comprehension. | Language |
| 9 | Writing | Written sentence. | Language |
| 10 | Word repetition | Single word repetition. | Language |
| 11 | Sentence repetition | Sentence repetition. | Language |
| 12 | Spoken naming | Object naming. | Language |
| 13 | Naming | Object comprehension. | Language |
| 14 | Dyslexia testing | Read words aloud. | Language |
| 15 | Dot counting | Visual object counting. | Visuospatial |
| 16 | Incomplete letters | Obscured letter identification. | Visuospatial |
| 17 | Temporal orientation | State the date | Attention |
| 18 | Spatial orientation | State physical location | Attention |
| 19 | Delayed 7-word recall | Recognition of 7 words. | Memory |

Table S3.  **Question 1 of the ACoE.** Visuospatial function tested by the SketchNet convolutional neural network.

| Name | Task | Example | Preprocessing | Processing | Scoring |
| --- | --- | --- | --- | --- | --- |
| Copy | The patient must copy overlapping infinities | 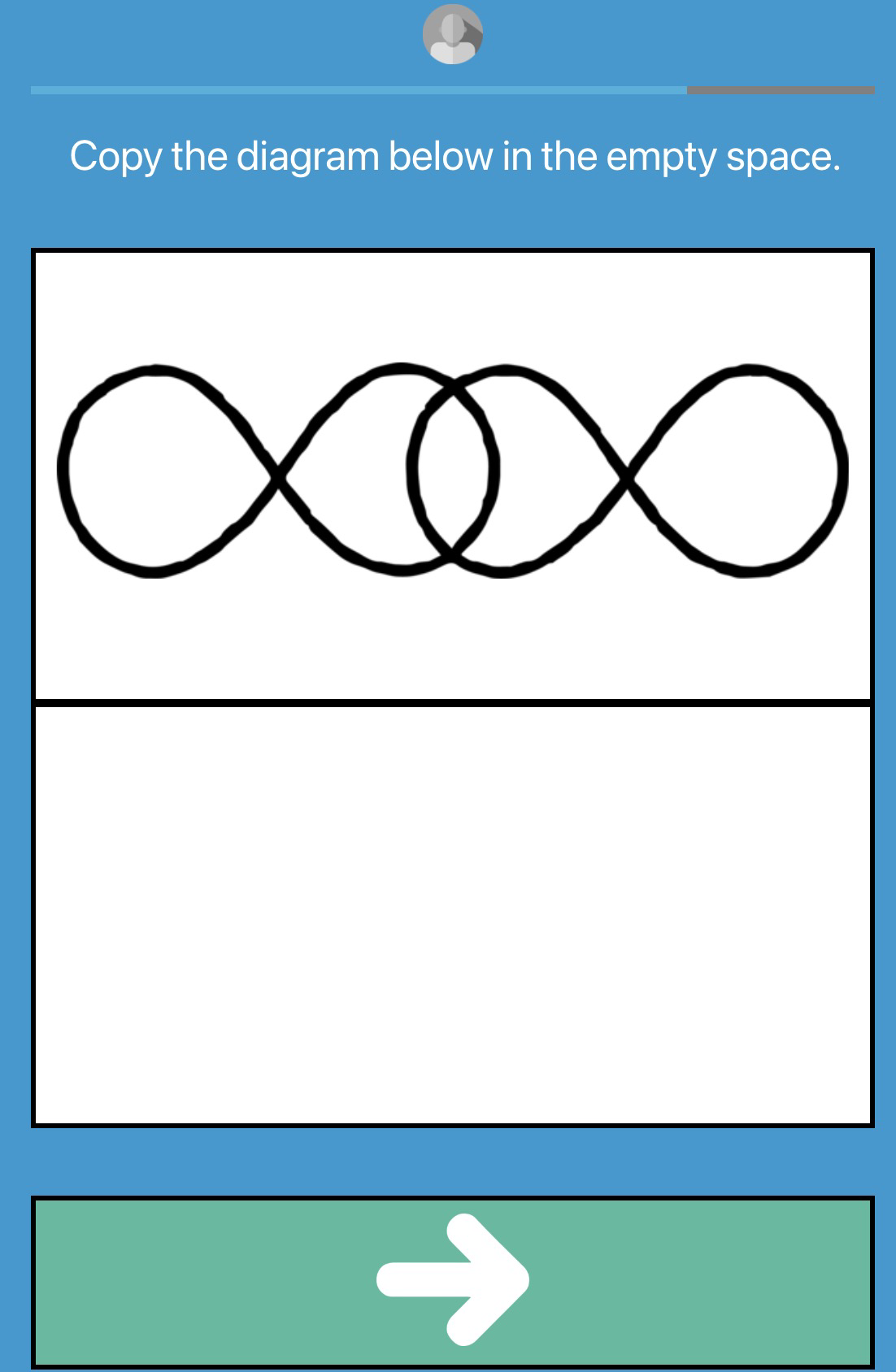 | PNG of resultant drawing is cropped, placed on white background, normalized in size, normalized in colour. | Passed through convolutional neural network which detects infinity/clock/cube. | 0 or 1  Points |
| Copy | The patient must copy a cube wire diagram | 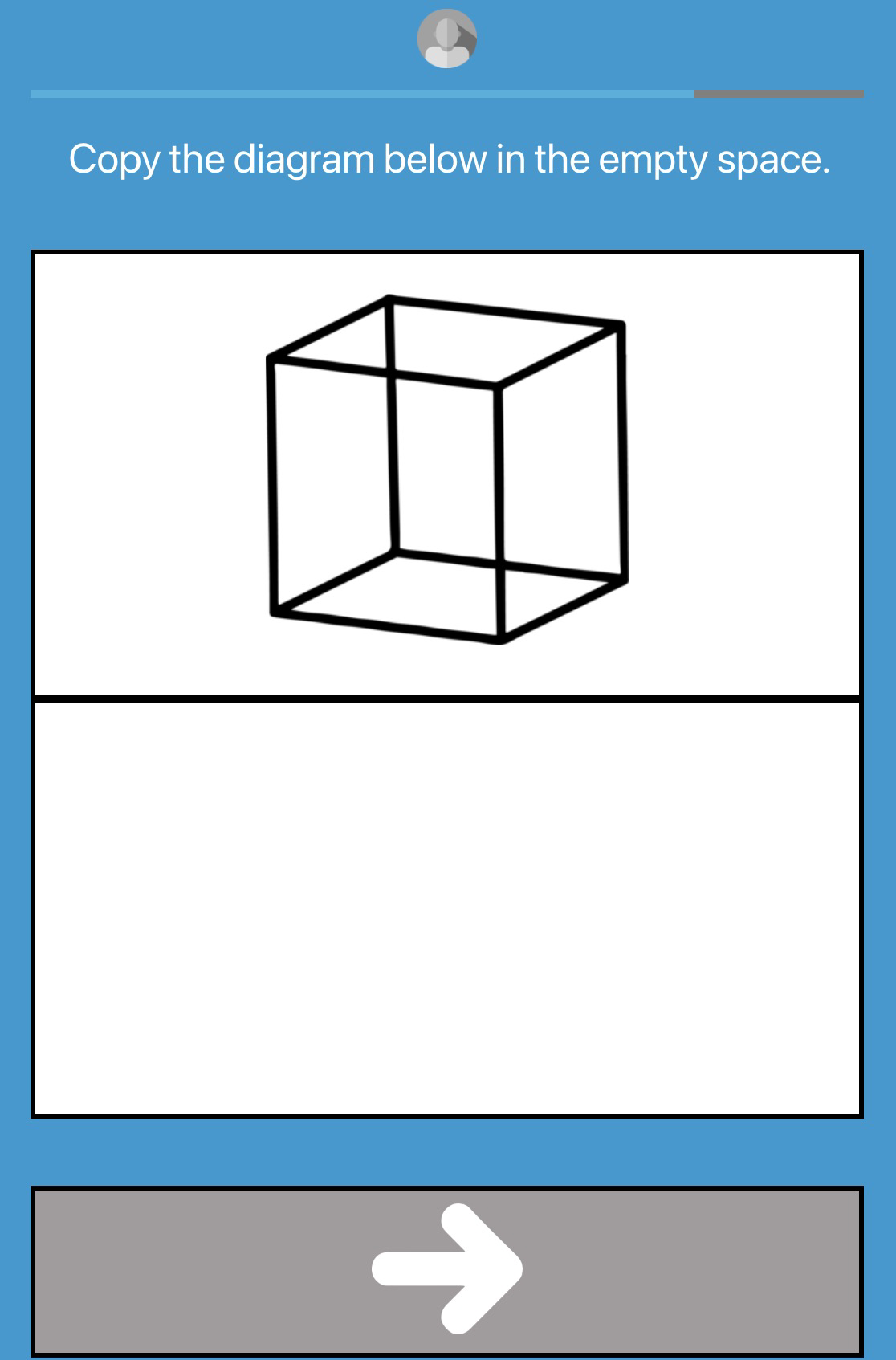 | PNG of resultant drawing is cropped, placed on white background, normalized in size, normalized in colour. | Passed through convolutional neural network which detects infinity/clock/cube. | 0 or 2  points |
| Draw | The patient must draw a clock with all hands and numbers at 10-past-5 | 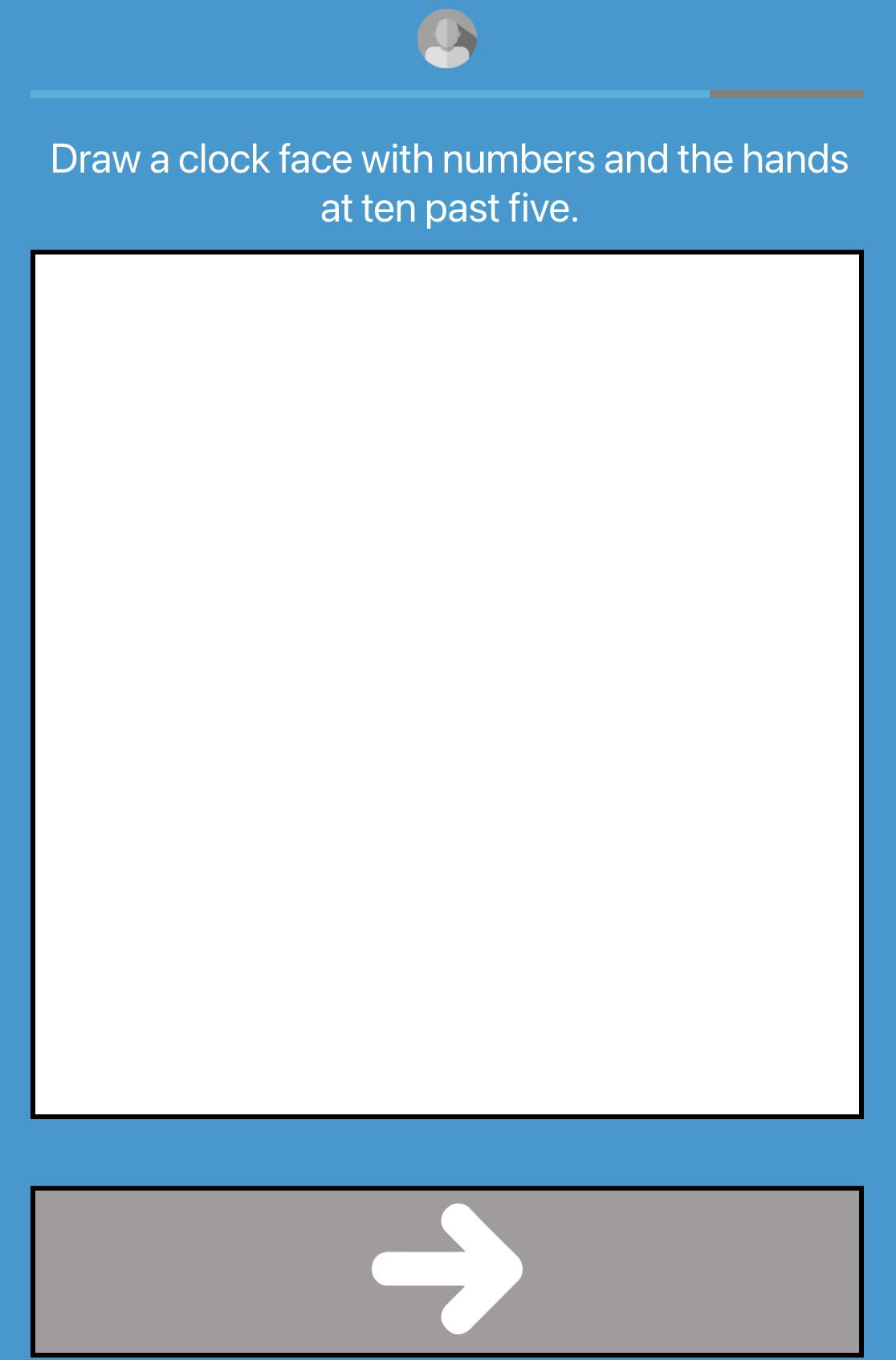 | PNG of resultant drawing is cropped, placed on white background, normalized in size, normalized in colour. | Passed through convolutional neural network which detects infinity/clock/cube. | 0 or 5 points |

Table S4. **Question 2 of the ACoE.** Executive function, tested by natural language processing

| Name | Task | Example | Preprocessing | Processing | Scoring |
| --- | --- | --- | --- | --- | --- |
| 3-word repetition | The patient must listen to 3 spoken words, click the microphone, and state them. | 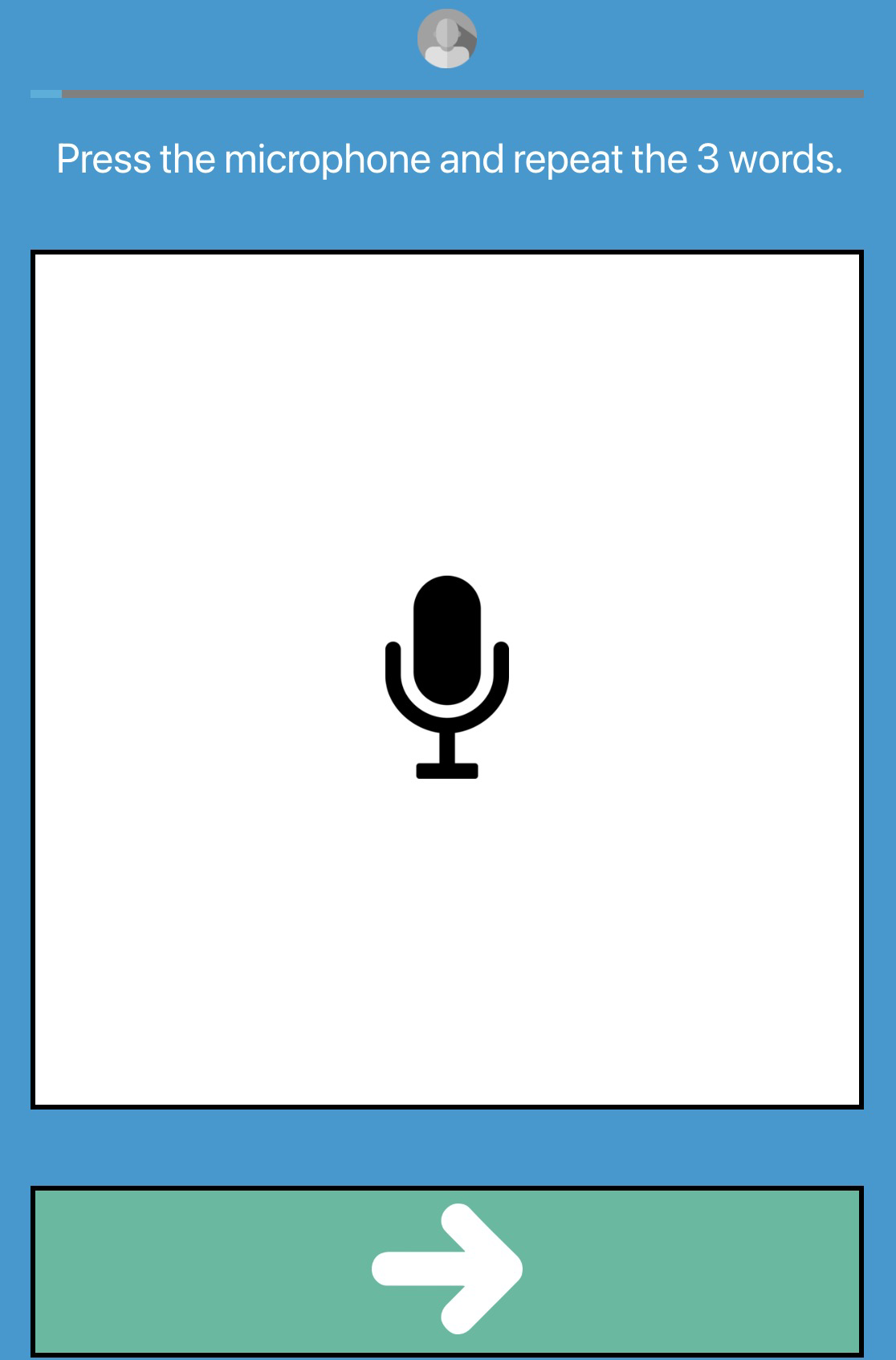 | MP4 uploaded.  FFMPEG for preprocessing.  1s pre and post.  Normalized to background noise.  Bitrate normalized.  Upsampled. | Passed through NLP Speech Recognition  Python Package.  A normative distribution of acceptable words are compared to the detected words. | 0 to 3  Points  1 point per word |

Table S5. **Question 3 of the ACoE.** Executive function, tested by natural language processing

| Name. | Task | Example | Preprocessing | Processing | Scoring |
| --- | --- | --- | --- | --- | --- |
| Serial  7s | The patient must click the microphone, subtract 7 from 100 and state the answers out loud. | 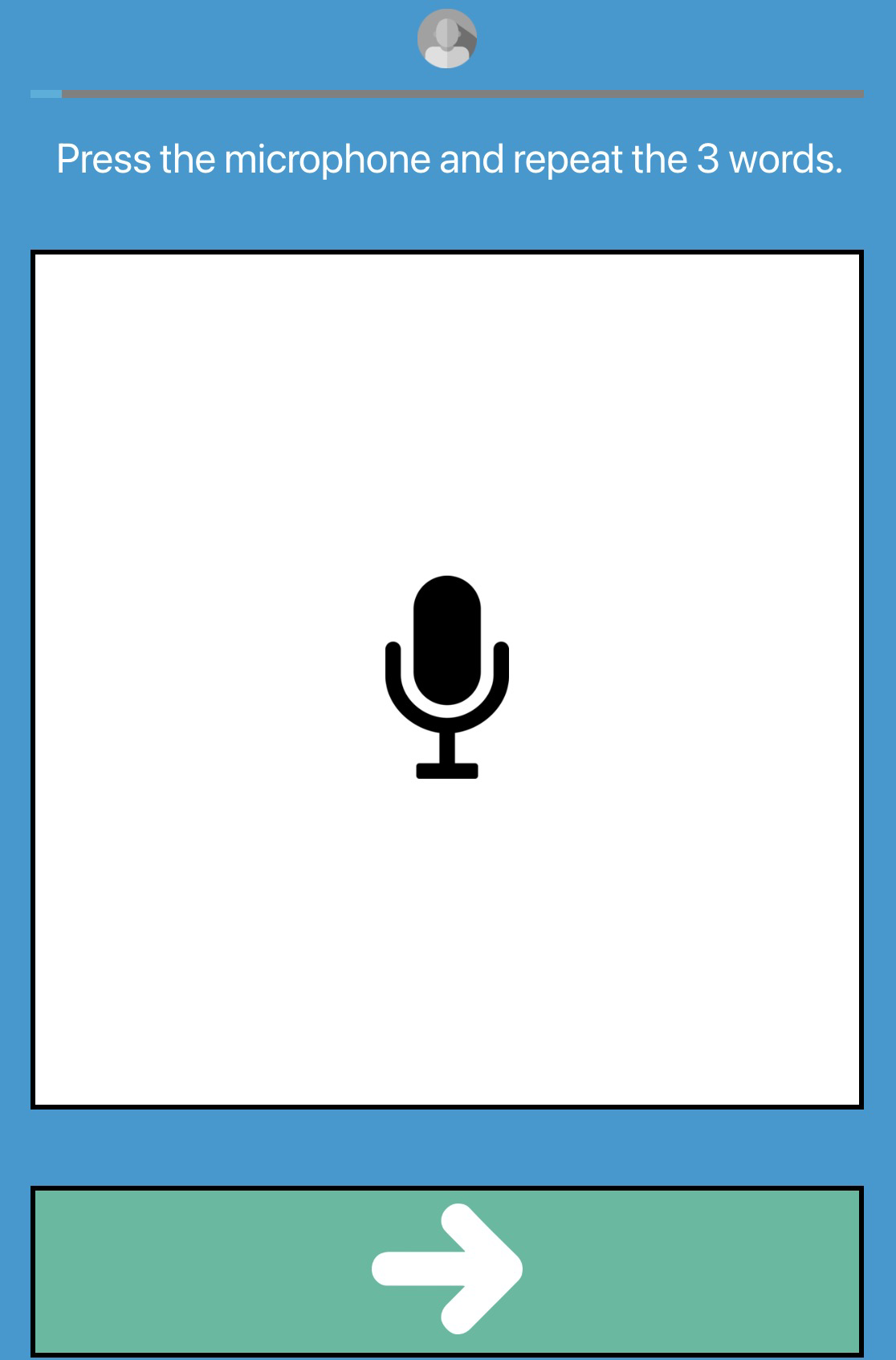 | MP4 uploaded.  FFMPEG for preprocessing.  1s pre and post.  Normalized to background noise.  Bitrate normalized.  Upsampled. | Passed through NLP Speech Recognition  Python Package.  The spoken numbers are recognized, and successful subtractions of 7 are calculated. | 0 to 5  Points  1 point per successful subtraction of 7. Can be correct from an incorrect number. |

Table S6. **Question 4 of the ACoE.** Memory function, tested by natural language processing

| Name | Task | Example | Preprocessing | Processing | Scoring |
| --- | --- | --- | --- | --- | --- |
| Delayed word recall | The patient must press the microphone and state the 3 words from prior. | 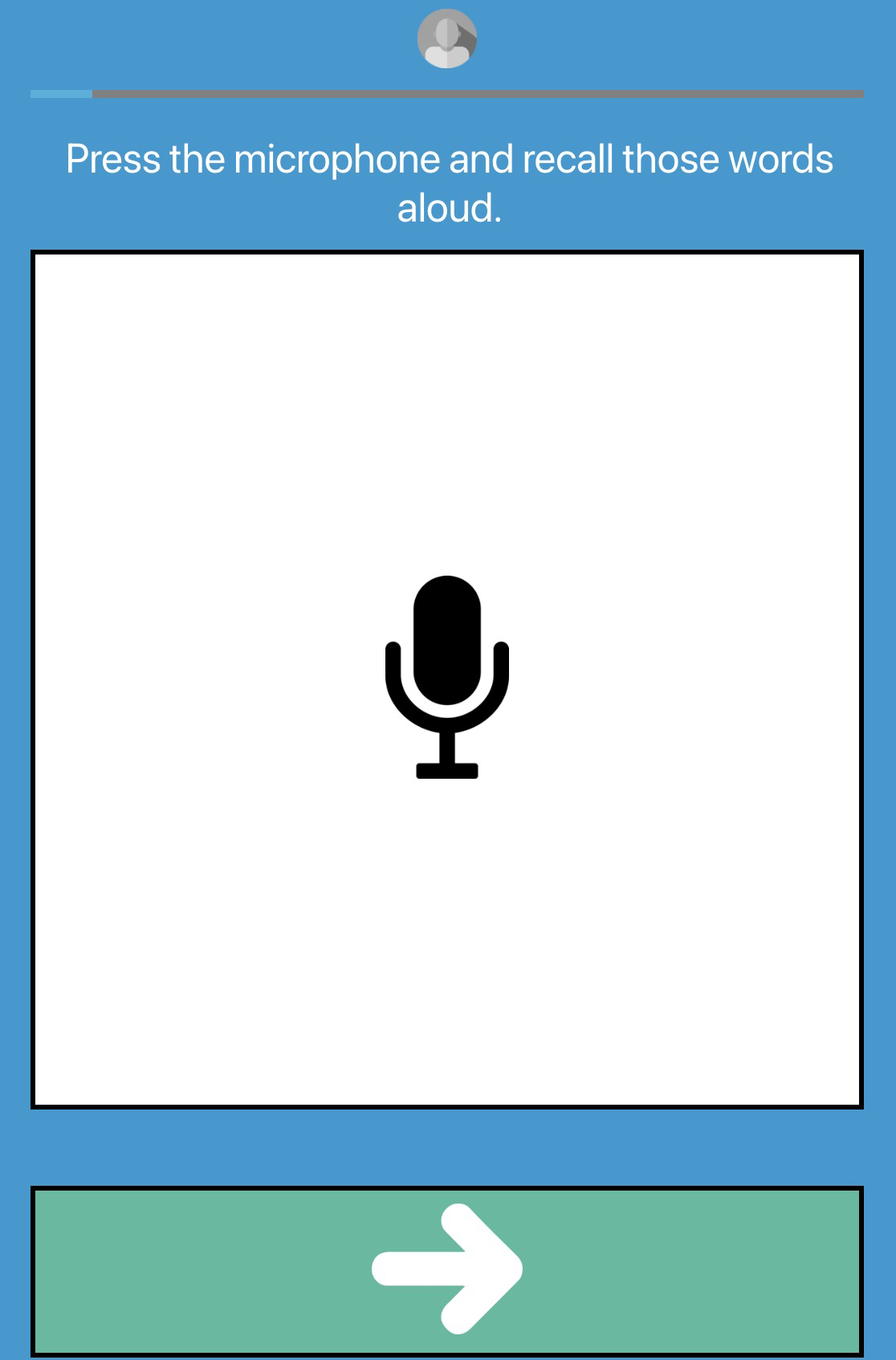 | MP4 uploaded.  FFMPEG for preprocessing.  1s pre and post.  Normalized to background noise.  Bitrate normalized.  Upsampled. | Passed through NLP Speech Recognition  Python Package.  The spoken words are compared to a normative distribution of acceptable words. | 0 to 3  Points  1 point per successful word. |

Table S7. **Question 5 of the ACoE.** Language function, subdomain fluency, tested by natural language processing

| Name | Task | Example | Preprocessing | Processing | Scoring |
| --- | --- | --- | --- | --- | --- |
| Phonemic List | The patient must press the microphone and name as many words starting with P as possible.  60s limit. | 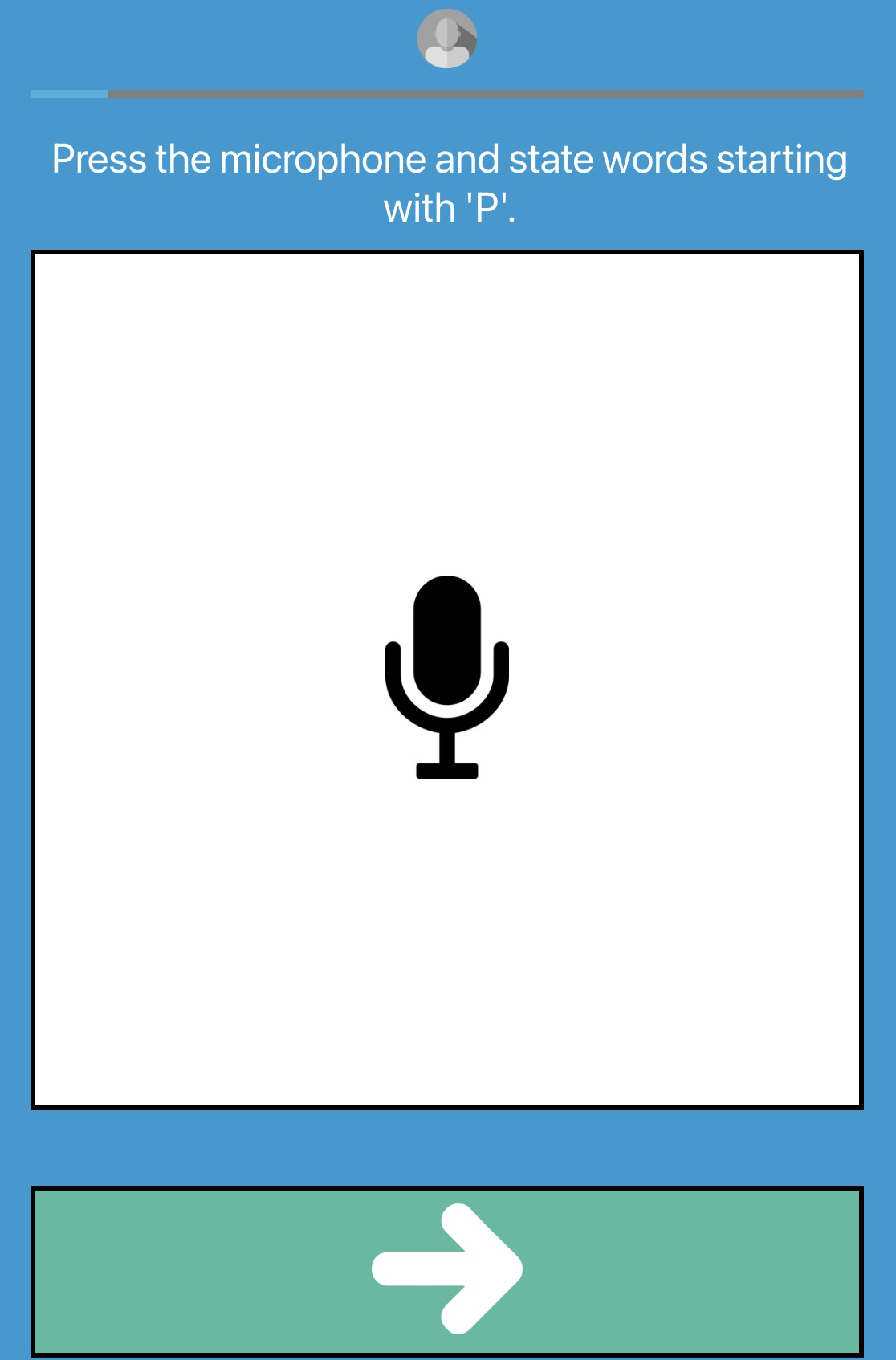 | MP4 uploaded.  FFMPEG for preprocessing.  1s pre and post.  Normalized to background noise.  Bitrate normalized.  Upsampled. | Passed through NLP Speech Recognition  Python Package.  Natural language processing toolkit compares spoken words to all acceptable words. | 0-7 points  In brackets of words, from 0 correct to >18  correct |
| Semantic List | Press mic.  Name animals in 60s. | 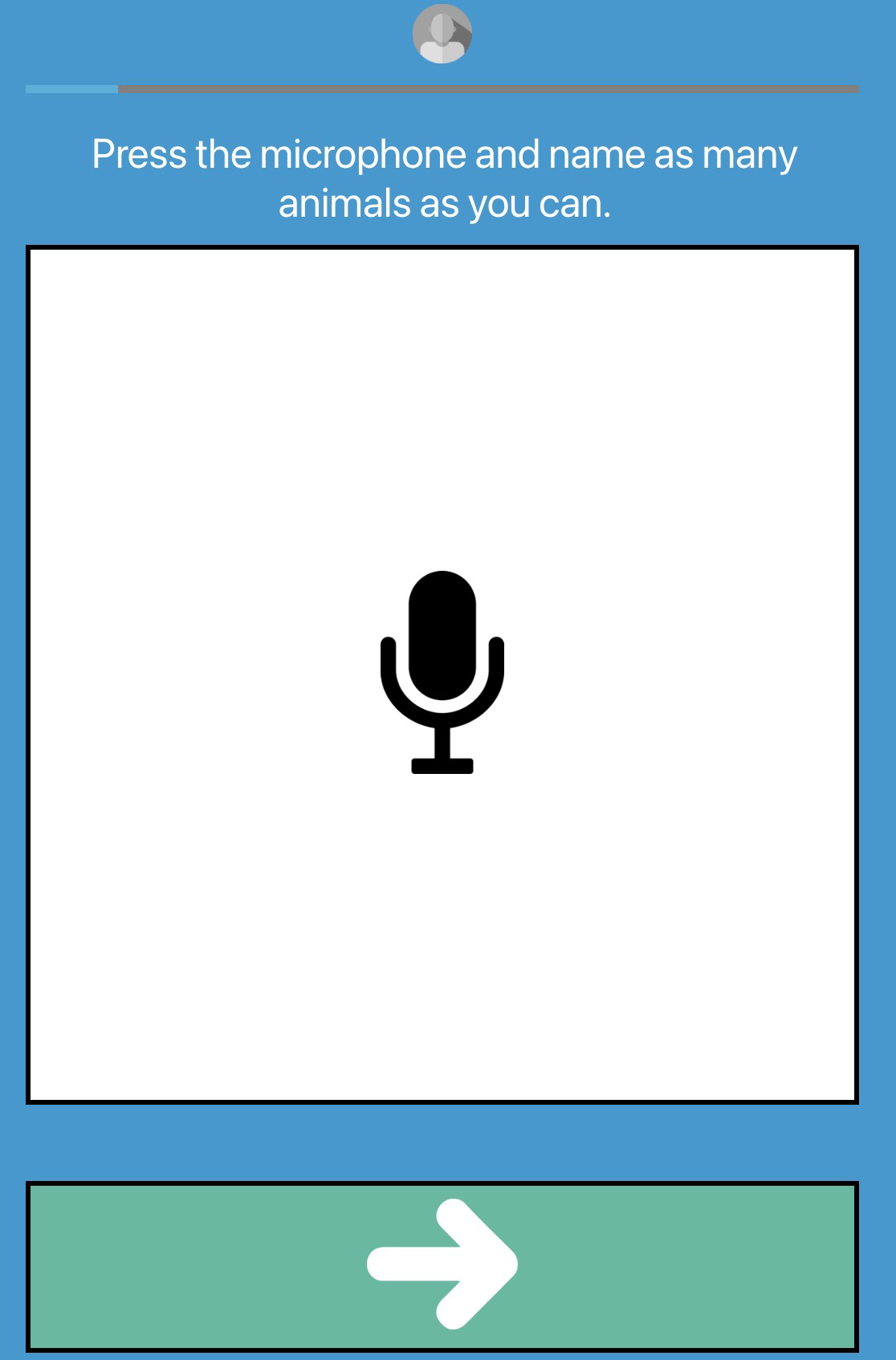 | MP4 uploaded.  FFMPEG for preprocessing.  1s pre and post.  Normalized to background noise.  Bitrate normalized.  Upsampled. | Passed through NLP Speech Recognition  Python Package.  The spoken words are compared to a normative distribution of acceptable words. | 0-7 points  Brackets  0 words  To  >22 words |

Table S8. **Question 6 of the ACoE.** Memory function, tested by natural language processing

| Name | Task | Example | Preprocessing | Processing | Scoring |
| --- | --- | --- | --- | --- | --- |
| Immediate recall | Press microphone  State 7-part foreign address.  2 entrains.  1 test. | 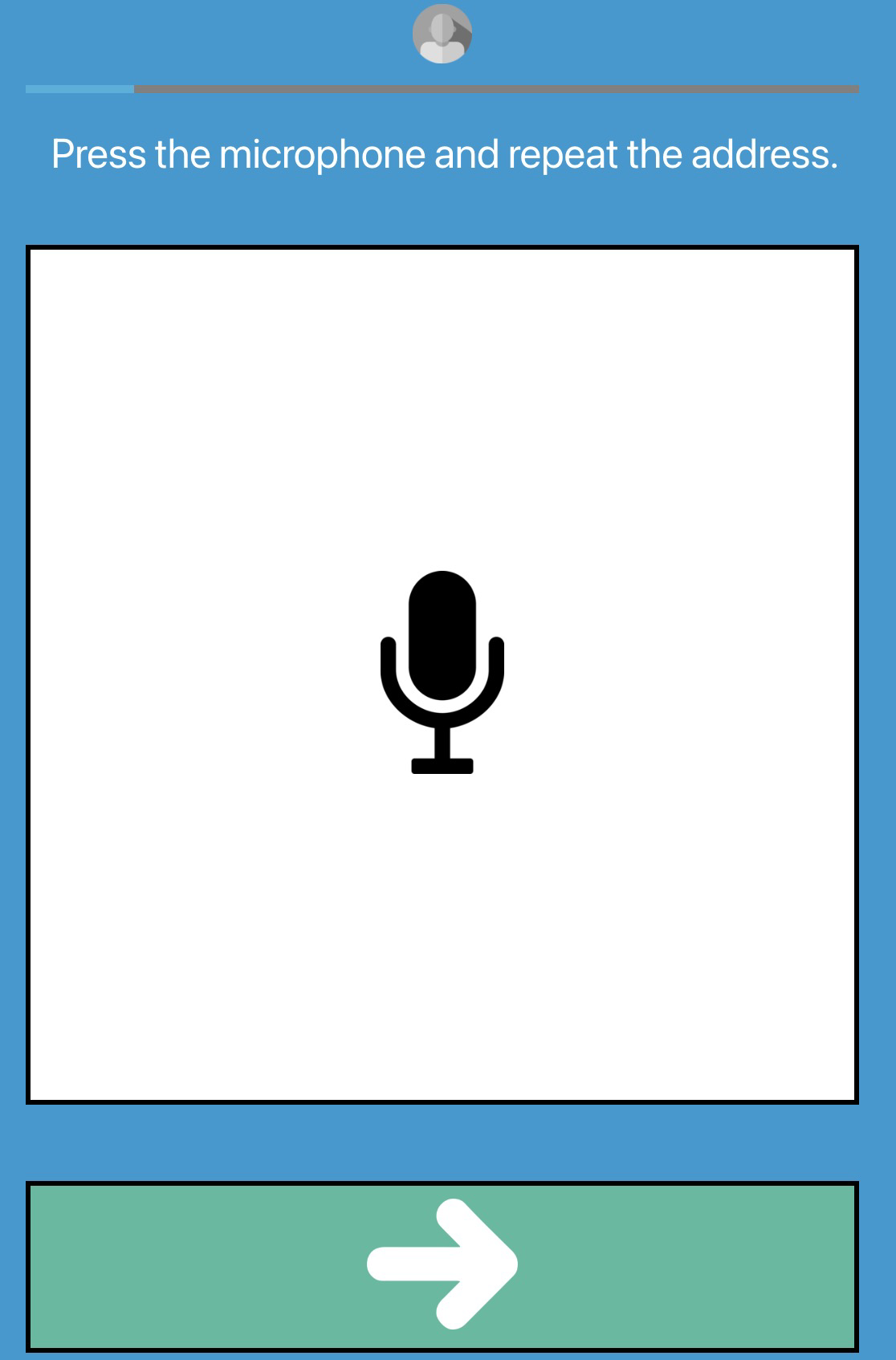 | MP4 uploaded.  FFMPEG for preprocessing.  1s pre and post.  Normalized to background noise.  Bitrate normalized.  Upsampled. | Passed through NLP Speech Recognition  Python Package.  Compare detected words to normative list of acceptable words. | 0-7 points.  1 point per part |

Table S9. **Question 7 of the ACoE.** Memory function, subdomain semantic memory, tested by natural language processing

| Name | Task | Example | Preprocessing | Processing | Scoring |
| --- | --- | --- | --- | --- | --- |
| Semantic  Recall A | State current Canadian Prime Minister | 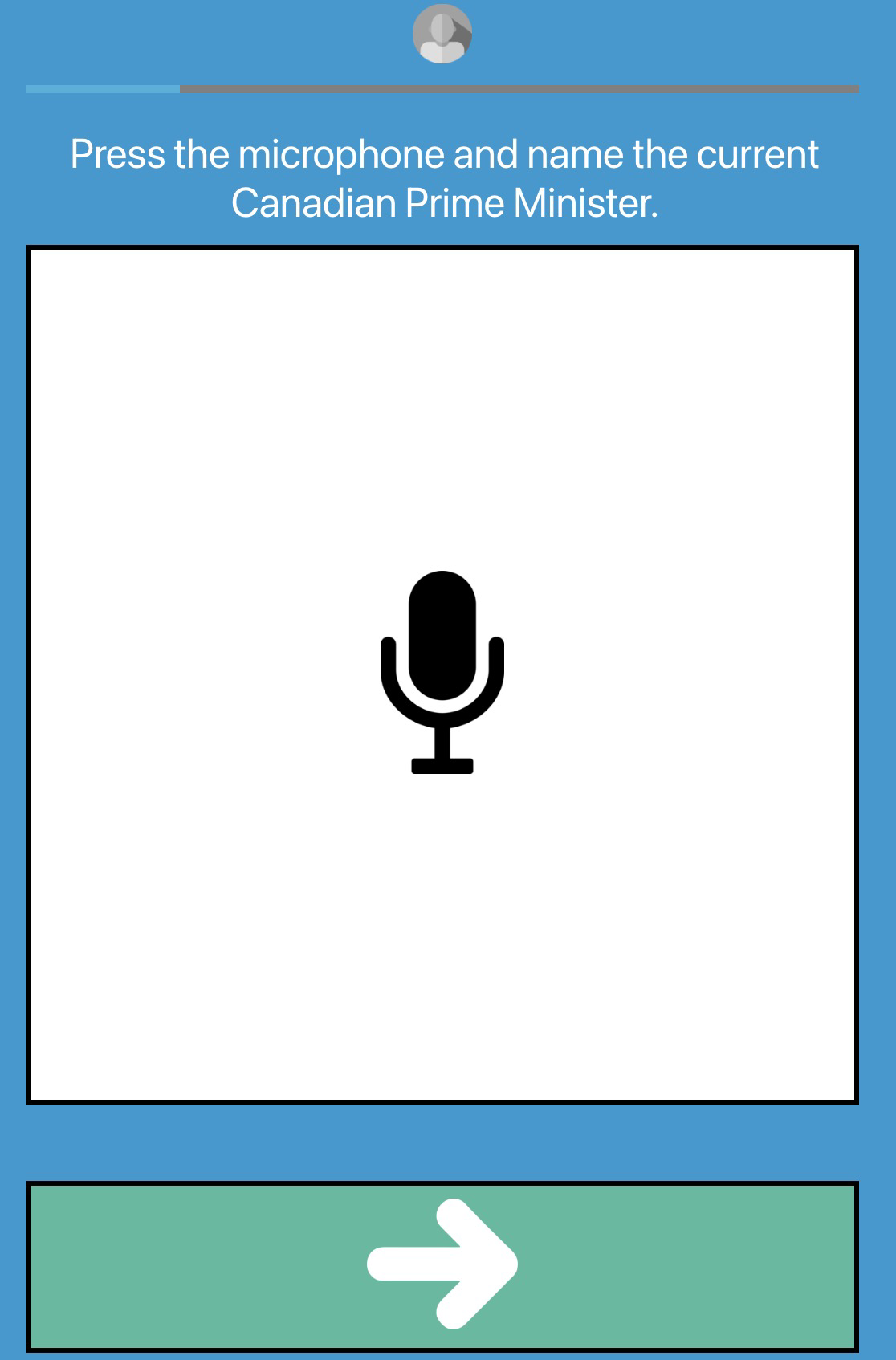 | MP4 uploaded.  FFMPEG for preprocessing.  1s pre and post.  Normalized to background noise.  Bitrate normalized.  Upsampled. | Passed through NLP Speech Recognition  Python Package.  Compare detected words to normative list of acceptable words. | 0 -1 points.  1 point per part |
| Semantic Recall B | State first Canadian Prime Minister | As above. | As above. | As above. | As above. |
| Semantic Recall C | State first African American US President | As above. | As above. | As above. | As above. |
| Semantic Recall D | State Current US President | As above. | As above. | As above. | As above. |

Table S10. **Question 8 of the ACoE.** Language function, subdomain comprehension, tested by expert algorithm.

| Name | Task | Example | Preprocessing | Processing | Scoring |
| --- | --- | --- | --- | --- | --- |
| Instruction A | Move pencil then paper | 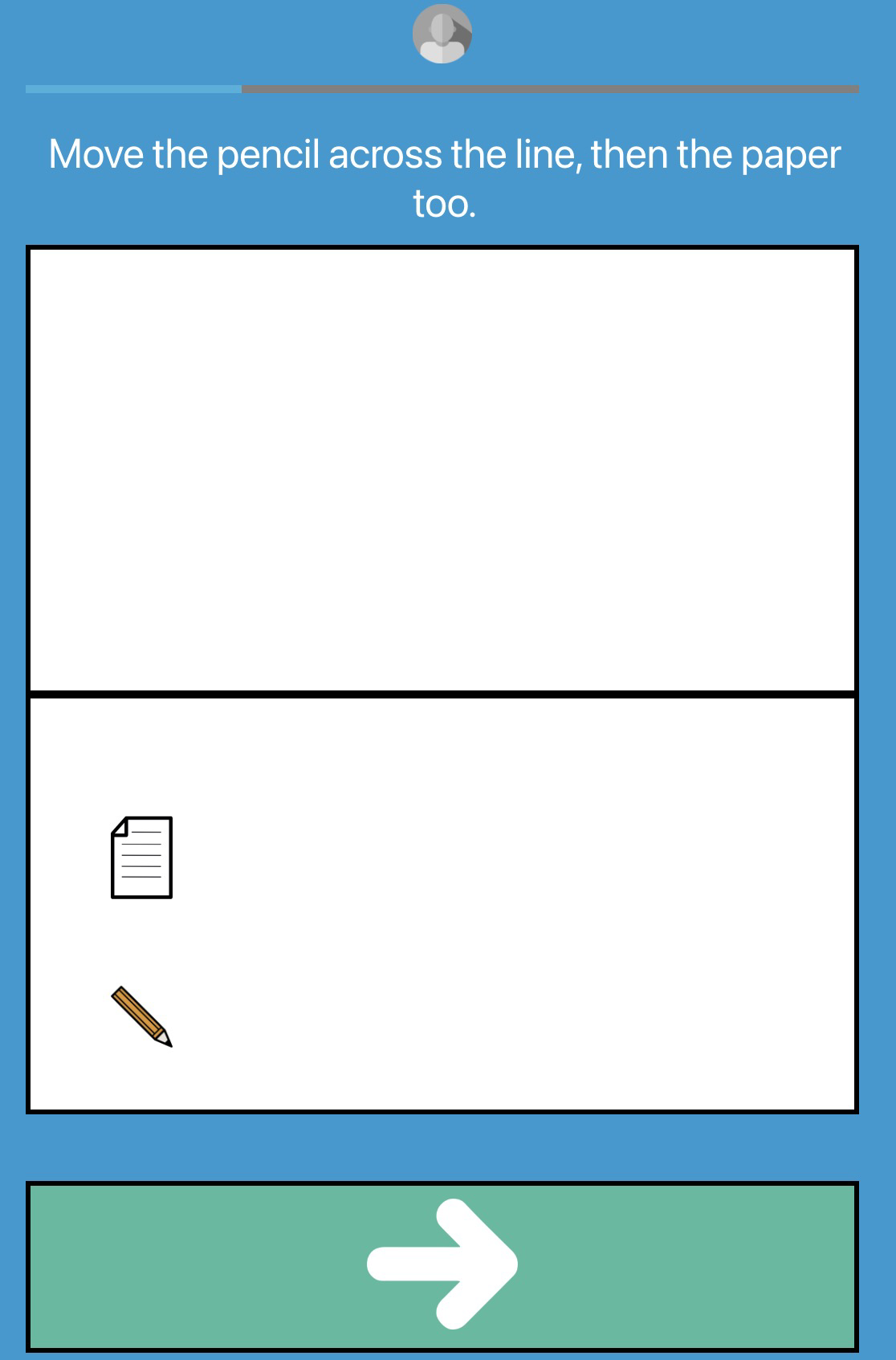 | Track vector of image movement. Upload raw vector. | Compare vector movement to bounding-boxes calculated from patient screen size.  Evaluate if vectors comply with instruction | 0 points.  Training. |
| Instruction B | Place paper on pencil. | As above. | As above. | As above. | 1 point |
| Instruction C | Move pencil across line, but not paper. | As above. | As above. | As above. | 1 point |
| Instruction D | Move pencil across line after touching paper. | As above. | As above. | As above. | 1 point. |

Table S11. **Question 9 of the ACoE.** Language function, subdomain writing, tested by natural language processing.

| Name | Task | Example | Preprocessing | Processing | Scoring |
| --- | --- | --- | --- | --- | --- |
| Writing | Write 2 complete sentences. | 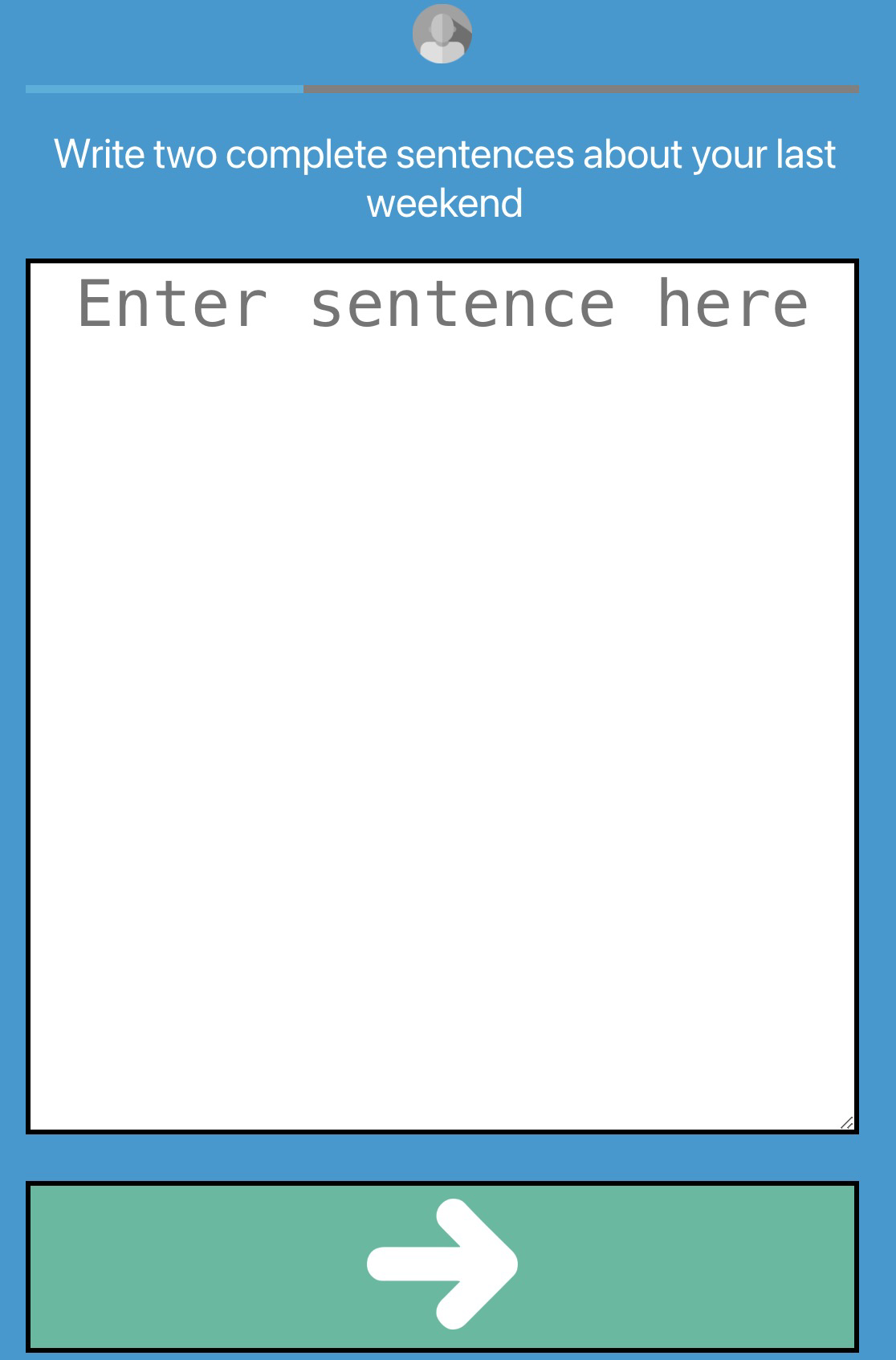 | Nil. | Natural language processing of raw text.  Detect 4 parts: punctuation  Capitalization  Grammar  2 sentences. | 0-2 points,  0.5 points per correct part. |

Table S12. **Question 10 of the ACoE.** Language function, subdomain repetition, tested by natural language processing.

| Name | Task | Example | Preprocessing | Processing | Scoring |
| --- | --- | --- | --- | --- | --- |
| Repetition A | Repeat caterpillar | 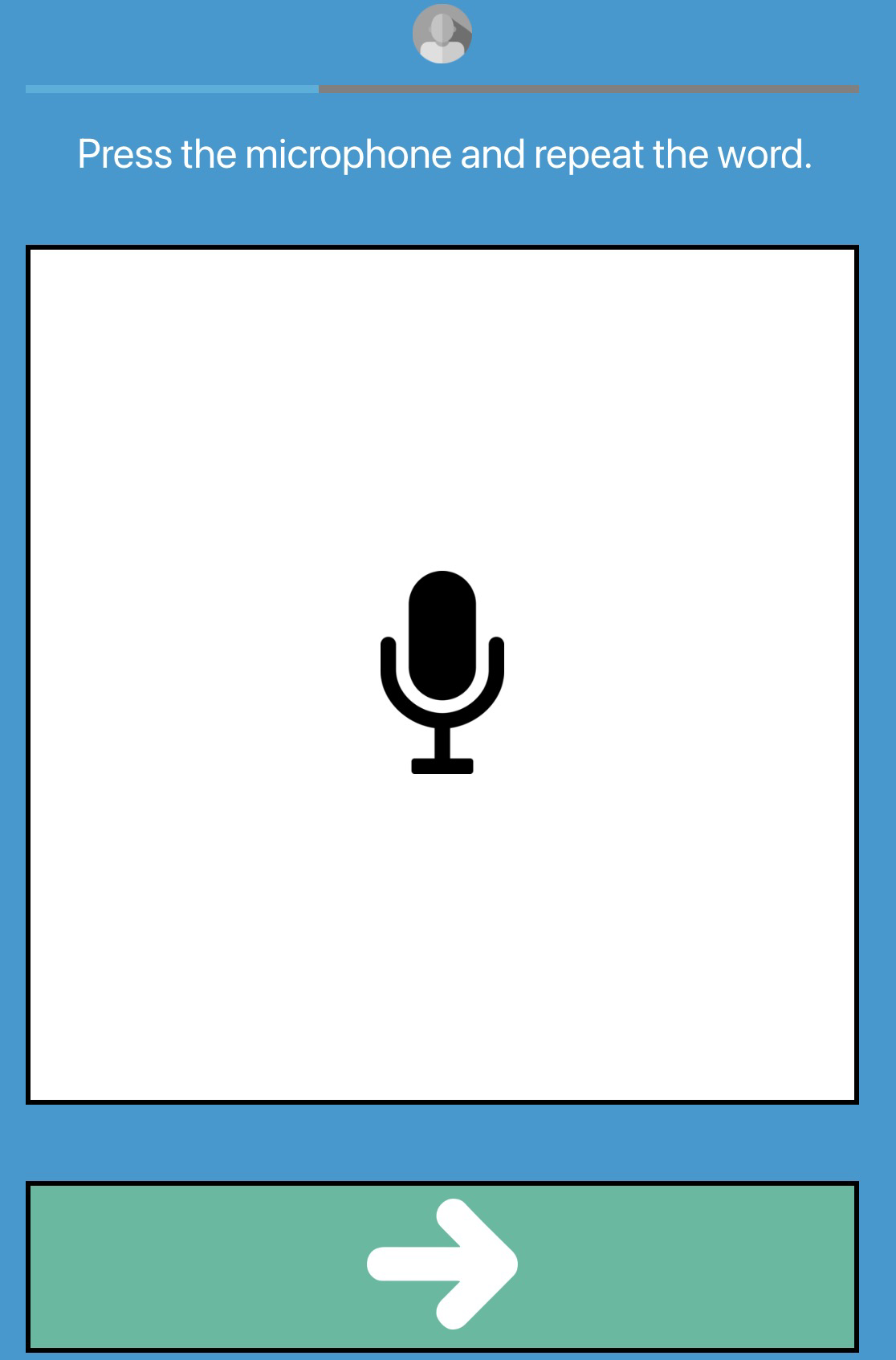 | MP4 uploaded.  FFMPEG for preprocessing.  1s pre and post.  Normalized to background noise.  Bitrate normalized.  Upsampled. | Passed through NLP Speech Recognition  Python Package.  Compare detected words to normative list of acceptable words. | - 1. Points   1 point per correct word. |
| Repetition B | Repeat statistician | As above. | As above. | As above. | As above |
| Repetition C | Repeat eccentricity | As above. | As above. | As above. | As above |
| Repetition C | Repeat unintelligible | As above. | As above. | As above. | As above |

Table S13. **Question 11 of the ACoE.** Language function, subdomain repetition, tested by natural language processing.

| Name | Task | Example | Preprocessing | Processing | Scoring |
| --- | --- | --- | --- | --- | --- |
| Repetition A | Repeat “all that glitters is not gold” | 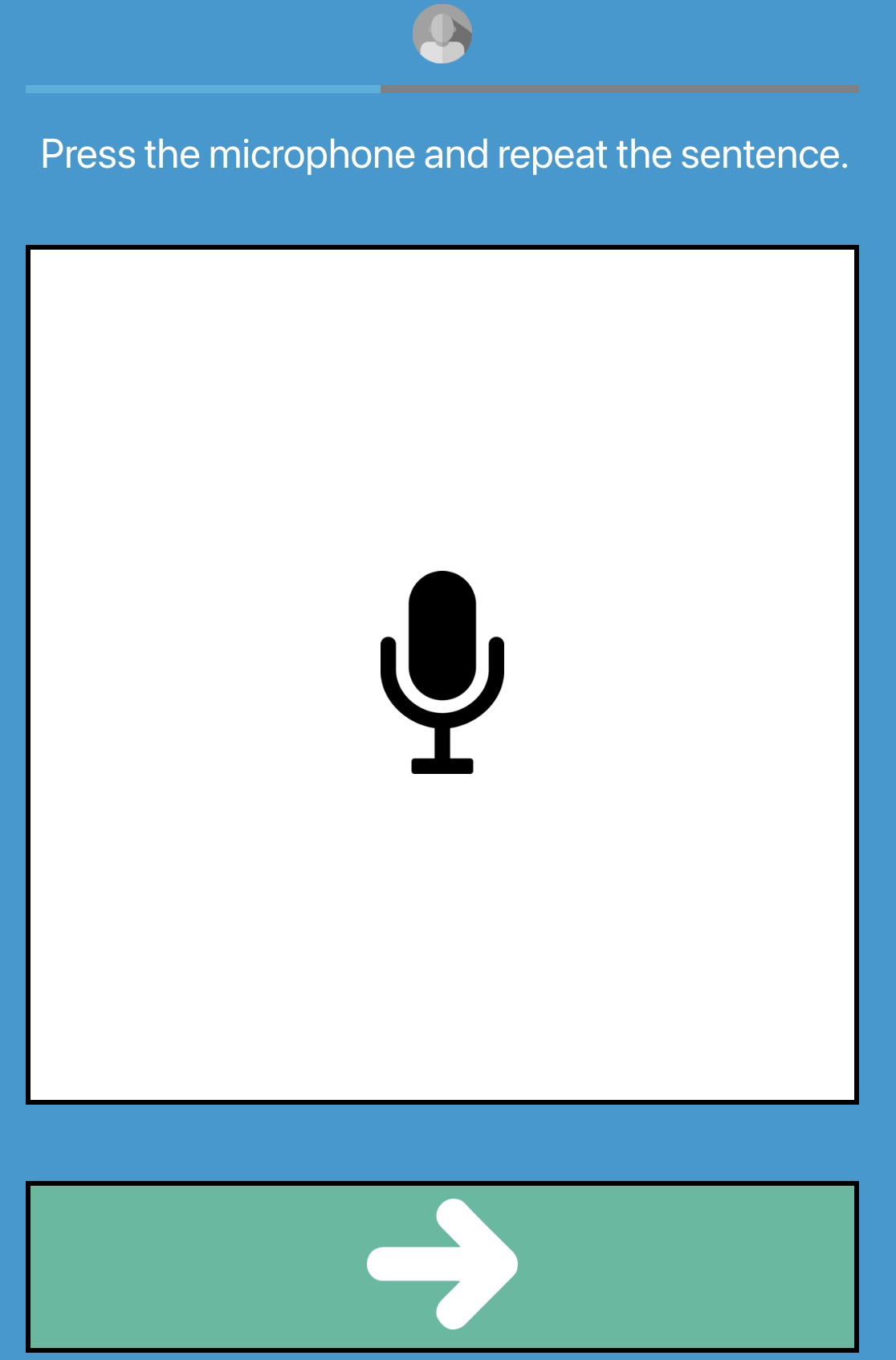 | MP4 uploaded.  FFMPEG for preprocessing.  1s pre and post.  Normalized to background noise.  Bitrate normalized.  Upsampled. | Passed through NLP Speech Recognition  Python Package.  Compare detected words to normative list of acceptable sentences. | 0-1Points  1 point per correct sentence. |
| Repetition B | Repeat “a stitch in time saves nine” | As above. | As above. | As above. | As above |

Table S14. **Question 12 of the ACoE.** Language function, subdomain naming, tested by natural language processing.

| Name | Task | Example | Preprocessing | Processing | Scoring |
| --- | --- | --- | --- | --- | --- |
| Naming A | Name spoon | 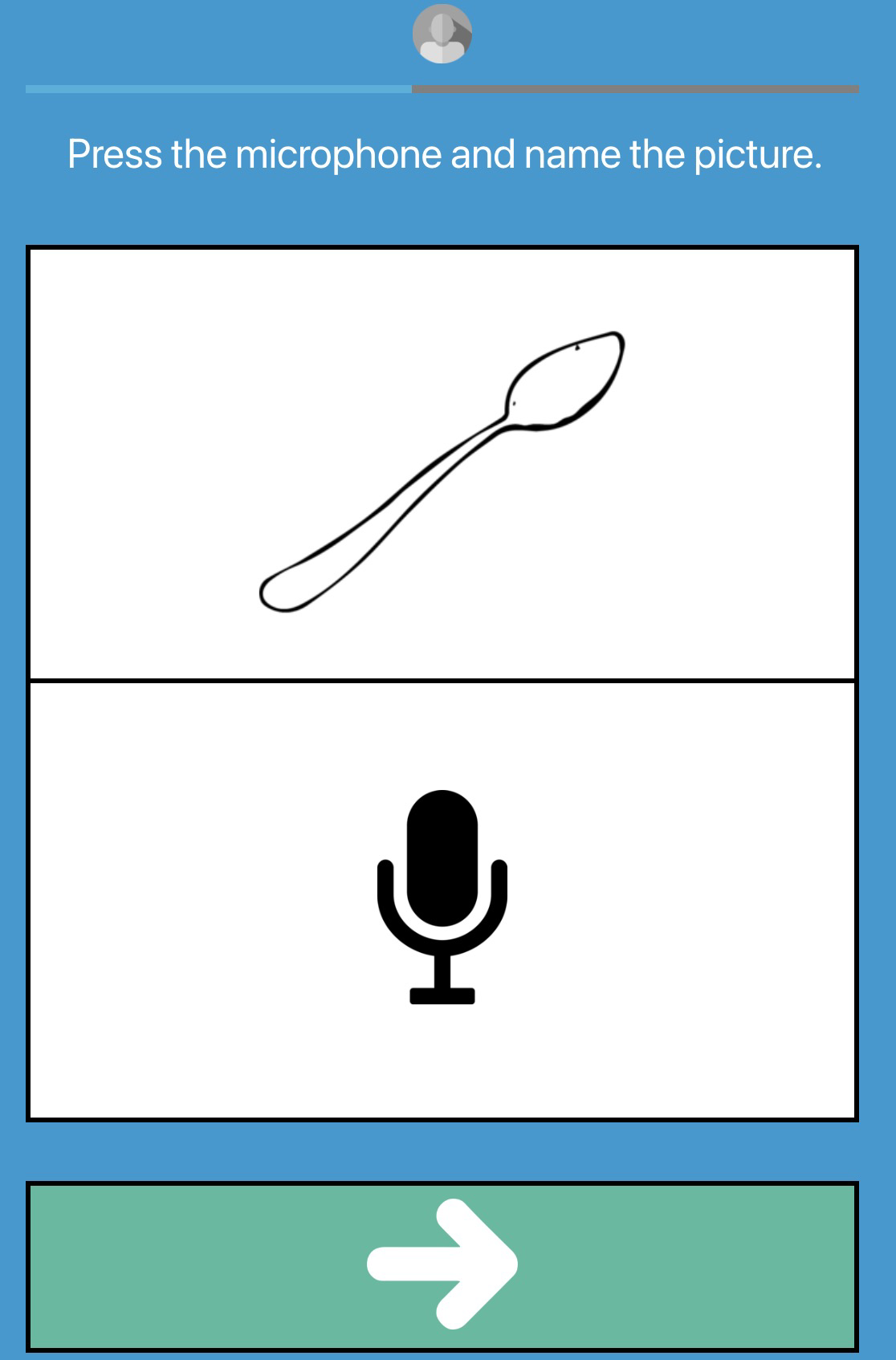 | MP4 uploaded.  FFMPEG for preprocessing.  1s pre and post.  Normalized to background noise.  Bitrate normalized.  Upsampled. | Passed through NLP Speech Recognition  Python Package.  Compare detected words to normative list of acceptable words. | 0-1 Points  1 point per correct word. |
| Naming B | Name book | Similar to above. | As above. | As above. | As above |
| Naming C | Name kangaroo | Similar to above. | As above. | As above. | As above |
| Naming D | Name penguin | Similar to above. | As above. | As above. | As above |
| Naming E | Name anchor | Similar to above. | As above. | As above. | As above |
| Naming F | Name camel | Similar to above. | As above. | As above. | As above |
| Naming G | Name harp | Similar to above. | As above. | As above. | As above |
| Naming H | Name rhinoceros | Similar to above. | As above. | As above. | As above |
| Naming I | Name barrel | Similar to above. | As above. | As above. | As above |
| Naming J | Name crown | Similar to above. | As above. | As above. | As above |
| Naming K | Name alligator | Similar to above. | As above. | As above. | As above |
| Naming L | Name accordion | Similar to above. | As above. | As above. | As above |

Table S15. **Question 13 of the ACoE.** Language function, subdomain naming, tested by expert algorithm.

| Name | Task | Example | Preprocessing | Processing | Scoring |
| --- | --- | --- | --- | --- | --- |
| Identification A | Touch object associated with monarchy. | 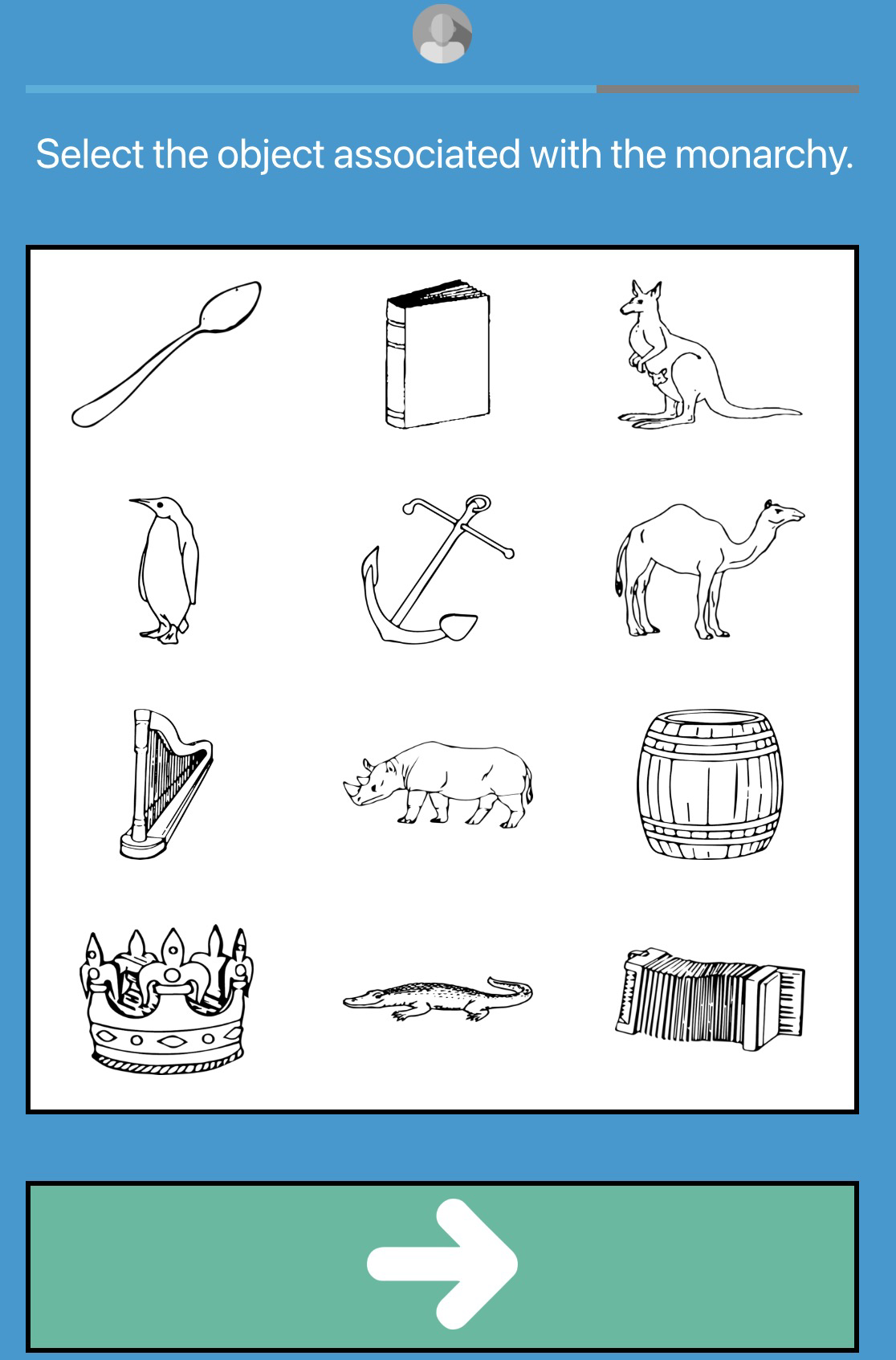 | Set true if calculated bounding box around crown selected. | None. | 0-1 Points  1 point if selected. |
| Identification B | Touch object which is a marsupial | Same as above. | Set true if calculated bounding box around kangaroo selected. | As above. | As above |
| Identification C | Touch object associated with the Antarctic | Same as above. | Set true if calculated bounding box around penguin selected. | As above. | As above |
| Identification D | Touch object with a nautical connection | Same as above. | Set true if calculated bounding box around anchor selected. | As above. | As above |

Table S16. **Question 14 of the ACoE.** Language function, subdomain pronunciation, tested by natural language processing.

| Name | Task | Example | Preprocessing | Processing | Scoring |
| --- | --- | --- | --- | --- | --- |
| Reading | Touch mic and read words out loud. | 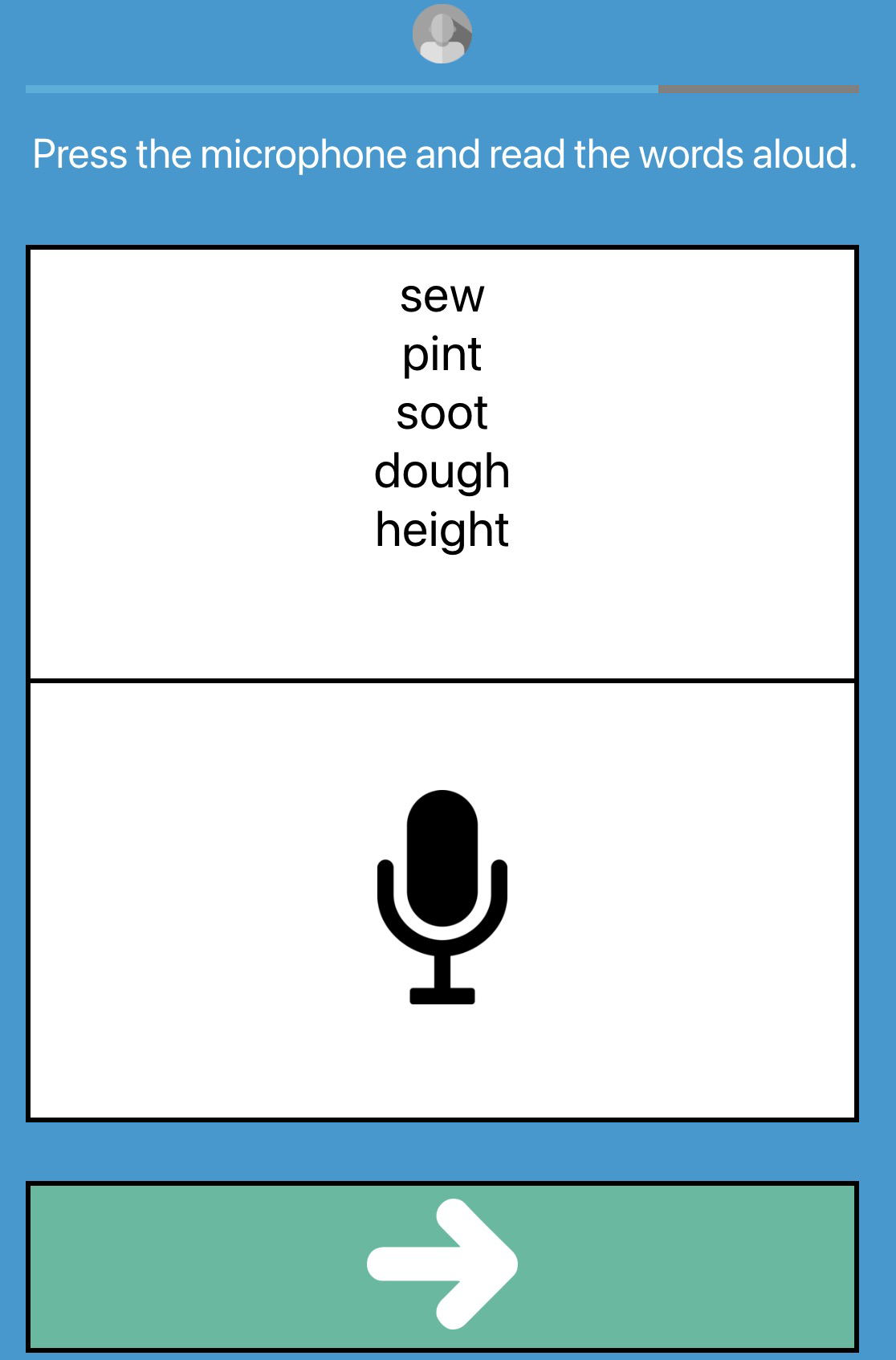 | MP4 uploaded.  FFMPEG for preprocessing.  1s pre and post.  Normalized to background noise.  Bitrate normalized.  Upsampled. | Passed through NLP Speech Recognition  Python Package.  Compare detected words to normative list of acceptable words. | 0-1 Points  1 point if all words correct. |

Table S17. **Question 15 of the ACoE.** Visuospatial function, tested by natural language processing.

| Name | Task | Example | Preprocessing | Processing | Scoring |
| --- | --- | --- | --- | --- | --- |
| Counting A | Count 8 dots out loud.  Do not touch dots. | 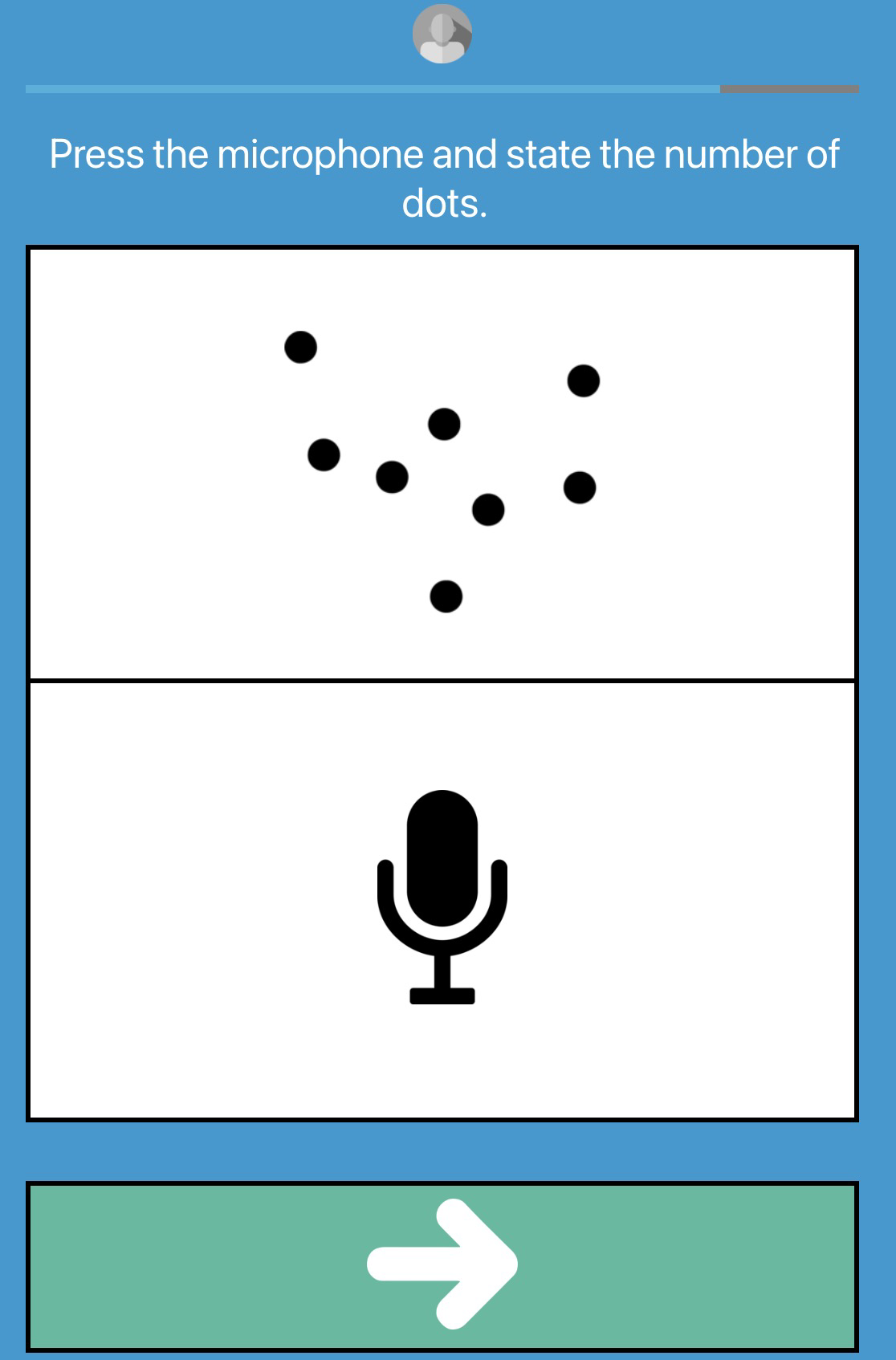 | MP4 uploaded.  FFMPEG for preprocessing.  1s pre and post.  Normalized to background noise.  Bitrate normalized.  Upsampled. | Passed through NLP Speech Recognition  Python Package.  Compare detected count to normative list of acceptable counts. | 0-1 Points  Point if correct dot count.  If dots touched, 0 points |
| Counting B | Count 10 dots | 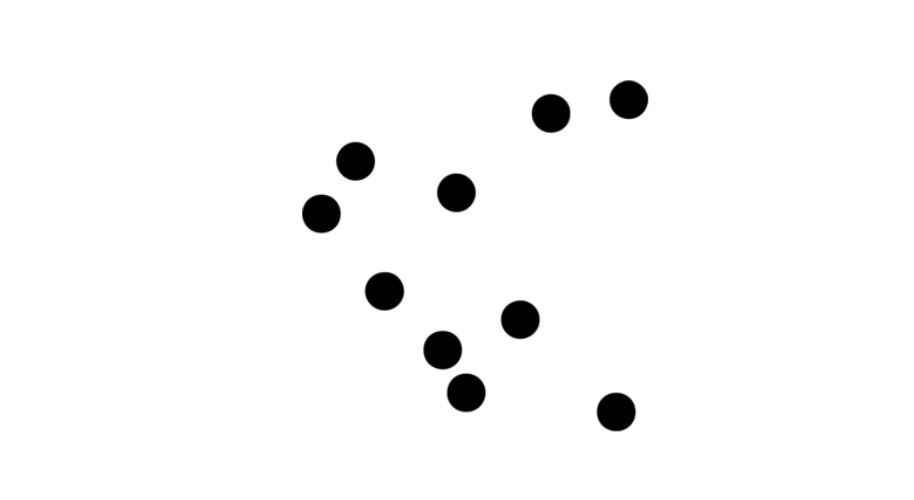 | As above. | As above. | As above. |
| Counting C | Count 7 dots | 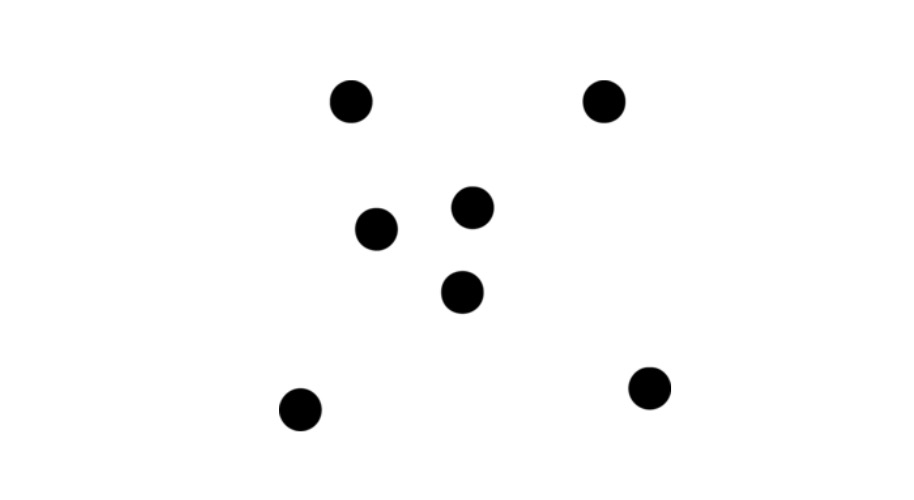 | As above. | As above. | As above. |
| Counting D | Count 9 dots. | 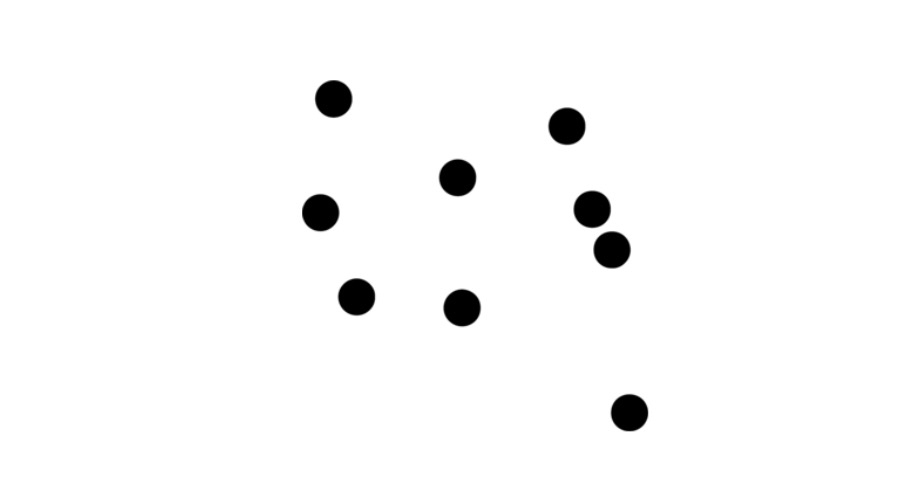 | As above. | As above. | As above. |

Table S18. **Question 16 of the ACoE.** Language function, subdomain simultanagnosia, tested by natural language processing.

| Name | Task | Example | Preprocessing | Processing | Scoring |
| --- | --- | --- | --- | --- | --- |
| Counting A | Name shown letter | 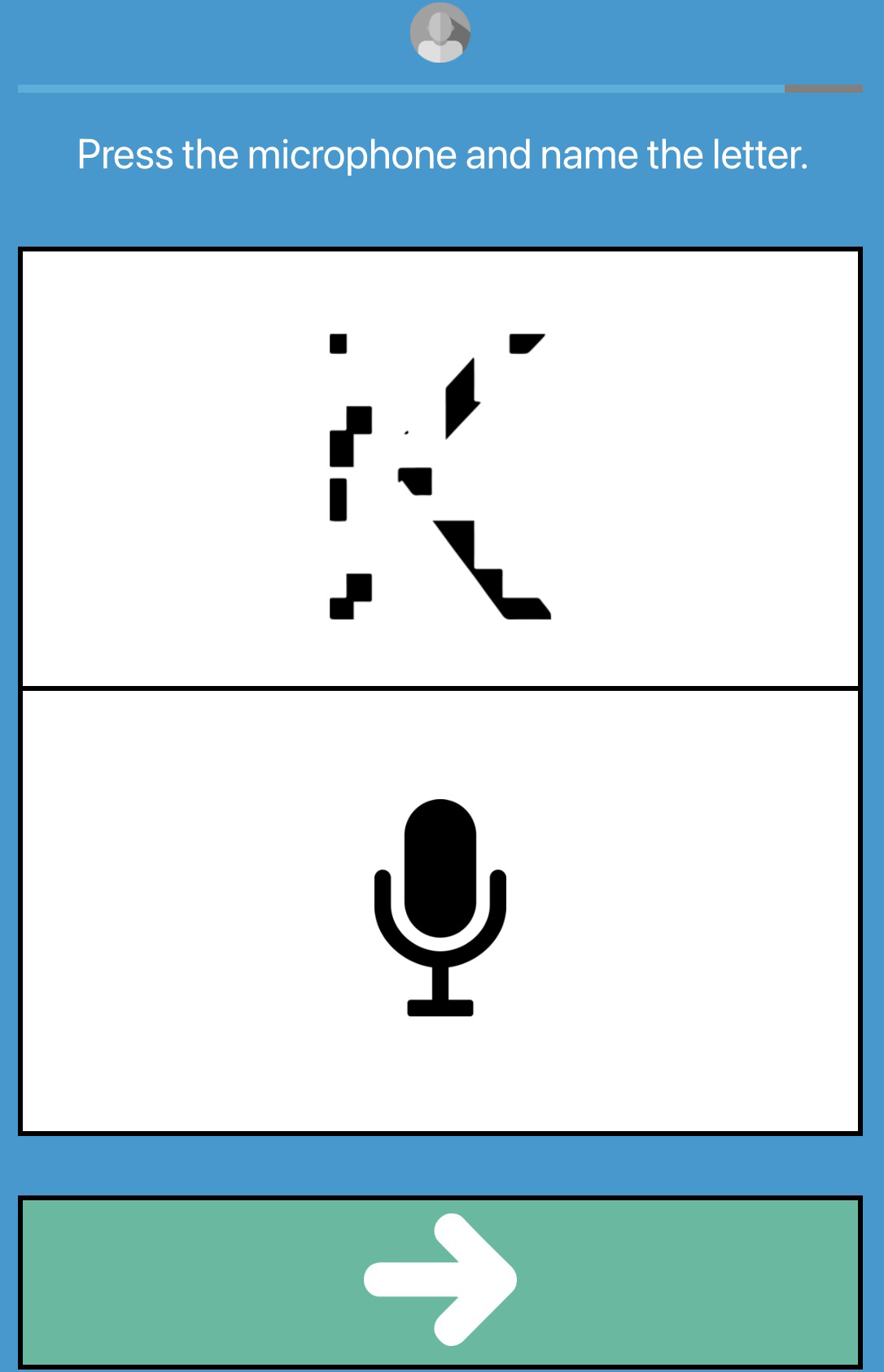 | MP4 uploaded.  FFMPEG for preprocessing.  1s pre and post.  Normalized to background noise.  Bitrate normalized.  Upsampled. | Passed through NLP Speech Recognition  Python Package.  Compare detected letter to normative list of acceptable words. | 0-1 Points  Point if correct letter. |
| Counting B | Name shown letter | 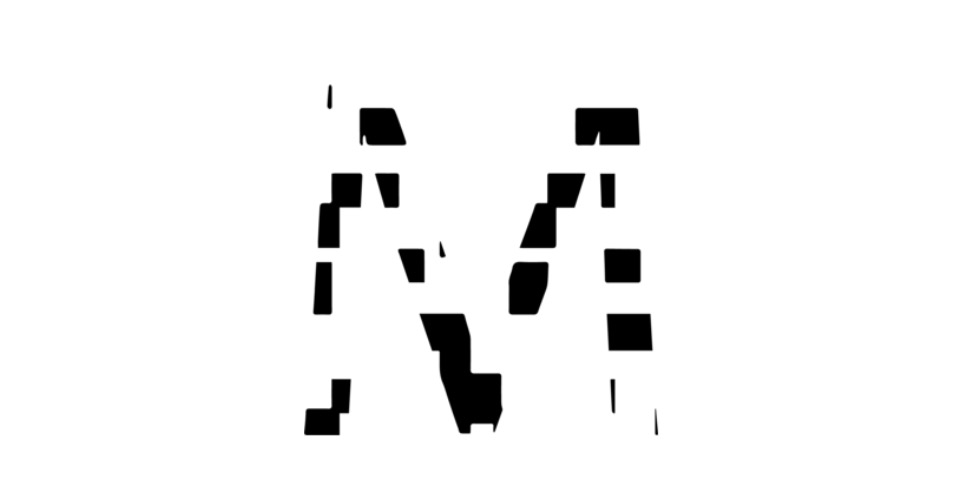 | As above. | As above. | As above. |
| Counting C | Name shown letter | 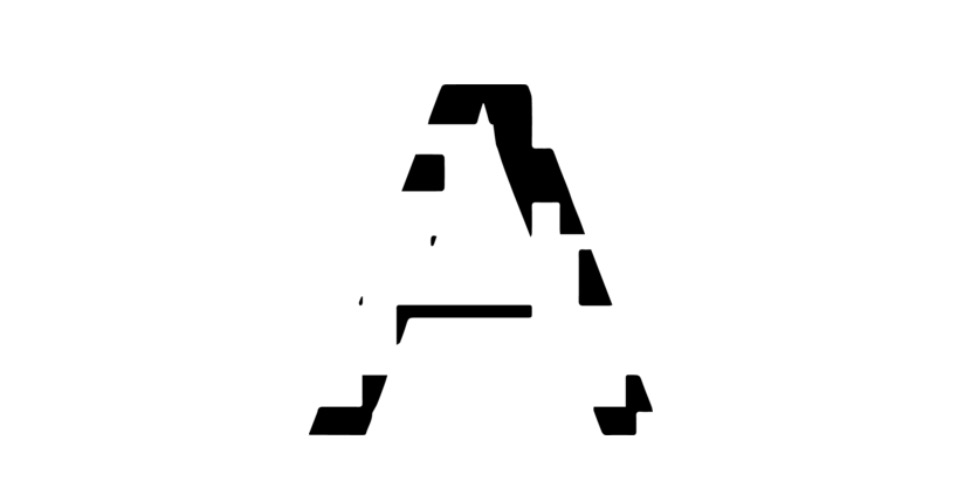 | As above. | As above. | As above. |
| Counting D | Name shown letter | 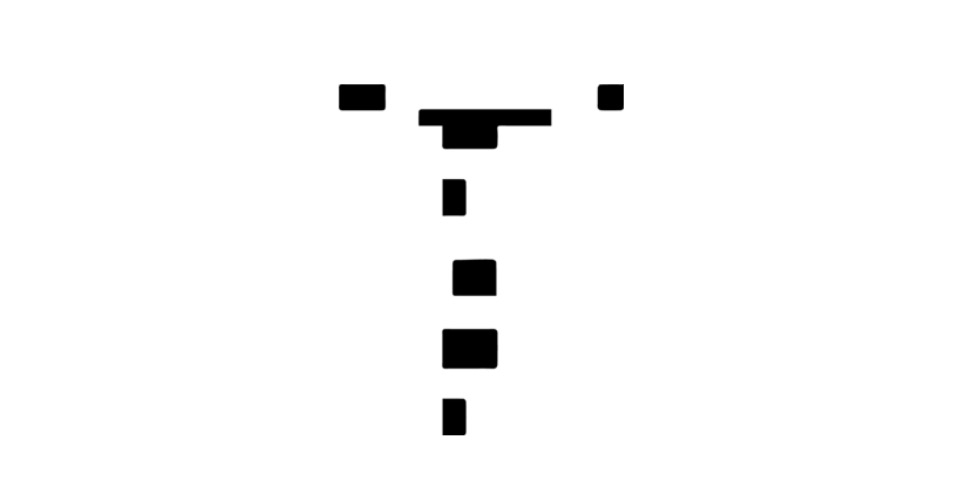 | As above. | As above. | As above. |

Table S19. **Question 17 of the ACoE.** Executive function, subdomain attention and temporal orientation, tested by expert algorithm.

| Name | Task | Example | Preprocessing | Processing | Scoring |
| --- | --- | --- | --- | --- | --- |
| Space A | Select day of the week from dropdown menu | 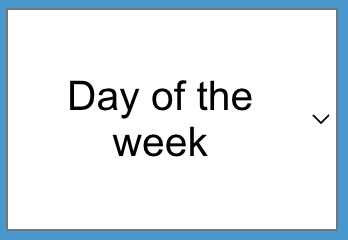 | Nil. | Access server clock.  Check if day within +/-1 days of current day of week. | 0-1 Points |
| Space B | Select month from dropdown menu | 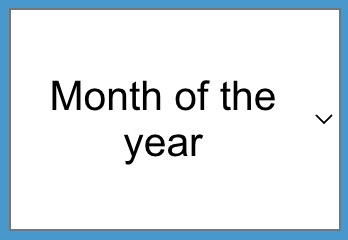 | Nil. | Access server clock.  Check if same month. | 0-1 Points |
| Space C | Select year from dropdown menu | 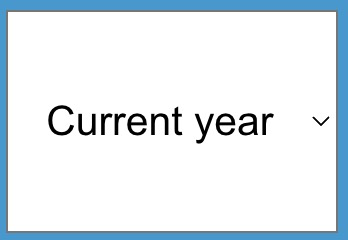 | Nil. | Access server clock.  Check if same year. | 0-1 Points |
| Space D | Select date of month from dropdown | 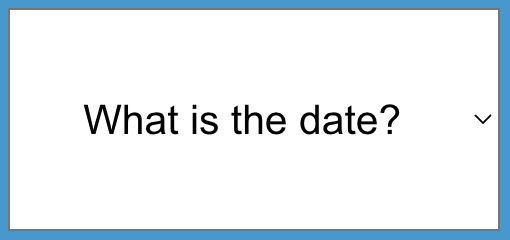 | Nil. | Access server clock.  Check if date within +/-1 of entered date. | 0-1 Points |
| Space E | Select season from dropdown | 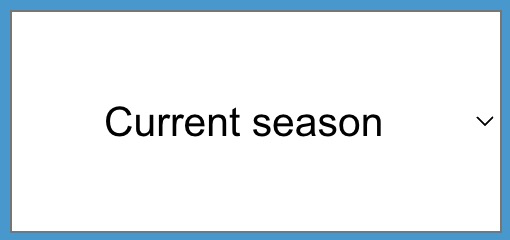 | Nil. | Access server clock.  Check if season correct. | 0-1 Points |

Table S20. **Question 18 of the ACoE.** Executive function, subdomain attention and spatial orientation, tested by expert algorithm.

| Name | Task | Example | Preprocessing | Processing | Scoring |
| --- | --- | --- | --- | --- | --- |
| Space A | Select answer from dropdown | 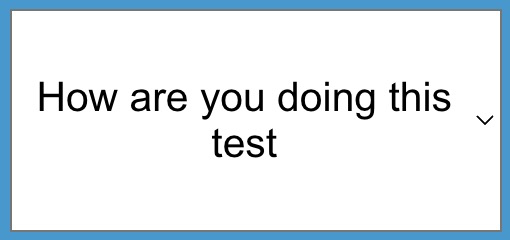 | Access device OS. | Compare answer to device operating system. | 0-1 Points |
| Space B | Type answer. | 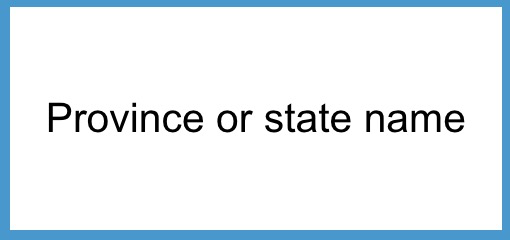 | Access device IP. | Triangulate device latitude and longitude from IP address.  Detect if IP within 100km of answer | 0-1 Points |
| Space C | Type answer | 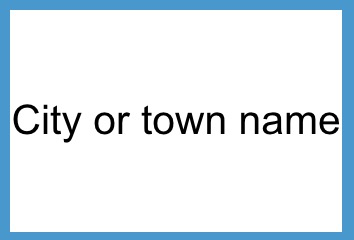 | Nil. | Same as above. | 0-1 Points |
| Space D | Select answer from dropdown | 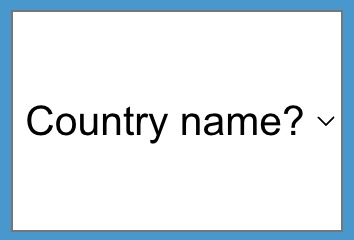 | Nil. | Same as above. | 0-1 Points |
| Space E | Select from dropdown. | 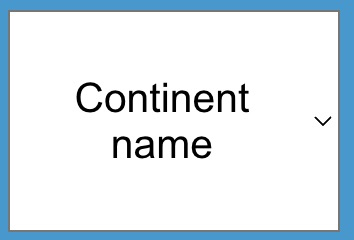 | Nil. | Same as above. | 0-1 Points |

Table S21. **Question 19 of the ACoE.** Memory function, tested by natural language processing

| Name | Task | Example | Preprocessing | Processing | Scoring |
| --- | --- | --- | --- | --- | --- |
| Delayed recall | Press microphone  State 7-part foreign address.  2 entrains.  1 test. | 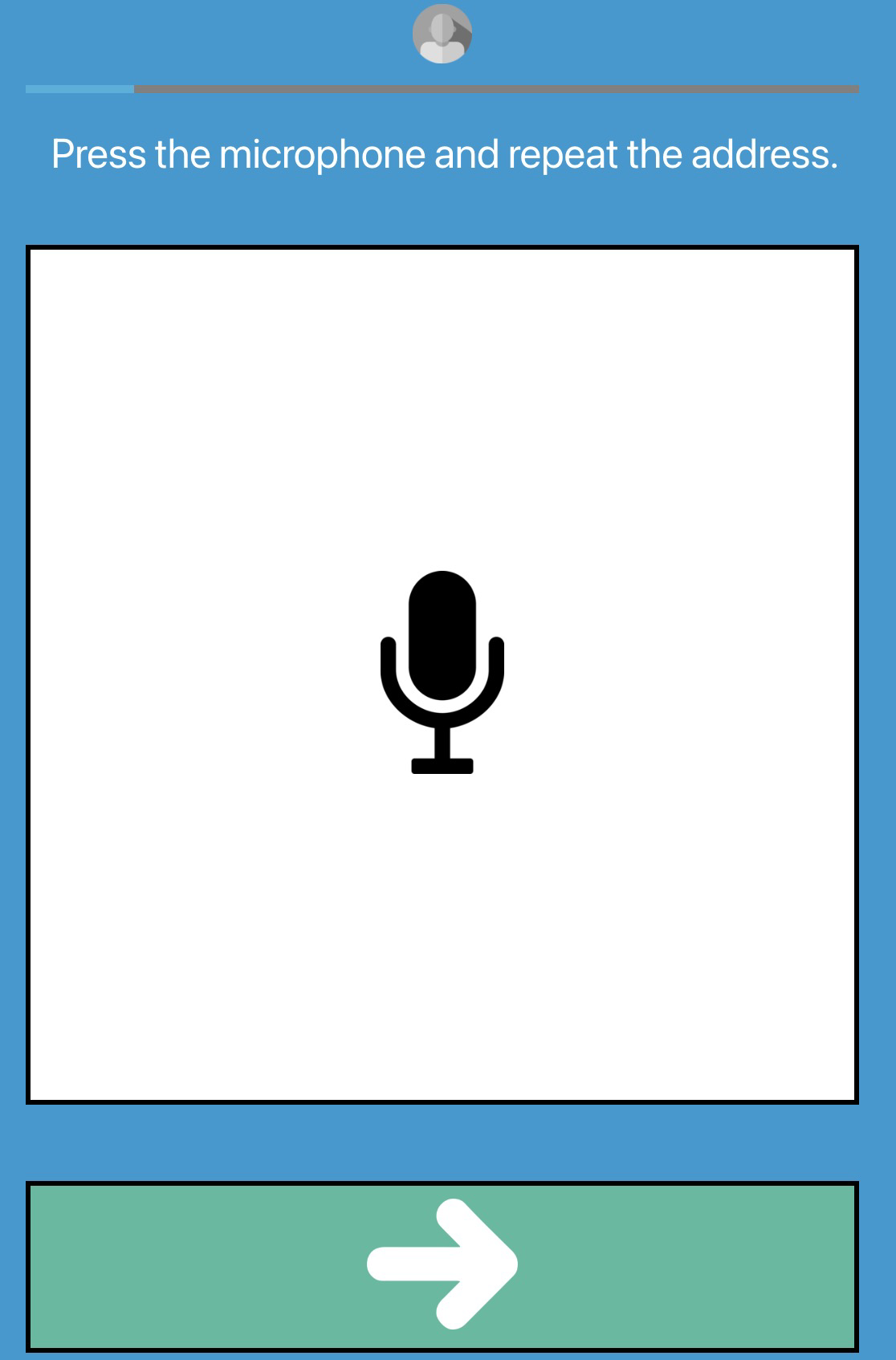 | MP4 uploaded.  FFMPEG for preprocessing.  1s pre and post.  Normalized to background noise.  Bitrate normalized.  Upsampled. | Passed through NLP Speech Recognition  Python Package.  Compare detected words to normative list of acceptable words. | 0-7 points.  1 point per part |

$$ACoE Score=80+19\left( Cognitive Status \right)-0.22(Age-30)$$

Equation S1. **The relationship of cognitive status on age upon ACoE scores, identified by multivariate regression.**

$$Age Adjusted ACoE Score=ACoE Score+0.22(Age-30)$$

Equation S2. **The formula to adjust ACoE score for patient age, based on Equation S1.**

Figure S1. **CONSORT Flow Diagram of Crossover Trial.** 132 patients were evaluated for enrollment. 86 patients were excluded. 46 patients were enrolled in the trial, with 24 randomized to receive the ACoE first and 22 randomized to receive the ACE-3 first. Subsequently, each arm then crossed over and received the other test. 11 total patients were lost to follow-up. 35 patients completed the study and were analyzed.

 Figure S2. **Distributions of scores across randomization arms.** A) Central tendencies of ACoE scores across randomization arms. Wilcoxon-Mann-Whitney U-Test demonstrates no significant difference (Group 1: 82, IQR 72.5-92; Group 2: 85.5, IQR 75-95.3, p = 0.82). B) Central tendencies of ACE-3 scores across randomization arms. Wilcoxon-Mann-Whitney U-Test demonstrates no significant difference (Group 1: 86, IQR (77-94.5); Group 2: 91.5, IQR 83.8-96, p = 0.65).


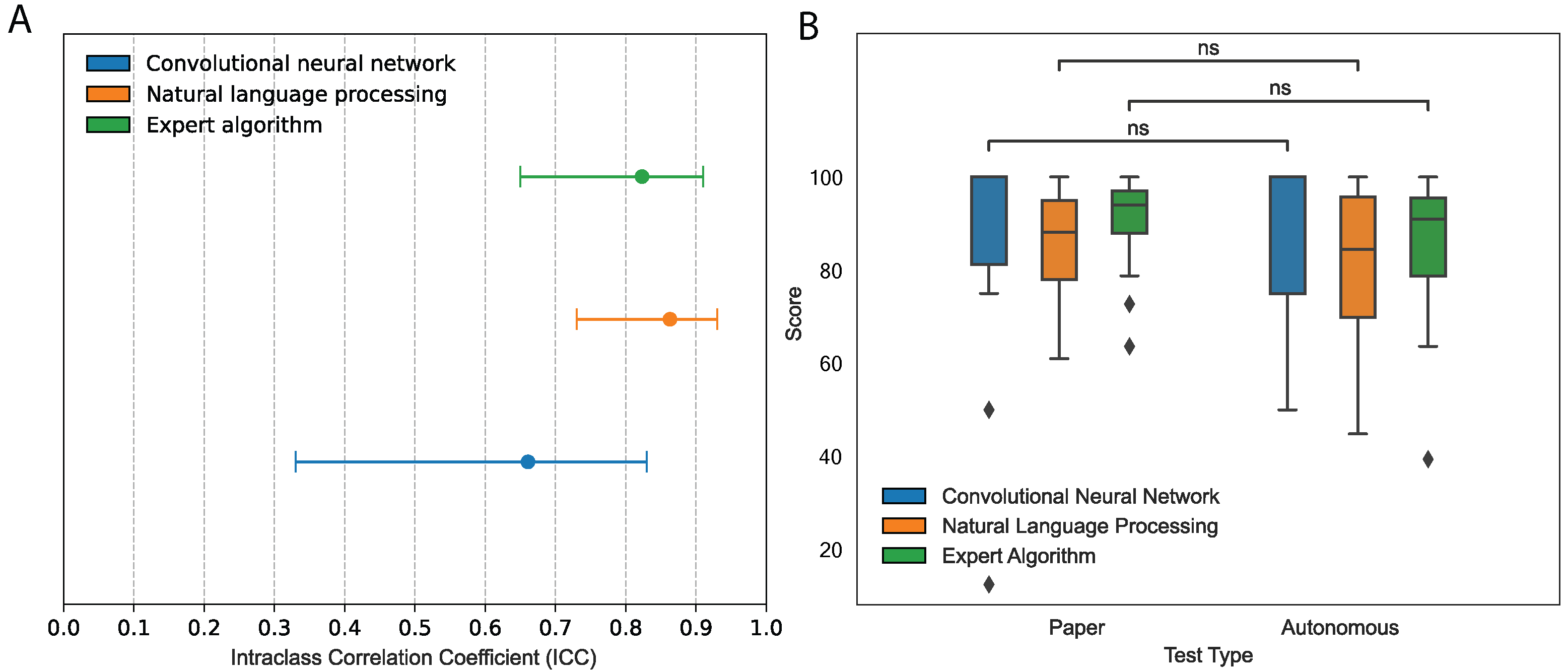


Figure S3. **Reliability of the algorithms used to assess patient inputs.** A) Reliability of the algorithms used in the ACoE range from high to very high as measured by ICC. 95% confidence intervals are presented with each ICC. The three primary algorithms were reliable, including computer vision (ICC = 0.67, p_FWE_ < .001), natural language processing (ICC = 0.86, p_FWE_ < .001), and the expert algorithms (ICC = 0.82, p_FWE_ < .001). B) Wilcoxon test demonstrates no significant difference between central tendencies of any test (p_min_ = .37). Scores are presented as percent.

**
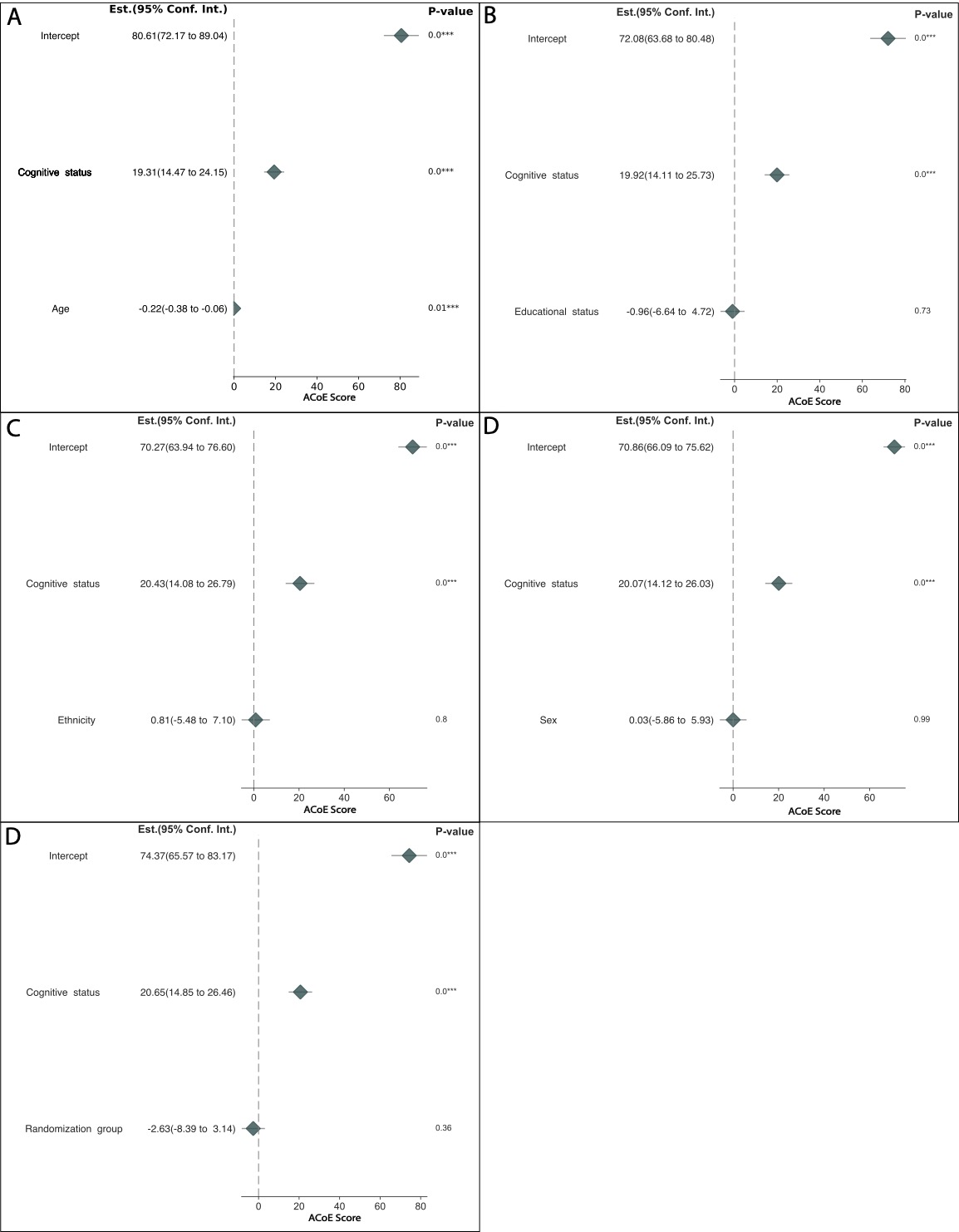
** Figure S4. **Only Age is Associated with ACoE Score After Controlling for Cognitive Status.** A) Multivariate regression of age and cognitive status upon ACoE score. B) Multivariate regression of educational status and cognitive status upon ACoE score. C) Multivariate regression of ethnicity and cognitive status upon ACoE score. D) Multivariate regression of sex and cognitive status upon ACoE score. E) Multivariate regression of randomization group and cognitive status upon ACoE score. Each multivariate regression is presented as a forest plot, with coefficients reported as estimates with 95% confidence intervals. The coefficients and confidence intervals are forest plotted. P-values for each coefficient are shown at the right. Unstandardized coefficients are presented to ensure directly interpretability and application in relation to ACoE scores.


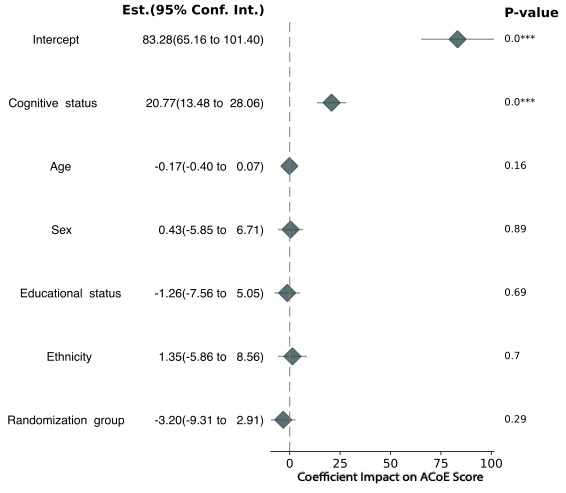


Figure S5. **Only Cognitive Status is Associated with ACE-3 Score.** Multivariate regression of all covariates on ACE-3 scores. The coefficients and confidence intervals are forest plotted and presented as their estimates with 95% confidence intervals. P-values for each coefficient are shown at the right. Unstandardized coefficients are presented to ensure directly interpretability and application in relation to ACoE scores.


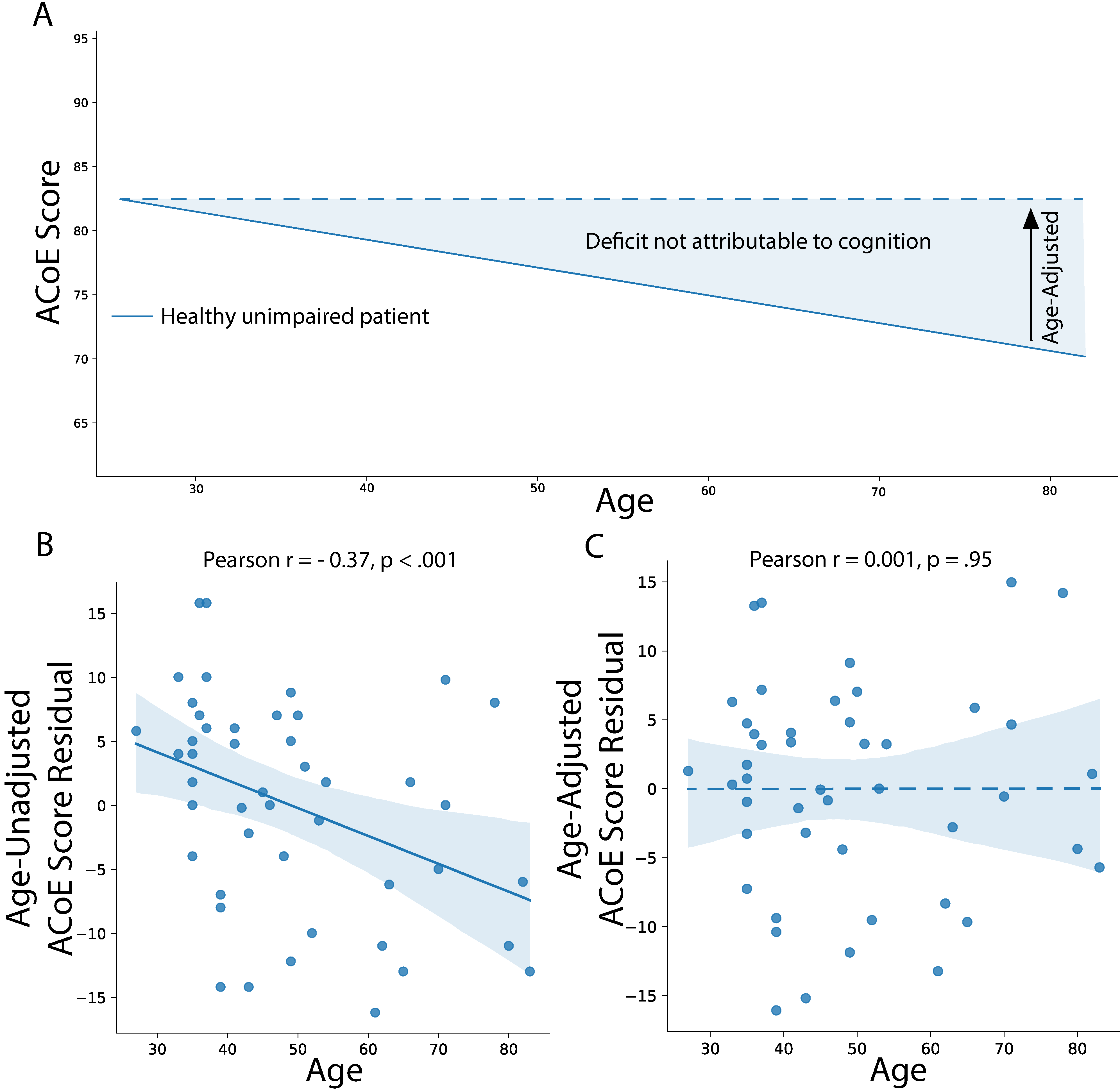


Figure S6. **Adjustment of ACoE Scores for the Effect of Age.** A) A diagrammatic representation of the relationship between age and ACoE Scores. Even in cognitively intact patients, older age steadily reduced patient scores. B) Correlation of age to ACoE scores after regressing cognitive status out. There is a significant relationship between age and the residual ACoE scores, which is completely unrelated to cognition (Pearson r = -0.37, p < .001). C) Successful adjustment of ACoE scores for the effect of age. Correlation of age to the age-adjusted ACoE scores with cognitive status regressed out. After correcting for age, the relationship between age and residual ACoE scores (Pearson r = 0.001, p = .95).


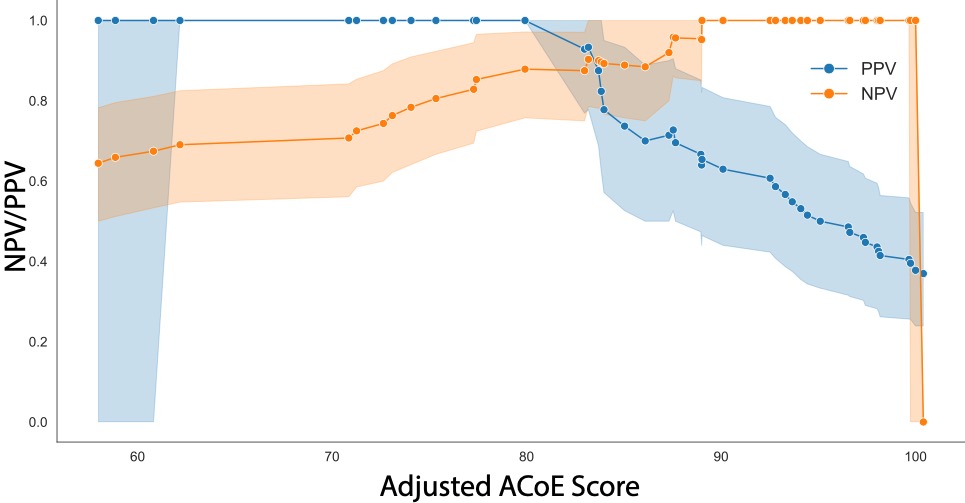


Figure S7. **Score-specific positive and negative predictive values are consistent with the sensitivity and specificity of the ACoE.** Below a score of 83% corresponds to a positive predictive value of 0.93 (95CI 0.79-1.0). Above a score of 89% corresponds to a negative predictive value of 0.95 (95%CI 0.78-1.0). Negative and positive predictive values become unstable and meaningless at the extremes of data ranges, resulting in confidence intervals destabilizing and spanning 0.0-1.0.


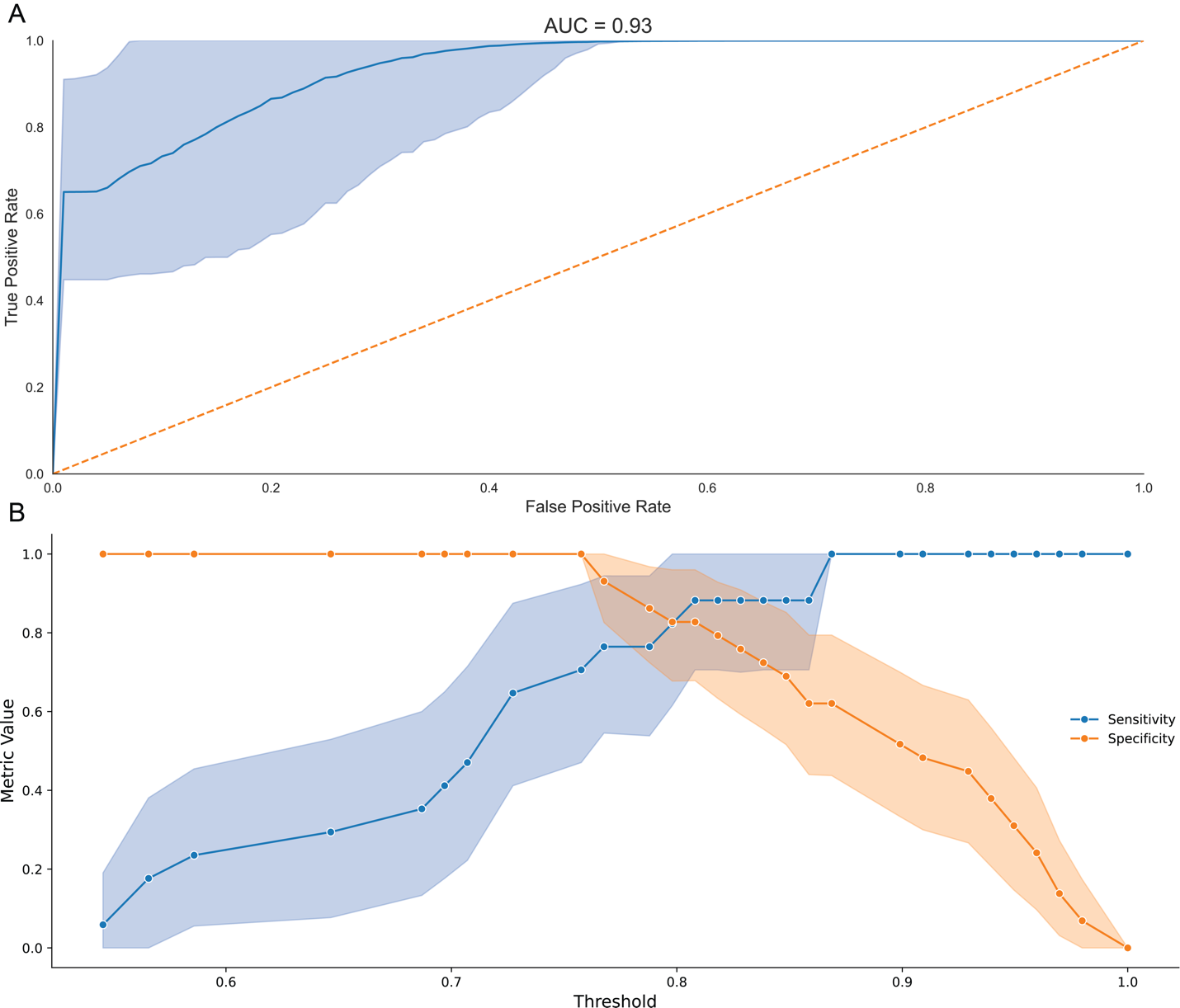


Figure S8. **Unadjusted ACoE Score Maintains Diagnostic Accuracy of Paper-Based Tests.** A) ROC and AUC of the unadjusted ACoE. AUC of diagnosis is 0.93 compared to ACE-3 and MoCA evaluation of patients. B) Sensitivity and specificity curves across ACoE scores. Under an unadjusted score of 76%, the ACoE achieves a specificity of 1.0 (95%CI 1.0-1.0). Above an unadjusted score of 86%, the ACoE achieves a sensitivity of 1.0 (95%CI 1.0-1.0).

Table S22. **Cognitive Domain Central Tendencies Between ACoE and ACE-3.** Values are reported as mean +/- standard error of the mean (median).

| Cognitive Domain | ACoE | ACE-3 | p-value |
| --- | --- | --- | --- |
| Attention | 16.11 +/- 0.32 (16.0) | 16.89 +/- 0.28 (18.0) | .84 |
| Memory | 18.35 +/- 0.91 (19.0) | 20.6 +/- 0.77 (22.0) | .73 |
| Fluency | 10.24 +/- 0.54 (11.0) | 11.23 +/- 0.43 (12.0) | .47 |
| Language | 22.91 +/- 0.5 (24.0) | 23.77 +/- 0.5 (25.0) | .78 |
| Visuospatial | 14.41 +/- 0.25 (14.0) | 14.63 +/- 0.36 (15.0) | .91 |

Table S23. **Algorithm Scoring Central Tendencies Between ACoE and ACE-3.** Values are reported as mean +/- standard error of the mean (median).

| Column Name | ACoE | ACE-3 | p-value |
| --- | --- | --- | --- |
| Convolutional Neural Network | 6.89 +/- 0.16 (6.5) | 7.03 +/- 0.26 (8.0) | .91 |
| Natural Language Processing | 46.89 +/- 1.27 (47.0) | 50.26 +/- 1.09 (52.0) | .68 |
| Expert Algorithm | 28.24 +/- 0.64 (30.0) | 29.83 +/- 0.48 (31.0) | .73 |

Table S24. **Question Scoring Central Tendencies Between ACoE and ACE-3.** Values are reported as mean +/- standard error of the mean (median). P-values are reported using tests of the median.

| Question | ACoE | ACE-3 | p-value |
| --- | --- | --- | --- |
| 1 | 9.63 +/- 0.11 (10.0) | 9.69 +/- 0.15 (10.0) | .63 |
| 2 | 2.84 +/- 0.13 (3.0) | 2.97 +/- 0.03 (3.0) | .73 |
| 3 | 3.93 +/- 0.24 (5.0) | 4.23 +/- 0.21 (5.0) | .41 |
| 4 | 2.35 +/- 0.15 (3.0) | 2.71 +/- 0.11 (3.0) | .57 |
| 5 | 10.24 +/- 0.54 (11.0) | 11.23 +/- 0.43 (12.0) | .40 |
| 6 | 5.09 +/- 0.33 (6.0) | 5.97 +/- 0.22 (7.0) | .29 |
| 7 | 2.72 +/- 0.15 (3.0) | 3.03 +/- 0.15 (3.0) | .78 |
| 8 | 2.43 +/- 0.11 (3.0) | 2.89 +/- 0.05 (3.0) | .74 |
| 9 | 1.59 +/- 0.1 (2.0) | 1.86 +/- 0.06 (2.0) | .62 |
| 10 | 1.78 +/- 0.07 (2.0) | 1.8 +/- 0.07 (2.0) | .77 |
| 11 | 1.83 +/- 0.06 (2.0) | 1.89 +/- 0.05 (2.0) | .81 |
| 12 | 11.0 +/- 0.32 (12.0) | 11.14 +/- 0.3 (12.0) | .71 |
| 13 | 3.74 +/- 0.09 (4.0) | 3.6 +/- 0.12 (4.0) | .73 |
| 14 | 0.54 +/- 0.07 (1.0) | 0.6 +/- 0.08 (1.0) | .78 |
| 15 | 6.89 +/- 0.16 (6.5) | 7.03 +/- 0.26 (8.0) | .49 |
| 16 | 3.76 +/- 0.09 (4.0) | 3.8 +/- 0.1 (4.0) | .75 |
| 17 | 3.76 +/- 0.1 (4.0) | 3.8 +/- 0.12 (4.0) | .78 |
| 18 | 4.0 +/- 0.36 (4.0) | 4.34 +/- 0.44 (5.0) | .55 |
| 19 | 4.2 +/- 0.18 (5.0) | 4.54 +/- 0.13 (5.0) | .70 |


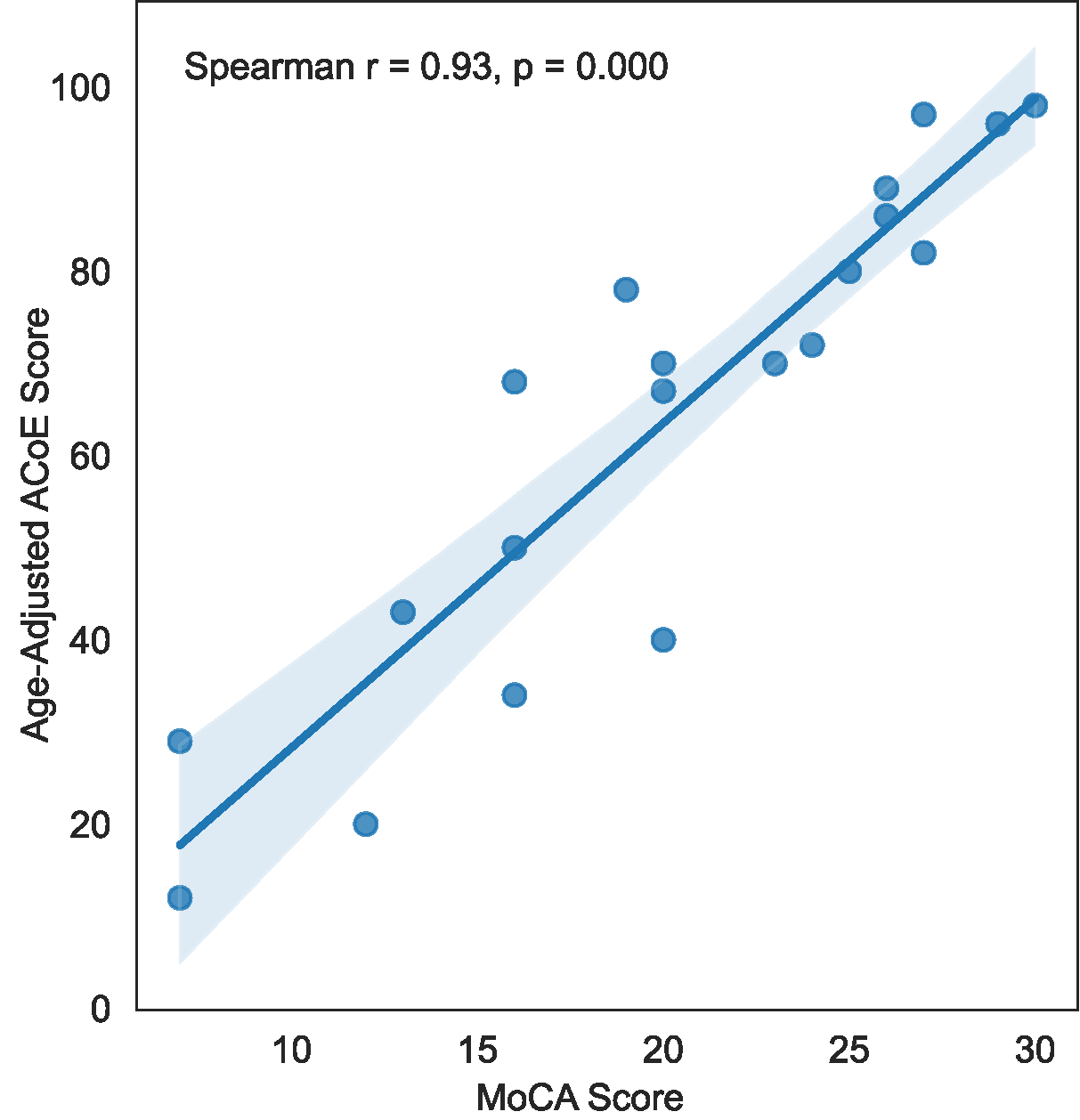


Figure S9. **The ACoE maintains reliable cognitive assessment in patients over the age of 65.** Age-adjusted ACoE scores from 20 patients over the age of 65 were correlated to their corresponding MoCA scores (average age = 70 ± 3.2 years).
