# Supplementary material for: Machine Learning–Based Cognitive Assessment With The Autonomous Cognitive Examination: Randomized Controlled Trial
Source: J Med Internet Res. 2025 Jul 30;27:e67446. doi: 10.2196/67446 (PMC12310151; doi:10.2196/67446)
Supplement: Checklist 1 [file jmir-v27-e67446-s002.pdf]

CONSORT-EHEALTH (V 1.6.1) - Submission/Publication Form

The CONSORT-EHEALTH checklist is intended for authors of randomized trials evaluating web-based and Internet-based applications/interventions, including mobile interventions, electronic games (incl multiplayer games), social media, certain telehealth applications, and other interactive and/or networked electronic applications. Some of the items (e.g. all subitems under item 5 - description of the intervention) may also be applicable for other study designs.

The goal of the CONSORT EHEALTH checklist and guideline is to be
a) a guide for reporting for authors of RCTs,
b) to form a basis for appraisal of an ehealth trial (in terms of validity)

CONSORT-EHEALTH items/subitems are MANDATORY reporting items for studies published in the Journal of Medical Internet Research and other journals / scientific societies endorsing the checklist.

Items numbered 1., 2., 3., 4a., 4b etc are original CONSORT or CONSORT-NPT (non-pharmacologic treatment) items.
Items with Roman numerals (i., ii, iii, iv etc.) are CONSORT-EHEALTH extensions/clarifications.

As the CONSORT-EHEALTH checklist is still considered in a formative stage, we would ask that you also RATE ON A SCALE OF 1-5 how important/useful you feel each item is FOR THE PURPOSE OF THE CHECKLIST and reporting guideline (optional).

Mandatory reporting items are marked with a red *.
In the textboxes, either copy & paste the relevant sections from your manuscript into this form - please include any quotes from your manuscript in QUOTATION MARKS,
or answer directly by providing additional information not in the manuscript, or elaborating on why the item was not relevant for this study.

YOUR ANSWERS WILL BE PUBLISHED AS A SUPPLEMENTARY FILE TO YOUR PUBLICATION IN JMIR AND ARE CONSIDERED PART OF YOUR PUBLICATION (IF ACCEPTED).
Please fill in these questions diligently. Information will not be copyedited, so please use proper spelling and grammar, use correct capitalization, and avoid abbreviations.

DO NOT FORGET TO SAVE AS PDF _AND_ CLICK THE SUBMIT BUTTON SO YOUR ANSWERS ARE IN OUR DATABASE !!!

Citation Suggestion (if you append the pdf as Appendix we suggest to cite this paper in the caption):
Eysenbach G, CONSORT-EHEALTH Group

CONSORT-EHEALTH: Improving and Standardizing Evaluation Reports of Web-based and Mobile Health Interventions
J Med Internet Res 2011;13(4):e126
URL: http://www.jmir.org/2011/4/e126/
doi: 10.2196/jmir.1923
PMID: 22209829

calvinwhow@gmail.com Switch account

Not shared

Draft saved

* Indicates required question

Your name *

First Last

Calvin Howard

Primary Affiliation (short), City, Country *

University of Toronto, Toronto, Canada

Harvard Medical School, Boston, USA

Your e-mail address *

abc@gmail.com

choward12@bwh.harvard.edu

Title of your manuscript *

Provide the (draft) title of your manuscript.

Randomized Controlled Trial of Machine- Learning –Based Cognitive AssessmenttMachine Learning–Based Cognitive Assessment With the Autonomous Cognitive Examination: Randomized Controlled Trial

Name of your App/Software/Intervention *

If there is a short and a long/alternate name, write the short name first and add the long name in brackets.

Autonomous Cognitive Examination

Evaluated Version (if any)

e.g. "V1", "Release 2017-03-01", "Version 2.0.27913"

V1

Language(s) *

What language is the intervention/app in? If multiple languages are available, separate by comma (e.g. "English, French")

English

URL of your Intervention Website or App

e.g. a direct link to the mobile app on app in appstore (itunes, Google Play), or URL of the website. If the intervention is a DVD or hardware, you can also link to an Amazon page.

demo.cog-net.com

URL of an image/screenshot (optional)

Your answer

Accessibility *

Can an enduser access the intervention presently?

- (selected) access is free and open
- access only for special usergroups, not open
- access is open to everyone, but requires payment/subscription/in-app purchases
- app/intervention no longer accessible
- Other:

Primary Medical Indication/Disease/Condition *

e.g. "Stress", "Diabetes", or define the target group in brackets after the condition, e.g. "Autism (Parents of children with)", "Alzheimers (Informal Caregivers of)"

Neurocognitive disorder

Primary Outcomes measured in trial *

comma-separated list of primary outcomes reported in the trial

Cognitive dysfunction

Secondary/other outcomes

Are there any other outcomes the intervention is expected to affect?

Your answer

Recommended "Dose" *

What do the instructions for users say on how often the app should be used?

- ○ Approximately Daily
- ○ Approximately Weekly
- ○ Approximately Monthly
- ◉ Approximately Yearly
- ○ "as needed"
- ○ Other:

Approx. Percentage of Users (starters) still using the app as recommended after 3 months *

- (x) unknown / not evaluated
- ( ) 0-10%
- ( ) 11-20%
- ( ) 21-30%
- ( ) 31-40%
- ( ) 41-50%
- ( ) 51-60%
- ( ) 61-70%
- ( ) 71%-80%
- ( ) 81-90%
- ( ) 91-100%
- ( ) Other:

Overall, was the app/intervention effective? *

- (x) yes: all primary outcomes were significantly better in intervention group vs control
- ( ) partly: SOME primary outcomes were significantly better in intervention group vs control
- ( ) no statistically significant difference between control and intervention
- ( ) potentially harmful: control was significantly better than intervention in one or more outcomes
- ( ) inconclusive: more research is needed
- ( ) Other:

Article Preparation Status/Stage *

At which stage in your article preparation are you currently (at the time you fill in this form)

- ○ not submitted yet - in early draft status
- ○ not submitted yet - in late draft status, just before submission
- ○ submitted to a journal but not reviewed yet
- ○ submitted to a journal and after receiving initial reviewer comments
- ◉ submitted to a journal and accepted, but not published yet
- ○ published
- ○ Other:

Journal *

If you already know where you will submit this paper (or if it is already submitted), please provide the journal name (if it is not JMIR, provide the journal name under "other")

- ○ not submitted yet / unclear where I will submit this
- ◉ Journal of Medical Internet Research (JMIR)
- ○ JMIR mHealth and UHealth
- ○ JMIR Serious Games
- ○ JMIR Mental Health
- ○ JMIR Public Health
- ○ JMIR Formative Research
- ○ Other JMIR sister journal
- ○ Other:

Is this a full powered effectiveness trial or a pilot/feasibility trial? *

- ○ Pilot/feasibility
- ◉ Fully powered

Manuscript tracking number *

If this is a JMIR submission, please provide the manuscript tracking number under "other" (The ms tracking number can be found in the submission acknowledgement email, or when you login as author in JMIR. If the paper is already published in JMIR, then the ms tracking number is the four-digit number at the end of the DOI, to be found at the bottom of each published article in JMIR)

- ○ no ms number (yet) / not (yet) submitted to / published in JMIR
- ◉ Other: 67446

TITLE AND ABSTRACT

1a) TITLE: Identification as a randomized trial in the title

1a) Does your paper address CONSORT item 1a? *

I.e does the title contain the phrase "Randomized Controlled Trial"? (if not, explain the reason under "other")

- ◉ yes
- ○ Other:

1a-i) Identify the mode of delivery in the title

Identify the mode of delivery. Preferably use “web-based” and/or “mobile” and/or “electronic game” in the title. Avoid ambiguous terms like “online”, “virtual”, “interactive”. Use “Internet-based” only if Intervention includes non-web-based Internet components (e.g. email), use “computer-based” or “electronic” only if offline products are used. Use “virtual” only in the context of “virtual reality” (3-D worlds). Use “online” only in the context of “online support groups”. Complement or substitute product names with broader terms for the class of products (such as “mobile” or “smart phone” instead of “iphone”), especially if the application runs on different platforms.

	1	2	3	4	5	
subitem not at all important	○	○	○	○	●	essential

Clear selection

Does your paper address subitem 1a-i? *

Copy and paste relevant sections from manuscript title (include quotes in quotation marks "like this" to indicate direct quotes from your manuscript), or elaborate on this item by providing additional information not in the ms, or briefly explain why the item is not applicable/relevant for your study

Randomized Controlled Trial of Machine- Learning –Based Cognitive Assessment Machine Learning–Based Cognitive Assessment With the Autonomous Cognitive Examination: Randomized Controlled Trial

1a-ii) Non-web-based components or important co-interventions in title

Mention non-web-based components or important co-interventions in title, if any (e.g., “with telephone support”).

	1	2	3	4	5	
subitem not at all important	●	○	○	○	○	essential

Clear selection

Does your paper address subitem 1a-ii?

Copy and paste relevant sections from manuscript title (include quotes in quotation marks "like this" to indicate direct quotes from your manuscript), or elaborate on this item by providing additional information not in the ms, or briefly explain why the item is not applicable/relevant for your study

Your answer

1a-iii) Primary condition or target group in the title

Mention primary condition or target group in the title, if any (e.g., "for children with Type I Diabetes") Example: A Web-based and Mobile Intervention with Telephone Support for Children with Type I Diabetes: Randomized Controlled Trial

	1	2	3	4	5	
subitem not at all important	○	○	○	○	○	essential

Does your paper address subitem 1a-iii? *

Copy and paste relevant sections from manuscript title (include quotes in quotation marks "like this" to indicate direct quotes from your manuscript), or elaborate on this item by providing additional information not in the ms, or briefly explain why the item is not applicable/relevant for your study

Randomized Controlled Trial of Machine- Learning –Based Cognitive Assessment Machine Learning–Based Cognitive Assessment With the Autonomous Cognitive Examination: Randomized Controlled Trial

1b) ABSTRACT: Structured summary of trial design, methods, results, and conclusions

NPT extension: Description of experimental treatment, comparator, care providers, centers, and blinding status.

1b-i) Key features/functionalities/components of the intervention and comparator in the METHODS section of the ABSTRACT

Mention key features/functionalities/components of the intervention and comparator in the abstract. If possible, also mention theories and principles used for designing the site. Keep in mind the needs of systematic reviewers and indexers by including important synonyms. (Note: Only report in the abstract what the main paper is reporting. If this information is missing from the main body of text, consider adding it)

	1	2	3	4	5	
subitem not at all important	○	○	○	○	○	essential

Does your paper address subitem 1b-i? *

Copy and paste relevant sections from the manuscript abstract (include quotes in quotation marks "like this" to indicate direct quotes from your manuscript), or elaborate on this item by providing additional information not in the ms, or briefly explain why the item is not applicable/relevant for your study

Randomized Controlled Trial of Machine- Learning –Based Cognitive Assessment Machine Learning–Based Cognitive Assessment With the Autonomous Cognitive Examination: Randomized Controlled Trial

1b-ii) Level of human involvement in the METHODS section of the ABSTRACT

Clarify the level of human involvement in the abstract, e.g., use phrases like “fully automated” vs. “therapist/nurse/care provider/physician-assisted” (mention number and expertise of providers involved, if any). (Note: Only report in the abstract what the main paper is reporting. If this information is missing from the main body of text, consider adding it)

	1	2	3	4	5	
subitem not at all important	○	○	○	○	○	essential

Does your paper address subitem 1b-ii?

Copy and paste relevant sections from the manuscript abstract (include quotes in quotation marks "like this" to indicate direct quotes from your manuscript), or elaborate on this item by providing additional information not in the ms, or briefly explain why the item is not applicable/relevant for your study

Your answer

1b-iii) Open vs. closed, web-based (self-assessment) vs. face-to-face assessments in the METHODS section of the ABSTRACT

Mention how participants were recruited (online vs. offline), e.g., from an open access website or from a clinic or a closed online user group (closed usergroup trial), and clarify if this was a purely web-based trial, or there were face-to-face components (as part of the intervention or for assessment). Clearly say if outcomes were self-assessed through questionnaires (as common in web-based trials). Note: In traditional offline trials, an open trial (open-label trial) is a type of clinical trial in which both the researchers and participants know which treatment is being administered. To avoid confusion, use “blinded” or “unblinded” to indicated the level of blinding instead of “open”, as “open” in web-based trials usually refers to “open access” (i.e. participants can self-enrol). (Note: Only report in the abstract what the main paper is reporting. If this information is missing from the main body of text, consider adding it)

	1	2	3	4	5	
subitem not at all important	○	○	○	○	○	essential

Does your paper address subitem 1b-iii?

Copy and paste relevant sections from the manuscript abstract (include quotes in quotation marks "like this" to indicate direct quotes from your manuscript), or elaborate on this item by providing additional information not in the ms, or briefly explain why the item is not applicable/relevant for your study

Your answer

1b-iv) RESULTS section in abstract must contain use data

Report number of participants enrolled/assessed in each group, the use/uptake of the intervention (e.g., attrition/adherence metrics, use over time, number of logins etc.), in addition to primary/secondary outcomes. (Note: Only report in the abstract what the main paper is reporting. If this information is missing from the main body of text, consider adding it)

	1	2	3	4	5	
subitem not at all important	○	○	○	○	○	essential

Does your paper address subitem 1b-iv?

Copy and paste relevant sections from the manuscript abstract (include quotes in quotation marks "like this" to indicate direct quotes from your manuscript), or elaborate on this item by providing additional information not in the ms, or briefly explain why the item is not applicable/relevant for your study

Your answer

1b-v) CONCLUSIONS/DISCUSSION in abstract for negative trials

Conclusions/Discussions in abstract for negative trials: Discuss the primary outcome - if the trial is negative (primary outcome not changed), and the intervention was not used, discuss whether negative results are attributable to lack of uptake and discuss reasons. (Note: Only report in the abstract what the main paper is reporting. If this information is missing from the main body of text, consider adding it)

	1	2	3	4	5	
subitem not at all important	○	○	○	○	○	essential

Does your paper address subitem 1b-v?

Copy and paste relevant sections from the manuscript abstract (include quotes in quotation marks "like this" to indicate direct quotes from your manuscript), or elaborate on this item by providing additional information not in the ms, or briefly explain why the item is not applicable/relevant for your study

Your answer

INTRODUCTION

2a) In INTRODUCTION: Scientific background and explanation of rationale

2a-i) Problem and the type of system/solution

Describe the problem and the type of system/solution that is object of the study: intended as stand-alone intervention vs. incorporated in broader health care program? Intended for a particular patient population? Goals of the intervention, e.g., being more cost-effective to other interventions, replace or complement other solutions? (Note: Details about the intervention are provided in "Methods" under 5)

	1	2	3	4	5	
subitem not at all important	○	○	○	○	○	essential

Does your paper address subitem 2a-i? *

Copy and paste relevant sections from the manuscript (include quotes in quotation marks "like this" to indicate direct quotes from your manuscript), or elaborate on this item by providing additional information not in the ms, or briefly explain why the item is not applicable/relevant for your study

Your answer

2a-ii) Scientific background, rationale: What is known about the (type of) system

Scientific background, rationale: What is known about the (type of) system that is the object of the study (be sure to discuss the use of similar systems for other conditions/diagnoses, if appropiate), motivation for the study, i.e. what are the reasons for and what is the context for this specific study, from which stakeholder viewpoint is the study performed, potential impact of findings [2]. Briefly justify the choice of the comparator.

	1	2	3	4	5	
subitem not at all important	○	○	○	○	○	essential

Does your paper address subitem 2a-ii? *

Copy and paste relevant sections from the manuscript (include quotes in quotation marks "like this" to indicate direct quotes from your manuscript), or elaborate on this item by providing additional information not in the ms, or briefly explain why the item is not applicable/relevant for your study

Current projections estimate 150 million patients living with dementia worldwide by 2050, with 57 million as of 2019 [1] The aging population presents a considerable diagnostic challenge. This challenge has resulted in diagnostic timelines requiring 3-years or longer from symptom onset [2–6] with a large portion of dementia patients remaining undiagnosed [2,3,7,8]
Digital cognitive assessments (DCAs) offer a potential method to address the diagnostic challenge and help improve diagnostic timelines [9–11] Among other things, two factors that influence the utility of DCAs at a population level are accessibility and generalizability [11,12]
To increase accuracy, some DCAs may sacrifice a degree of accessibility to better control testing conditions [13,14] Often this means DCAs achieve their exceptional performance by requiring specific hardware [15–21] in-office expert administrators [13,15,16,18–20,22–24] or by limiting variability by using mouse-and-keyboard questionnaires [15,25–28] Eliminating these requirements could improve accessibility, for non-affluent, rural, or cognitively impaired patients.
Other DCAs may sacrifice some generalizability to focus on a specific disease. These DCAs often use disease-specific exam maneuvers [15,18,22,23,29–31] or disease-specific algorithms [32–37] with both approaches meant to maximize detection of specific diseases. By taking a step backward from highly focused assessments, providing a thorough cognitive examination may improve generalization.
We previously developed Autonomous Cognitive Examination (ACoE) to improve accessibility and generalizability. The ACoE uses various machine learning algorithms to provide a thorough assessment of cognition in a naturalistic and remote assessment [38,39] However, it has not been clinically validated and its utility is unknown.
Here, we evaluate the validity of the ACoE. We compare the ACoE against a comprehensive test, the Addenbrooke's Cognitive Examination-3 (ACE-3) [40,41] and a ubiquitously used test, the Montreal Cognitive Assessment (MoCA) [42] First, we compare the reliability of the ACoE to phenotype overall cognition and cognitive symptoms compared the ACE-3. We then evaluate the ability of the ACoE's phenotypic output to achieve similar screening results as the ACE-3 and MoCA.

2b) In INTRODUCTION: Specific objectives or hypotheses

Does your paper address CONSORT subitem 2b? *

Copy and paste relevant sections from the manuscript (include quotes in quotation marks "like this" to indicate direct quotes from your manuscript), or elaborate on this item by providing additional information not in the ms, or briefly explain why the item is not applicable/relevant for your study

Current projections estimate 150 million patients living with dementia worldwide by 2050, with 57 million as of 2019 [1] The aging population presents a considerable diagnostic challenge. This challenge has resulted in diagnostic timelines requiring 3-years or longer from symptom onset [2–6] with a large portion of dementia patients remaining undiagnosed [2,3,7,8]
Digital cognitive assessments (DCAs) offer a potential method to address the diagnostic challenge and help improve diagnostic timelines [9–11] Among other things, two factors that influence the utility of DCAs at a population level are accessibility and generalizability [11,12]
To increase accuracy, some DCAs may sacrifice a degree of accessibility to better control testing conditions [13,14] Often this means DCAs achieve their exceptional performance by requiring specific hardware [15–21] in-office expert administrators [13,15,16,18–20,22–24] or by limiting variability by using mouse-and-keyboard questionnaires [15,25–28] Eliminating these requirements could improve accessibility, for non-affluent, rural, or cognitively impaired patients.
Other DCAs may sacrifice some generalizability to focus on a specific disease. These DCAs often use disease-specific exam maneuvers [15,18,22,23,29–31] or disease-specific algorithms [32–37] with both approaches meant to maximize detection of specific diseases. By taking a step backward from highly focused assessments, providing a thorough cognitive examination may improve generalization.
We previously developed Autonomous Cognitive Examination (ACoE) to improve accessibility and generalizability. The ACoE uses various machine learning algorithms to provide a thorough assessment of cognition in a naturalistic and remote assessment [38,39] However, it has not been clinically validated and its utility is unknown.
Here, we evaluate the validity of the ACoE. We compare the ACoE against a comprehensive test, the Addenbrooke's Cognitive Examination-3 (ACE-3) [40,41] and a ubiquitously used test, the Montreal Cognitive Assessment (MoCA) [42] First, we compare the reliability of the ACoE to phenotype overall cognition and cognitive symptoms compared the ACE-3. We then evaluate the ability of the ACoE's phenotypic output to achieve similar screening results as the ACE-3 and MoCA.

METHODS

3a) Description of trial design (such as parallel, factorial) including allocation ratio

Does your paper address CONSORT subitem 3a? *

Copy and paste relevant sections from the manuscript (include quotes in quotation marks "like this" to indicate direct quotes from your manuscript), or elaborate on this item by providing additional information not in the ms, or briefly explain why the item is not applicable/relevant for your study

Study Design
A two-period double crossover randomized-controlled study was employed. The double-crossover study design mitigates learning bias and has been previously shown to improve statistical power [43] Patients were randomized in a 1:1 ratio to receive either the ACoE or paper-based test first, then returned 1-6 weeks later to receive the other test. Inter-test duration was limited to 1-6 weeks to control for time-, medication-, or pathology-related cognitive changes to balance for learning bias while also minimizing in disease or mental state between tests [37]. Only patients receiving the ACE-3 were randomized to enable comparison of the cognitive evaluation of the ACoE to the ACE-3 (Supplementary Figure 1). No changes were made to the study design after initiation.
Patients with and without cognitive complaints were recruited. Inclusion criterion was English fluency and being over age 18. English fluency was evaluated by the attending clinician. Exclusion criteria were acute medical conditions contributing to cognitive state, acute psychiatric disorders contributing to cognitive state, delirious states, or disabilities restricting ability to utilize screens, disabilities restricting ability to receive visual and auditory instructions, or developmental delay.

Subjects
Our study cohort comprised patients from neurology clinics across the Health Sciences Centre, University of Manitoba. Subjects who had previously consented to being contacted for research were contacted by study staff by phone for enrollment. Our overall cohort (n = 46) is composed of patients receiving both the ACE-3 and MoCA, with each group receiving the ACoE. To understand how the ACoE performs across a range of cognition and age states, we recruited patients across ranging from healthy controls to probable Alzheimer Disease, and a range of ages spanning 33 to 82 years of age. Further details are available (Table 1). The patients receiving the ACE-3 were randomized into two arms, with further details available for each arm (Supplementary Table 1).
An additional validation cohort of elderly patients from the Health Sciences Center, University of Manitoba, was recruited in a non-randomized fashion. These patients (n = 20) were all over the age of 65 with an age range spanning 67 to 86 years of age, and received the MoCA as well as the ACoE in a non-randomized fashion.

Patient Recruitment
Patients indicating interest in clinical research were contacted by study team members via phone. Interested patients were screened for inclusion and exclusion criteria and enrolled. This study was not blinded. At the first clinic visit, patients were again screened for inclusion/exclusion criteria by a physician.

Study Sample Size
Power analysis for this study was based on two separate analyses: 1) sample required to have powered assessment reliability of cognitive phenotyping, and 2) sample required to have powered assessment of screening performance.
To assess reliability of phenotyping, the intraclass correlation coefficient (ICC) is the primary metric used in validation of tools [44], including cognitive examinations [21,45,46]. Previous statistical analysis has been performed to evaluate the amount of subjects required to achieve powered analysis using the ICC in human research [47]. The power analysis published in this study demonstrates 35 subjects are required to achieve 80% statistical power. The three variables dictating the power analysis were number of [illegible]

observations (k), minimum ICC (ρ), and confidence interval precision (ω). Values were chosen according to our study design. Patients received two separate observations (ACoE observation and ACE-3 observation), setting k to 2. We chose a minimum acceptable ICC of 0.80, setting ρ to 0.80. Lastly, we chose maximum potential confidence interval half-widths of 0.15, setting ω to 0.15.
To assess screening performance, the area under the curve of the receiver operating characteristic (AUROC) is the measurement of choice. To assess sample size required for a powered analysis of the AUROC, we employed the Hanley and MacNeil formula [47]. We targeted 80% power. The required sample size for the AUROC varies with test performance. If a test has an AUROC of 0.70, 16 positive and negative cases are required, which decreases to 8 at an AUROC of 0.80, and 2 at an AUROC of 0.90. We recruited to a total of 16 positive and negative cases to account for the range of potential ACoE AUROC values.

Test Administration
Patients were tested in a quiet environment by a physician trained in cognitive examination. Caregivers were allowed to join but could not participate in the examination. A trained physician examiner directly administered the examination, evaluated responses, and summated scores.
Paper-based tests were administered via standard paper forms and evaluated using their established scoring guideline. The ACE-3 is composed of 19 questions spanning the domains of language, executive function, memory, and visuospatial function [40]. Language is separated into 'language' and 'fluency' on the ACE-3 for relevance to Parkinson Disease. The ACE-3 total score ranges from 0-100, with 100 representing maximum function. The MOCA is another ubiqituous test which briefly evaluates language, executive function, memory, and visuospatial function with 13 questions [42]. The total score of the MoCA ranges from 0-30, with 30 representing maximum function.
The ACoE was administered by touchscreen and microphone-equipped t. The ACoE administered itself to the patient without interference or prompting from the examiner. ACoE responses were automatically scored and summated.

The ACoE Evaluation of Cognition
The ACoE receives user input via any hardware device with an internet connection, microphone and touchscreen. The ACoE questions are answered via microphone and touchscreen, although a keyboard and mouse can be used. The user proceeds through an examination from end-end which automatically administers voiced instructions with closed captioning (Figure 1).
The ACoE consists of 19 questions within the primary domains of memory, language, fluency, visuospatial, and executive function (also referred to as attention). Attention comprises executive function and actual attention. This classification system of the cognitive functions is modeled after the ACE-3 [40] A table of each question and associated primary cognitive domain are available (Supplementary Table 2).
For each question, the patient is given unrestricted time to answer. The patient receives specific instructions both visually and by audio at the start of each question. The instructions were allowed to be repeated up to 3 times, but no further assistance is provided. 13 questions are answered with speech, 3 with touchscreen inputs of drawing or manipulating on-screen objects, 2 with dropdown menus, and 1 with typing.
At the end of the examination, total score and cognitive domain scores are calculated. There are 5 memory questions totaling 26 points, 1 fluency question totaling 14 points, 7 language questions totaling 40 points, 3 executive questions totaling 18 points, and 3 visuospatial questions totaling 16 points (Table 1). The total score totals 100 points.

questions totaling 16 points (Table 1). The total score totals 100 points.

Table 1. Breakdown of ACoE Scoring.

Cognitive Domain	Number of Questions	Total Score
Overall	19	100
Memory	4	26
Language	8	40
Fluency (language subdomain)	1	14
Executive Function	3	18
Visuospatial Function	3	16

ACoE Algorithms to Evaluate Patient Input

A unique algorithm was developed for each of the 19 primary questions, including sub-questions (Supplementary Table 3-21). This resulted in 76 unique algorithms, which have been previously described (Figure 1) [38,39,48,49] In brief, each algorithm corresponds to how one question on the ACE-3 is scored and attempts to estimate the scoring of the ACE-3 for the corresponding question. These span three primary algorithmic domains: computer vision, natural language processing, and expert algorithms.

The 3 computer vision algorithms enable testing of visuospatial drawing tasks. For example, the overlapping infinity copy, cube copy, and clock drawing test evaluated by the SketchNet, a custom convolutional neural network created for the ACoE [49] These are involved in visuospatial function evaluation. The computer vision algorithms are responsible for assessing 8/100 ACoE points.

The 48 natural language processing algorithms evaluate spoken answers, speech quality, sentence structure, and word pronunciation. The tasks these are responsible for are immediate recall, mental arithmetic, delayed word recall, phonemic list generation, semantic list generation, semantic memory, sentence writing, word repetition, sentence repetition, naming, reading aloud, counting, identifying and partially obscured objects for simultanagnosia. These are involved in evaluation of memory, language, executive, and visuospatial tasks. The natural language processing algorithms are responsible 68/100 ACoE points.

The 25 expert algorithms allow evaluation of complex tasks which do not easily fit into common machine learning approaches. Examples include an algorithm to evaluate a patient’s orientation to their location in space by combining natural language processing with geolocation. Similar algorithms are used to assess orientation to time, ability to follow on-screen commands, recognize objects, or recall with prompting. These are involved in the evaluation of memory, language, executive, and visuospatial tasks. The questions requiring expert algorithms compose 24/100 ACoE points.

ACoE Deployment

The ACoE is hosted on Amazon Web Services to provide accessibility to roughly 75% of the globe. The ACoE leverages a cloud-based format to enhance accessibility for patients, allow physicians to test patients regardless of their location, and to provide secure storage of data. Patients access the ACoE through links which are sent to them by an ACoE administrator. Each link is specific to the given patient and becomes inactive after use. Each link is validated upon use and invalid links are rejected access. The patient then receives the ACoE and results are sent via encryption to the scoring server which is stored on a private subnet. The scoring server then returns patient scores in an encrypted manner to the administrative user’s database. This allows the clinician using the ACoE’s administrative

administrative user's database. This allows the clinician using the ACoE's administrative platform to view patient results as they are completed. The raw files for each patient are stored in an anonymized format on and encrypted and private database. The ACoE and administrator platform are Health Information Privacy Protection Act compliant. A diagrammatic representation of the process is provided (Figure 2).

Evaluation of Cognitive Phenotyping Reliability
To evaluate ability of the ACoE to reliably phenotype overall cognition and cognitive subdomains, the ICC was used to compare the similarity of scores between each patient's ACoE and ACE-3 scores. To evaluate systematic deviances in group-level scores, we compared the central tendency of ACoE versus ACE-3 score with Wilcoxon signed-rank testing, a paired evaluator of the median.

Evaluation of Diagnostic Reliability
A receiver operating characteristic was constructed comparing the ACoE classifications to ACE-3 (n = 35) and MoCA (n = 11) classifications. AUROC was calculated as a metric of reliability in diagnosis. Youden's J was calculated to derive optimal classification threshold, enabling direct comparison of thresholds between ACoE and ACE-3 [51] To assess the confidence with which a classification of impairment can be made, bootstrapped sensitivity and specificity were calculated at all ACoE scores. Specifically, patients were resampled with replacement 10,000 times, which is a reliable method of generated confidence intervals [52,53] Labels of cognitively impaired versus intact were made by an expert clinician in conjunction with the ACE-3 and MoCA established cutoffs: 26/30 on the MoCA and 83/100 on the ACE-3 [40,42,54]

Statistics
All analyses were performed in Python. Central tendency, correlation, normality, and general linear model analyses were performed with Statsmodels. Power analyses were performed in accordance with established techniques, using a nomogram for intraclass correlation coefficient and a Python implementation [47] Intraclass correlation coefficient was calculated with the Pingouin package [55]
Spearman correlation was used for ordinal data. Paired Wilcoxon tests was employed for ordinal data and when normality was violated as measured by the Shapiro-Wilks test. Multiple comparisons were corrected with Bonferroni correction.
Two methods of intraclass correlation were performed. The two-way random effects model is commonly employed to evaluate agreement between clinical evaluations and scales, and is what we use to derive our primary ICC results [44] We focus on evaluating the consistency of the two raters, the ACoE and the ACE-3. To perform sensitivity analyses, we use the more conservative one-way random effects model [44]
A multivariate regression (ordinary least squares) was used to relate independent variables age, cognitive status, ethnicity, sex, educational status, and randomization group to the dependent variable: ACoE score. These variables were selected to specifically evaluate the effect of known demographic variables upon cognitive test scores.
An adjustment factor was developed to account for the effect of patient age upon their ACoE score derived from the multivariate regression relating age to ACoE score (Supplementary Equation 1). The coefficient of age from this formula can be used to adjust ACoE scores for age (Supplementary Equation 2).

Ethics Considerations
The study has been conducted in accordance with the ethical standards. This study was

The study has been conducted in accordance with the ethical standards. This study was conducted in accordance with ethical standards as described in the 1964 Declaration of Helsinki and its subsequent amendments. Approval was achieved by the Research Ethics Board of the Health Sciences Center, the University of Manitoba (#HS25666) for human subjects research. Informed consent was achieved for assessment of each patient and further analysis of results. Patients or their caregivers provided written consent at the first clinic visit. all signed Institutional Review Board-approved consent forms. Substitute decision makers were included in the consent process of cognitively impaired patients. All data was anonymized and de-identified. Patients were not compensated for involvement in this study.

3b) Important changes to methods after trial commencement (such as eligibility criteria), with reasons

Does your paper address CONSORT subitem 3b? *

Copy and paste relevant sections from the manuscript (include quotes in quotation marks "like this" to indicate direct quotes from your manuscript), or elaborate on this item by providing additional information not in the ms, or briefly explain why the item is not applicable/relevant for your study

Study Design
A two-period double crossover randomized-controlled study was employed. The double-crossover study design mitigates learning bias and has been previously shown to improve statistical power [43] Patients were randomized in a 1:1 ratio to receive either the ACoE or paper-based test first, then returned 1-6 weeks later to receive the other test. Inter-test duration was limited to 1-6 weeks to control for time-, medication-, or pathology-related cognitive changes to balance for learning bias while also minimizing in disease or mental state between tests [37]. Only patients receiving the ACE-3 were randomized to enable comparison of the cognitive evaluation of the ACoE to the ACE-3 (Supplementary Figure 1). No changes were made to the study design after initiation.
Patients with and without cognitive complaints were recruited. Inclusion criterion was English fluency and being over age 18. English fluency was evaluated by the attending clinician. Exclusion criteria were acute medical conditions contributing to cognitive state, acute psychiatric disorders contributing to cognitive state, delirious states, or disabilities restricting ability to utilize screens, disabilities restricting ability to receive visual and auditory instructions, or developmental delay.

Subjects
Our study cohort comprised patients from neurology clinics across the Health Sciences Centre, University of Manitoba. Subjects who had previously consented to being contacted for research were contacted by study staff by phone for enrollment. Our overall cohort (n = 46) is composed of patients receiving both the ACE-3 and MoCA, with each group receiving the ACoE. To understand how the ACoE performs across a range of cognition and age states, we recruited patients across ranging from healthy controls to probable Alzheimer Disease, and a range of ages spanning 33 to 82 years of age. Further details are available (Table 1). The patients receiving the ACE-3 were randomized into two arms, with further details available for each arm (Supplementary Table 1).
An additional validation cohort of elderly patients from the Health Sciences Center, University of Manitoba, was recruited in a non-randomized fashion. These patients (n = 20) were all over the age of 65 with an age range spanning 67 to 86 years of age, and received the MoCA as well as the ACoE in a non-randomized fashion.

Patient Recruitment
Patients indicating interest in clinical research were contacted by study team members via phone. Interested patients were screened for inclusion and exclusion criteria and enrolled. This study was not blinded. At the first clinic visit, patients were again screened for inclusion/exclusion criteria by a physician.

Study Sample Size
Power analysis for this study was based on two separate analyses: 1) sample required to have powered assessment reliability of cognitive phenotyping, and 2) sample required to have powered assessment of screening performance.
To assess reliability of phenotyping, the intraclass correlation coefficient (ICC) is the primary metric used in validation of tools [44], including cognitive examinations [21,45,46]. Previous statistical analysis has been performed to evaluate the amount of subjects required to achieve powered analysis using the ICC in human research [47]. The power analysis published in this study demonstrates 35 subjects are required to achieve 80% statistical power. The three variables dictating the power analysis were number of [illegible]

observations (k), minimum ICC (ρ), and confidence interval precision (ω). Values were chosen according to our study design. Patients received two separate observations (ACoE observation and ACE-3 observation), setting k to 2. We chose a minimum acceptable ICC of 0.80, setting ρ to 0.80. Lastly, we chose maximum potential confidence interval half-widths of 0.15, setting ω to 0.15.
To assess screening performance, the area under the curve of the receiver operating characteristic (AUROC) is the measurement of choice. To assess sample size required for a powered analysis of the AUROC, we employed the Hanley and MacNeil formula [47]. We targeted 80% power. The required sample size for the AUROC varies with test performance. If a test has an AUROC of 0.70, 16 positive and negative cases are required, which decreases to 8 at an AUROC of 0.80, and 2 at an AUROC of 0.90. We recruited to a total of 16 positive and negative cases to account for the range of potential ACoE AUROC values.

Test Administration
Patients were tested in a quiet environment by a physician trained in cognitive examination. Caregivers were allowed to join but could not participate in the examination. A trained physician examiner directly administered the examination, evaluated responses, and summated scores.
Paper-based tests were administered via standard paper forms and evaluated using their established scoring guideline. The ACE-3 is composed of 19 questions spanning the domains of language, executive function, memory, and visuospatial function [40]. Language is separated into 'language' and 'fluency' on the ACE-3 for relevance to Parkinson Disease. The ACE-3 total score ranges from 0-100, with 100 representing maximum function. The MOCA is another ubiqituous test which briefly evaluates language, executive function, memory, and visuospatial function with 13 questions [42]. The total score of the MoCA ranges from 0-30, with 30 representing maximum function.
The ACoE was administered by touchscreen and microphone-equipped t. The ACoE administered itself to the patient without interference or prompting from the examiner. ACoE responses were automatically scored and summated.

The ACoE Evaluation of Cognition
The ACoE receives user input via any hardware device with an internet connection, microphone and touchscreen. The ACoE questions are answered via microphone and touchscreen, although a keyboard and mouse can be used. The user proceeds through an examination from end-end which automatically administers voiced instructions with closed captioning (Figure 1).
The ACoE consists of 19 questions within the primary domains of memory, language, fluency, visuospatial, and executive function (also referred to as attention). Attention comprises executive function and actual attention. This classification system of the cognitive functions is modeled after the ACE-3 [40] A table of each question and associated primary cognitive domain are available (Supplementary Table 2).
For each question, the patient is given unrestricted time to answer. The patient receives specific instructions both visually and by audio at the start of each question. The instructions were allowed to be repeated up to 3 times, but no further assistance is provided. 13 questions are answered with speech, 3 with touchscreen inputs of drawing or manipulating on-screen objects, 2 with dropdown menus, and 1 with typing.
At the end of the examination, total score and cognitive domain scores are calculated. There are 5 memory questions totaling 26 points, 1 fluency question totaling 14 points, 7 language questions totaling 40 points, 3 executive questions totaling 18 points, and 3 visuospatial questions totaling 16 points (Table 1). The total score totals 100 points.

questions totaling 16 points (Table 1). The total score totals 100 points.

Table 1. Breakdown of ACoE Scoring.

Cognitive Domain	Number of Questions	Total Score
Overall	19	100
Memory	4	26
Language	8	40
Fluency (language subdomain)	1	14
Executive Function	3	18
Visuospatial Function	3	16

ACoE Algorithms to Evaluate Patient Input

A unique algorithm was developed for each of the 19 primary questions, including sub-questions (Supplementary Table 3-21). This resulted in 76 unique algorithms, which have been previously described (Figure 1) [38,39,48,49] In brief, each algorithm corresponds to how one question on the ACE-3 is scored and attempts to estimate the scoring of the ACE-3 for the corresponding question. These span three primary algorithmic domains: computer vision, natural language processing, and expert algorithms.

The 3 computer vision algorithms enable testing of visuospatial drawing tasks. For example, the overlapping infinity copy, cube copy, and clock drawing test evaluated by the SketchNet, a custom convolutional neural network created for the ACoE [49] These are involved in visuospatial function evaluation. The computer vision algorithms are responsible for assessing 8/100 ACoE points.

The 48 natural language processing algorithms evaluate spoken answers, speech quality, sentence structure, and word pronunciation. The tasks these are responsible for are immediate recall, mental arithmetic, delayed word recall, phonemic list generation, semantic list generation, semantic memory, sentence writing, word repetition, sentence repetition, naming, reading aloud, counting, identifying and partially obscured objects for simultanagnosia. These are involved in evaluation of memory, language, executive, and visuospatial tasks. The natural language processing algorithms are responsible 68/100 ACoE points.

The 25 expert algorithms allow evaluation of complex tasks which do not easily fit into common machine learning approaches. Examples include an algorithm to evaluate a patient's orientation to their location in space by combining natural language processing with geolocation. Similar algorithms are used to assess orientation to time, ability to follow on-screen commands, recognize objects, or recall with prompting. These are involved in the evaluation of memory, language, executive, and visuospatial tasks. The questions requiring expert algorithms compose 24/100 ACoE points.

ACoE Deployment

The ACoE is hosted on Amazon Web Services to provide accessibility to roughly 75% of the globe. The ACoE leverages a cloud-based format to enhance accessibility for patients, allow physicians to test patients regardless of their location, and to provide secure storage of data. Patients access the ACoE through links which are sent to them by an ACoE administrator. Each link is specific to the given patient and becomes inactive after use. Each link is validated upon use and invalid links are rejected access. The patient then receives the ACoE and results are sent via encryption to the scoring server which is stored on a private subnet. The scoring server then returns patient scores in an encrypted manner to the administrative user's database. This allows the clinician using the ACoE's administrative

administrative user's database. This allows the clinician using the ACoE's administrative platform to view patient results as they are completed. The raw files for each patient are stored in an anonymized format on and encrypted and private database. The ACoE and administrator platform are Health Information Privacy Protection Act compliant. A diagrammatic representation of the process is provided (Figure 2).

Evaluation of Cognitive Phenotyping Reliability
To evaluate ability of the ACoE to reliably phenotype overall cognition and cognitive subdomains, the ICC was used to compare the similarity of scores between each patient's ACoE and ACE-3 scores. To evaluate systematic deviances in group-level scores, we compared the central tendency of ACoE versus ACE-3 score with Wilcoxon signed-rank testing, a paired evaluator of the median.

3b-i) Bug fixes, Downtimes, Content Changes

Bug fixes, Downtimes, Content Changes: ehealth systems are often dynamic systems. A description of changes to methods therefore also includes important changes made on the intervention or comparator during the trial (e.g., major bug fixes or changes in the functionality or content) (5-iii) and other "unexpected events" that may have influenced study design such as staff changes, system failures/downtimes, etc. [2].

	1	2	3	4	5	
subitem not at all important	○	○	○	○	○	essential

Does your paper address subitem 3b-i?

Copy and paste relevant sections from the manuscript (include quotes in quotation marks "like this" to indicate direct quotes from your manuscript), or elaborate on this item by providing additional information not in the ms, or briefly explain why the item is not applicable/relevant for your study

Your answer

4a) Eligibility criteria for participants

Does your paper address CONSORT subitem 4a? *

Copy and paste relevant sections from the manuscript (include quotes in quotation marks "like this" to indicate direct quotes from your manuscript), or elaborate on this item by providing additional information not in the ms, or briefly explain why the item is not applicable/relevant for your study

Study Design
A two-period double crossover randomized-controlled study was employed. The double-crossover study design mitigates learning bias and has been previously shown to improve statistical power [43] Patients were randomized in a 1:1 ratio to receive either the ACoE or paper-based test first, then returned 1-6 weeks later to receive the other test. Inter-test duration was limited to 1-6 weeks to control for time-, medication-, or pathology-related cognitive changes to balance for learning bias while also minimizing in disease or mental state between tests [37]. Only patients receiving the ACE-3 were randomized to enable comparison of the cognitive evaluation of the ACoE to the ACE-3 (Supplementary Figure 1). No changes were made to the study design after initiation.
Patients with and without cognitive complaints were recruited. Inclusion criterion was English fluency and being over age 18. English fluency was evaluated by the attending clinician. Exclusion criteria were acute medical conditions contributing to cognitive state, acute psychiatric disorders contributing to cognitive state, delirious states, or disabilities restricting ability to utilize screens, disabilities restricting ability to receive visual and auditory instructions, or developmental delay.

Subjects
Our study cohort comprised patients from neurology clinics across the Health Sciences Centre, University of Manitoba. Subjects who had previously consented to being contacted for research were contacted by study staff by phone for enrollment. Our overall cohort (n = 46) is composed of patients receiving both the ACE-3 and MoCA, with each group receiving the ACoE. To understand how the ACoE performs across a range of cognition and age states, we recruited patients across ranging from healthy controls to probable Alzheimer Disease, and a range of ages spanning 33 to 82 years of age. Further details are available (Table 1). The patients receiving the ACE-3 were randomized into two arms, with further details available for each arm (Supplementary Table 1).
An additional validation cohort of elderly patients from the Health Sciences Center, University of Manitoba, was recruited in a non-randomized fashion. These patients (n = 20) were all over the age of 65 with an age range spanning 67 to 86 years of age, and received the MoCA as well as the ACoE in a non-randomized fashion.

Patient Recruitment
Patients indicating interest in clinical research were contacted by study team members via phone. Interested patients were screened for inclusion and exclusion criteria and enrolled. This study was not blinded. At the first clinic visit, patients were again screened for inclusion/exclusion criteria by a physician.

4a-i) Computer / Internet literacy

Computer / Internet literacy is often an implicit “de facto” eligibility criterion - this should be explicitly clarified.

	1	2	3	4	5	
subitem not at all important	○	○	○	○	○	essential

Does your paper address subitem 4a-i?

Copy and paste relevant sections from the manuscript (include quotes in quotation marks "like this" to indicate direct quotes from your manuscript), or elaborate on this item by providing additional information not in the ms, or briefly explain why the item is not applicable/relevant for your study

Your answer

4a-ii) Open vs. closed, web-based vs. face-to-face assessments:

Open vs. closed, web-based vs. face-to-face assessments: Mention how participants were recruited (online vs. offline), e.g., from an open access website or from a clinic, and clarify if this was a purely web-based trial, or there were face-to-face components (as part of the intervention or for assessment), i.e., to what degree got the study team to know the participant. In online-only trials, clarify if participants were quasi-anonymous and whether having multiple identities was possible or whether technical or logistical measures (e.g., cookies, email confirmation, phone calls) were used to detect/prevent these.

	1	2	3	4	5	
subitem not at all important	○	○	○	○	○	essential

Does your paper address subitem 4a-ii? *

Copy and paste relevant sections from the manuscript (include quotes in quotation marks "like this" to indicate direct quotes from your manuscript), or elaborate on this item by providing additional information not in the ms, or briefly explain why the item is not applicable/relevant for your study

Test Administration
Patients were tested in a quiet environment by a physician trained in cognitive examination. Caregivers were allowed to join but could not participate in the examination. A trained physician examiner directly administered the examination, evaluated responses, and summated scores.
Paper-based tests were administered via standard paper forms and evaluated using their established scoring guideline. The ACE-3 is composed of 19 questions spanning the domains of language, executive function, memory, and visuospatial function [40]. Language is separated into 'language' and 'fluency' on the ACE-3 for relevance to Parkinson Disease. The ACE-3 total score ranges from 0-100, with 100 representing maximum function. The MOCA is another ubiqituous test which briefly evaluates language, executive function, memory, and visuospatial function with 13 questions [42]. The total score of the MoCA ranges from 0-30, with 30 representing maximum function.
The ACoE was administered by touchscreen and microphone-equipped t. The ACoE administered itself to the patient without interference or prompting from the examiner. ACoE responses were automatically scored and summated.

4a-iii) Information giving during recruitment

Information given during recruitment. Specify how participants were briefed for recruitment and in the informed consent procedures (e.g., publish the informed consent documentation as appendix, see also item X26), as this information may have an effect on user self-selection, user expectation and may also bias results.

	1	2	3	4	5	
subitem not at all important	○	○	○	○	○	essential

Does your paper address subitem 4a-iii?

Copy and paste relevant sections from the manuscript (include quotes in quotation marks "like this" to indicate direct quotes from your manuscript), or elaborate on this item by providing additional information not in the ms, or briefly explain why the item is not applicable/relevant for your study

Your answer

4b) Settings and locations where the data were collected

Does your paper address CONSORT subitem 4b? *

Copy and paste relevant sections from the manuscript (include quotes in quotation marks "like this" to indicate direct quotes from your manuscript), or elaborate on this item by providing additional information not in the ms, or briefly explain why the item is not applicable/relevant for your study

Test Administration
Patients were tested in a quiet environment by a physician trained in cognitive examination. Caregivers were allowed to join but could not participate in the examination. A trained physician examiner directly administered the examination, evaluated responses, and summated scores.
Paper-based tests were administered via standard paper forms and evaluated using their established scoring guideline. The ACE-3 is composed of 19 questions spanning the domains of language, executive function, memory, and visuospatial function [40]. Language is separated into 'language' and 'fluency' on the ACE-3 for relevance to Parkinson Disease. The ACE-3 total score ranges from 0-100, with 100 representing maximum function. The MOCA is another ubiqituous test which briefly evaluates language, executive function, memory, and visuospatial function with 13 questions [42]. The total score of the MoCA ranges from 0-30, with 30 representing maximum function.
The ACoE was administered by touchscreen and microphone-equipped t. The ACoE administered itself to the patient without interference or prompting from the examiner. ACoE responses were automatically scored and summated.

4b-i) Report if outcomes were (self-)assessed through online questionnaires

Clearly report if outcomes were (self-)assessed through online questionnaires (as common in web-based trials) or otherwise.

	1	2	3	4	5	
subitem not at all important	○	○	○	○	○	essential

Does your paper address subitem 4b-i? *

Copy and paste relevant sections from the manuscript (include quotes in quotation marks "like this" to indicate direct quotes from your manuscript), or elaborate on this item by providing additional information not in the ms, or briefly explain why the item is not applicable/relevant for your study

Test Administration
Patients were tested in a quiet environment by a physician trained in cognitive examination. Caregivers were allowed to join but could not participate in the examination. A trained physician examiner directly administered the examination, evaluated responses, and summated scores.
Paper-based tests were administered via standard paper forms and evaluated using their established scoring guideline. The ACE-3 is composed of 19 questions spanning the domains of language, executive function, memory, and visuospatial function [40]. Language is separated into 'language' and 'fluency' on the ACE-3 for relevance to Parkinson Disease. The ACE-3 total score ranges from 0-100, with 100 representing maximum function. The MOCA is another ubiqituous test which briefly evaluates language, executive function, memory, and visuospatial function with 13 questions [42]. The total score of the MoCA ranges from 0-30, with 30 representing maximum function.
The ACoE was administered by touchscreen and microphone-equipped t. The ACoE administered itself to the patient without interference or prompting from the examiner. ACoE responses were automatically scored and summated.

4b-ii) Report how institutional affiliations are displayed

Report how institutional affiliations are displayed to potential participants [on ehealth media], as affiliations with prestigious hospitals or universities may affect volunteer rates, use, and reactions with regards to an intervention.(Not a required item – describe only if this may bias results)

	1	2	3	4	5	
subitem not at all important	○	○	○	○	○	essential

Does your paper address subitem 4b-ii?

Copy and paste relevant sections from the manuscript (include quotes in quotation marks "like this" to indicate direct quotes from your manuscript), or elaborate on this item by providing additional information not in the ms, or briefly explain why the item is not applicable/relevant for your study

Your answer

5) The interventions for each group with sufficient details to allow replication, including how and when they were actually administered

5-i) Mention names, credential, affiliations of the developers, sponsors, and owners

Mention names, credential, affiliations of the developers, sponsors, and owners [6] (if authors/evaluators are owners or developer of the software, this needs to be declared in a “Conflict of interest” section or mentioned elsewhere in the manuscript).

	1	2	3	4	5	
subitem not at all important	○	○	○	○	○	essential

Does your paper address subitem 5-i?

Copy and paste relevant sections from the manuscript (include quotes in quotation marks "like this" to indicate direct quotes from your manuscript), or elaborate on this item by providing additional information not in the ms, or briefly explain why the item is not applicable/relevant for your study

Your answer

5-ii) Describe the history/development process

Describe the history/development process of the application and previous formative evaluations (e.g., focus groups, usability testing), as these will have an impact on adoption/use rates and help with interpreting results.

	1	2	3	4	5	
subitem not at all important	○	○	○	○	○	essential

Does your paper address subitem 5-ii?

Copy and paste relevant sections from the manuscript (include quotes in quotation marks "like this" to indicate direct quotes from your manuscript), or elaborate on this item by providing additional information not in the ms, or briefly explain why the item is not applicable/relevant for your study

Your answer

5-iii) Revisions and updating

Revisions and updating. Clearly mention the date and/or version number of the application/intervention (and comparator, if applicable) evaluated, or describe whether the intervention underwent major changes during the evaluation process, or whether the development and/or content was “frozen” during the trial. Describe dynamic components such as news feeds or changing content which may have an impact on the replicability of the intervention (for unexpected events see item 3b).

	1	2	3	4	5	
subitem not at all important	○	○	○	○	○	essential

Does your paper address subitem 5-iii?

Copy and paste relevant sections from the manuscript (include quotes in quotation marks "like this" to indicate direct quotes from your manuscript), or elaborate on this item by providing additional information not in the ms, or briefly explain why the item is not applicable/relevant for your study

Your answer

5-iv) Quality assurance methods

Provide information on quality assurance methods to ensure accuracy and quality of information provided [1], if applicable.

	1	2	3	4	5	
subitem not at all important	○	○	○	○	○	essential

Does your paper address subitem 5-iv?

Copy and paste relevant sections from the manuscript (include quotes in quotation marks "like this" to indicate direct quotes from your manuscript), or elaborate on this item by providing additional information not in the ms, or briefly explain why the item is not applicable/relevant for your study

Your answer

5-v) Ensure replicability by publishing the source code, and/or providing screenshots/screen-capture video, and/or providing flowcharts of the algorithms used

Ensure replicability by publishing the source code, and/or providing screenshots/screen-capture video, and/or providing flowcharts of the algorithms used. Replicability (i.e., other researchers should in principle be able to replicate the study) is a hallmark of scientific reporting.

	1	2	3	4	5	
subitem not at all important	○	○	○	○	○	essential

Does your paper address subitem 5-v?

Copy and paste relevant sections from the manuscript (include quotes in quotation marks "like this" to indicate direct quotes from your manuscript), or elaborate on this item by providing additional information not in the ms, or briefly explain why the item is not applicable/relevant for your study

Your answer

5-vi) Digital preservation

Digital preservation: Provide the URL of the application, but as the intervention is likely to change or disappear over the course of the years; also make sure the intervention is archived (Internet Archive, webcitation.org, and/or publishing the source code or screenshots/videos alongside the article). As pages behind login screens cannot be archived, consider creating demo pages which are accessible without login.

	1	2	3	4	5	
subitem not at all important	○	○	○	○	○	essential

Does your paper address subitem 5-vi?

Copy and paste relevant sections from the manuscript (include quotes in quotation marks "like this" to indicate direct quotes from your manuscript), or elaborate on this item by providing additional information not in the ms, or briefly explain why the item is not applicable/relevant for your study

Your answer

5-vii) Access

Access: Describe how participants accessed the application, in what setting/context, if they had to pay (or were paid) or not, whether they had to be a member of specific group. If known, describe how participants obtained “access to the platform and Internet” [1]. To ensure access for editors/reviewers/readers, consider to provide a “backdoor” login account or demo mode for reviewers/readers to explore the application (also important for archiving purposes, see vi).

	1	2	3	4	5	
subitem not at all important	○	○	○	○	○	essential

Does your paper address subitem 5-vii? *

Copy and paste relevant sections from the manuscript (include quotes in quotation marks "like this" to indicate direct quotes from your manuscript), or elaborate on this item by providing additional information not in the ms, or briefly explain why the item is not applicable/relevant for your study

Your answer

5-viii) Mode of delivery, features/functionalities/components of the intervention and comparator, and the theoretical framework

Describe mode of delivery, features/functionalities/components of the intervention and comparator, and the theoretical framework [6] used to design them (instructional strategy [1], behaviour change techniques, persuasive features, etc., see e.g., [7, 8] for terminology). This includes an in-depth description of the content (including where it is coming from and who developed it) [1],” whether [and how] it is tailored to individual circumstances and allows users to track their progress and receive feedback” [6]. This also includes a description of communication delivery channels and – if computer-mediated communication is a component – whether communication was synchronous or asynchronous [6]. It also includes information on presentation strategies [1], including page design principles, average amount of text on pages, presence of hyperlinks to other resources, etc. [1].

	1	2	3	4	5	
subitem not at all important	○	○	○	○	○	essential

Does your paper address subitem 5-viii? *

Copy and paste relevant sections from the manuscript (include quotes in quotation marks "like this" to indicate direct quotes from your manuscript), or elaborate on this item by providing additional information not in the ms, or briefly explain why the item is not applicable/relevant for your study

Test Administration
Patients were tested in a quiet environment by a physician trained in cognitive examination. Caregivers were allowed to join but could not participate in the examination. A trained physician examiner directly administered the examination, evaluated responses, and summated scores.
Paper-based tests were administered via standard paper forms and evaluated using their established scoring guideline. The ACE-3 is composed of 19 questions spanning the domains of language, executive function, memory, and visuospatial function [40]. Language is separated into 'language' and 'fluency' on the ACE-3 for relevance to Parkinson Disease. The ACE-3 total score ranges from 0-100, with 100 representing maximum function. The MOCA is another ubiqituous test which briefly evaluates language, executive function, memory, and visuospatial function with 13 questions [42]. The total score of the MoCA ranges from 0-30, with 30 representing maximum function.
The ACoE was administered by touchscreen and microphone-equipped t. The ACoE administered itself to the patient without interference or prompting from the examiner. ACoE responses were automatically scored and summated.

5-ix) Describe use parameters

Describe use parameters (e.g., intended "doses" and optimal timing for use). Clarify what instructions or recommendations were given to the user, e.g., regarding timing, frequency, heaviness of use, if any, or was the intervention used ad libitum.

	1	2	3	4	5	
subitem not at all important	○	○	○	○	○	essential

Does your paper address subitem 5-ix?

Copy and paste relevant sections from the manuscript (include quotes in quotation marks "like this" to indicate direct quotes from your manuscript), or elaborate on this item by providing additional information not in the ms, or briefly explain why the item is not applicable/relevant for your study

Your answer

5-x) Clarify the level of human involvement

Clarify the level of human involvement (care providers or health professionals, also technical assistance) in the e-intervention or as co-intervention (detail number and expertise of professionals involved, if any, as well as “type of assistance offered, the timing and frequency of the support, how it is initiated, and the medium by which the assistance is delivered”. It may be necessary to distinguish between the level of human involvement required for the trial, and the level of human involvement required for a routine application outside of a RCT setting (discuss under item 21 – generalizability).

	1	2	3	4	5	
subitem not at all important	○	○	○	○	○	essential

Does your paper address subitem 5-x?

Copy and paste relevant sections from the manuscript (include quotes in quotation marks "like this" to indicate direct quotes from your manuscript), or elaborate on this item by providing additional information not in the ms, or briefly explain why the item is not applicable/relevant for your study

Your answer

5-xi) Report any prompts/reminders used

Report any prompts/reminders used: Clarify if there were prompts (letters, emails, phone calls, SMS) to use the application, what triggered them, frequency etc. It may be necessary to distinguish between the level of prompts/reminders required for the trial, and the level of prompts/reminders for a routine application outside of a RCT setting (discuss under item 21 – generalizability).

	1	2	3	4	5	
subitem not at all important	○	○	○	○	○	essential

Does your paper address subitem 5-xi? *

Copy and paste relevant sections from the manuscript (include quotes in quotation marks "like this" to indicate direct quotes from your manuscript), or elaborate on this item by providing additional information not in the ms, or briefly explain why the item is not applicable/relevant for your study

Test Administration
Patients were tested in a quiet environment by a physician trained in cognitive examination. Caregivers were allowed to join but could not participate in the examination. A trained physician examiner directly administered the examination, evaluated responses, and summated scores.
Paper-based tests were administered via standard paper forms and evaluated using their established scoring guideline. The ACE-3 is composed of 19 questions spanning the domains of language, executive function, memory, and visuospatial function [40]. Language is separated into 'language' and 'fluency' on the ACE-3 for relevance to Parkinson Disease. The ACE-3 total score ranges from 0-100, with 100 representing maximum function. The MOCA is another ubiqituous test which briefly evaluates language, executive function, memory, and visuospatial function with 13 questions [42]. The total score of the MoCA ranges from 0-30, with 30 representing maximum function.
The ACoE was administered by touchscreen and microphone-equipped t. The ACoE administered itself to the patient without interference or prompting from the examiner. ACoE responses were automatically scored and summated.

5-xii) Describe any co-interventions (incl. training/support)

Describe any co-interventions (incl. training/support): Clearly state any interventions that are provided in addition to the targeted eHealth intervention, as ehealth intervention may not be designed as stand-alone intervention. This includes training sessions and support [1]. It may be necessary to distinguish between the level of training required for the trial, and the level of training for a routine application outside of a RCT setting (discuss under item 21 – generalizability.

	1	2	3	4	5	
subitem not at all important	○	○	○	○	○	essential

Does your paper address subitem 5-xii? *

Copy and paste relevant sections from the manuscript (include quotes in quotation marks "like this" to indicate direct quotes from your manuscript), or elaborate on this item by providing additional information not in the ms, or briefly explain why the item is not applicable/relevant for your study

Test Administration
Patients were tested in a quiet environment by a physician trained in cognitive examination. Caregivers were allowed to join but could not participate in the examination. A trained physician examiner directly administered the examination, evaluated responses, and summated scores.
Paper-based tests were administered via standard paper forms and evaluated using their established scoring guideline. The ACE-3 is composed of 19 questions spanning the domains of language, executive function, memory, and visuospatial function [40]. Language is separated into 'language' and 'fluency' on the ACE-3 for relevance to Parkinson Disease. The ACE-3 total score ranges from 0-100, with 100 representing maximum function. The MOCA is another ubiqituous test which briefly evaluates language, executive function, memory, and visuospatial function with 13 questions [42]. The total score of the MoCA ranges from 0-30, with 30 representing maximum function.
The ACoE was administered by touchscreen and microphone-equipped t. The ACoE administered itself to the patient without interference or prompting from the examiner. ACoE responses were automatically scored and summated.

6a) Completely defined pre-specified primary and secondary outcome measures, including how and when they were assessed

Does your paper address CONSORT subitem 6a? *

Copy and paste relevant sections from the manuscript (include quotes in quotation marks "like this" to indicate direct quotes from your manuscript), or elaborate on this item by providing additional information not in the ms, or briefly explain why the item is not applicable/relevant for your study

Your answer

6a-i) Online questionnaires: describe if they were validated for online use and apply CHERRIES items to describe how the questionnaires were designed/deployed

If outcomes were obtained through online questionnaires, describe if they were validated for online use and apply CHERRIES items to describe how the questionnaires were designed/deployed [9].

	1	2	3	4	5	
subitem not at all important	○	○	○	○	○	essential

Does your paper address subitem 6a-i?

Copy and paste relevant sections from manuscript text

Your answer

6a-ii) Describe whether and how “use” (including intensity of use/dosage) was defined/measured/monitored

Describe whether and how “use” (including intensity of use/dosage) was defined/measured/monitored (logins, logfile analysis, etc.). Use/adoption metrics are important process outcomes that should be reported in any ehealth trial.

	1	2	3	4	5	
subitem not at all important	○	○	○	○	○	essential

Does your paper address subitem 6a-ii?

Copy and paste relevant sections from manuscript text

Your answer

6a-iii) Describe whether, how, and when qualitative feedback from participants was obtained

Describe whether, how, and when qualitative feedback from participants was obtained (e.g., through emails, feedback forms, interviews, focus groups).

	1	2	3	4	5	
subitem not at all important	○	○	○	○	○	essential

Does your paper address subitem 6a-iii?

Copy and paste relevant sections from manuscript text

Your answer

6b) Any changes to trial outcomes after the trial commenced, with reasons

Does your paper address CONSORT subitem 6b? *

Copy and paste relevant sections from the manuscript (include quotes in quotation marks "like this" to indicate direct quotes from your manuscript), or elaborate on this item by providing additional information not in the ms, or briefly explain why the item is not applicable/relevant for your study

Study Design
A two-period double crossover randomized-controlled study was employed. The double-crossover study design mitigates learning bias and has been previously shown to improve statistical power [43] Patients were randomized in a 1:1 ratio to receive either the ACoE or paper-based test first, then returned 1-6 weeks later to receive the other test. Inter-test duration was limited to 1-6 weeks to control for time-, medication-, or pathology-related cognitive changes to balance for learning bias while also minimizing in disease or mental state between tests [37]. Only patients receiving the ACE-3 were randomized to enable comparison of the cognitive evaluation of the ACoE to the ACE-3 (Supplementary Figure 1). No changes were made to the study design after initiation.
Patients with and without cognitive complaints were recruited. Inclusion criterion was English fluency and being over age 18. English fluency was evaluated by the attending clinician. Exclusion criteria were acute medical conditions contributing to cognitive state, acute psychiatric disorders contributing to cognitive state, delirious states, or disabilities restricting ability to utilize screens, disabilities restricting ability to receive visual and auditory instructions, or developmental delay.

7a) How sample size was determined

NPT: When applicable, details of whether and how the clustering by care provides or centers was addressed

7a-i) Describe whether and how expected attrition was taken into account when calculating the sample size

Describe whether and how expected attrition was taken into account when calculating the sample size.

	1	2	3	4	5	
subitem not at all important	○	○	○	○	○	essential

Does your paper address subitem 7a-i?

Copy and paste relevant sections from manuscript title (include quotes in quotation marks "like this" to indicate direct quotes from your manuscript), or elaborate on this item by providing additional information not in the ms, or briefly explain why the item is not applicable/relevant for your study

Your answer

7b) When applicable, explanation of any interim analyses and stopping guidelines

Does your paper address CONSORT subitem 7b? *

Copy and paste relevant sections from the manuscript (include quotes in quotation marks "like this" to indicate direct quotes from your manuscript), or elaborate on this item by providing additional information not in the ms, or briefly explain why the item is not applicable/relevant for your study

Study Design
A two-period double crossover randomized-controlled study was employed. The double-crossover study design mitigates learning bias and has been previously shown to improve statistical power [43] Patients were randomized in a 1:1 ratio to receive either the ACoE or paper-based test first, then returned 1-6 weeks later to receive the other test. Inter-test duration was limited to 1-6 weeks to control for time-, medication-, or pathology-related cognitive changes to balance for learning bias while also minimizing in disease or mental state between tests [37]. Only patients receiving the ACE-3 were randomized to enable comparison of the cognitive evaluation of the ACoE to the ACE-3 (Supplementary Figure 1). No changes were made to the study design after initiation.
Patients with and without cognitive complaints were recruited. Inclusion criterion was English fluency and being over age 18. English fluency was evaluated by the attending clinician. Exclusion criteria were acute medical conditions contributing to cognitive state, acute psychiatric disorders contributing to cognitive state, delirious states, or disabilities restricting ability to utilize screens, disabilities restricting ability to receive visual and auditory instructions, or developmental delay.

8a) Method used to generate the random allocation sequence

NPT: When applicable, how care providers were allocated to each trial group

Does your paper address CONSORT subitem 8a? *

Copy and paste relevant sections from the manuscript (include quotes in quotation marks "like this" to indicate direct quotes from your manuscript), or elaborate on this item by providing additional information not in the ms, or briefly explain why the item is not applicable/relevant for your study

Study Design
A two-period double crossover randomized-controlled study was employed. The double-crossover study design mitigates learning bias and has been previously shown to improve statistical power [43] Patients were randomized in a 1:1 ratio to receive either the ACoE or paper-based test first, then returned 1-6 weeks later to receive the other test. Inter-test duration was limited to 1-6 weeks to control for time-, medication-, or pathology-related cognitive changes to balance for learning bias while also minimizing in disease or mental state between tests [37]. Only patients receiving the ACE-3 were randomized to enable comparison of the cognitive evaluation of the ACoE to the ACE-3 (Supplementary Figure 1). No changes were made to the study design after initiation.
Patients with and without cognitive complaints were recruited. Inclusion criterion was English fluency and being over age 18. English fluency was evaluated by the attending clinician. Exclusion criteria were acute medical conditions contributing to cognitive state, acute psychiatric disorders contributing to cognitive state, delirious states, or disabilities restricting ability to utilize screens, disabilities restricting ability to receive visual and auditory instructions, or developmental delay.

8b) Type of randomisation; details of any restriction (such as blocking and block size)

Does your paper address CONSORT subitem 8b? *

Copy and paste relevant sections from the manuscript (include quotes in quotation marks "like this" to indicate direct quotes from your manuscript), or elaborate on this item by providing additional information not in the ms, or briefly explain why the item is not applicable/relevant for your study

Study Design
A two-period double crossover randomized-controlled study was employed. The double-crossover study design mitigates learning bias and has been previously shown to improve statistical power [43] Patients were randomized in a 1:1 ratio to receive either the ACoE or paper-based test first, then returned 1-6 weeks later to receive the other test. Inter-test duration was limited to 1-6 weeks to control for time-, medication-, or pathology-related cognitive changes to balance for learning bias while also minimizing in disease or mental state between tests [37]. Only patients receiving the ACE-3 were randomized to enable comparison of the cognitive evaluation of the ACoE to the ACE-3 (Supplementary Figure 1). No changes were made to the study design after initiation.
Patients with and without cognitive complaints were recruited. Inclusion criterion was English fluency and being over age 18. English fluency was evaluated by the attending clinician. Exclusion criteria were acute medical conditions contributing to cognitive state, acute psychiatric disorders contributing to cognitive state, delirious states, or disabilities restricting ability to utilize screens, disabilities restricting ability to receive visual and auditory instructions, or developmental delay.

9) Mechanism used to implement the random allocation sequence (such as sequentially numbered containers), describing any steps taken to conceal the sequence until interventions were assigned

Does your paper address CONSORT subitem 9? *

Copy and paste relevant sections from the manuscript (include quotes in quotation marks "like this" to indicate direct quotes from your manuscript), or elaborate on this item by providing additional information not in the ms, or briefly explain why the item is not applicable/relevant for your study

Study Design
A two-period double crossover randomized-controlled study was employed. The double-crossover study design mitigates learning bias and has been previously shown to improve statistical power [43] Patients were randomized in a 1:1 ratio to receive either the ACoE or paper-based test first, then returned 1-6 weeks later to receive the other test. Inter-test duration was limited to 1-6 weeks to control for time-, medication-, or pathology-related cognitive changes to balance for learning bias while also minimizing in disease or mental state between tests [37]. Only patients receiving the ACE-3 were randomized to enable comparison of the cognitive evaluation of the ACoE to the ACE-3 (Supplementary Figure 1). No changes were made to the study design after initiation.
Patients with and without cognitive complaints were recruited. Inclusion criterion was English fluency and being over age 18. English fluency was evaluated by the attending clinician. Exclusion criteria were acute medical conditions contributing to cognitive state, acute psychiatric disorders contributing to cognitive state, delirious states, or disabilities restricting ability to utilize screens, disabilities restricting ability to receive visual and auditory instructions, or developmental delay.

10) Who generated the random allocation sequence, who enrolled participants, and who assigned participants to interventions

Does your paper address CONSORT subitem 10? *

Copy and paste relevant sections from the manuscript (include quotes in quotation marks "like this" to indicate direct quotes from your manuscript), or elaborate on this item by providing additional information not in the ms, or briefly explain why the item is not applicable/relevant for your study

Study Design
A two-period double crossover randomized-controlled study was employed. The double-crossover study design mitigates learning bias and has been previously shown to improve statistical power [43] Patients were randomized in a 1:1 ratio to receive either the ACoE or paper-based test first, then returned 1-6 weeks later to receive the other test. Inter-test duration was limited to 1-6 weeks to control for time-, medication-, or pathology-related cognitive changes to balance for learning bias while also minimizing in disease or mental state between tests [37]. Only patients receiving the ACE-3 were randomized to enable comparison of the cognitive evaluation of the ACoE to the ACE-3 (Supplementary Figure 1). No changes were made to the study design after initiation.
Patients with and without cognitive complaints were recruited. Inclusion criterion was English fluency and being over age 18. English fluency was evaluated by the attending clinician. Exclusion criteria were acute medical conditions contributing to cognitive state, acute psychiatric disorders contributing to cognitive state, delirious states, or disabilities restricting ability to utilize screens, disabilities restricting ability to receive visual and auditory instructions, or developmental delay.

11a) If done, who was blinded after assignment to interventions (for example, participants, care providers, those assessing outcomes) and how

NPT: Whether or not administering co-interventions were blinded to group assignment

11a-i) Specify who was blinded, and who wasn’t

Specify who was blinded, and who wasn’t. Usually, in web-based trials it is not possible to blind the participants [1, 3] (this should be clearly acknowledged), but it may be possible to blind outcome assessors, those doing data analysis or those administering co-interventions (if any).

	1	2	3	4	5	
subitem not at all important	○	○	○	○	○	essential

Does your paper address subitem 11a-i? *

Copy and paste relevant sections from the manuscript (include quotes in quotation marks "like this" to indicate direct quotes from your manuscript), or elaborate on this item by providing additional information not in the ms, or briefly explain why the item is not applicable/relevant for your study

Study Design
A two-period double crossover randomized-controlled study was employed. The double-crossover study design mitigates learning bias and has been previously shown to improve statistical power [43] Patients were randomized in a 1:1 ratio to receive either the ACoE or paper-based test first, then returned 1-6 weeks later to receive the other test. Inter-test duration was limited to 1-6 weeks to control for time-, medication-, or pathology-related cognitive changes to balance for learning bias while also minimizing in disease or mental state between tests [37]. Only patients receiving the ACE-3 were randomized to enable comparison of the cognitive evaluation of the ACoE to the ACE-3 (Supplementary Figure 1). No changes were made to the study design after initiation.
Patients with and without cognitive complaints were recruited. Inclusion criterion was English fluency and being over age 18. English fluency was evaluated by the attending clinician. Exclusion criteria were acute medical conditions contributing to cognitive state, acute psychiatric disorders contributing to cognitive state, delirious states, or disabilities restricting ability to utilize screens, disabilities restricting ability to receive visual and auditory instructions, or developmental delay.

11a-ii) Discuss e.g., whether participants knew which intervention was the “intervention of interest” and which one was the “comparator”

Informed consent procedures (4a-ii) can create biases and certain expectations - discuss e.g., whether participants knew which intervention was the “intervention of interest” and which one was the “comparator”.

	1	2	3	4	5	
subitem not at all important	○	○	○	○	○	essential

Does your paper address subitem 11a-ii?

Copy and paste relevant sections from the manuscript (include quotes in quotation marks "like this" to indicate direct quotes from your manuscript), or elaborate on this item by providing additional information not in the ms, or briefly explain why the item is not applicable/relevant for your study

Your answer

11b) If relevant, description of the similarity of interventions

(this item is usually not relevant for ehealth trials as it refers to similarity of a placebo or sham intervention to a active medication/intervention)

Does your paper address CONSORT subitem 11b? *

Copy and paste relevant sections from the manuscript (include quotes in quotation marks "like this" to indicate direct quotes from your manuscript), or elaborate on this item by providing additional information not in the ms, or briefly explain why the item is not applicable/relevant for your study

Your answer

12a) Statistical methods used to compare groups for primary and secondary outcomes

NPT: When applicable, details of whether and how the clustering by care providers or centers was addressed

Does your paper address CONSORT subitem 12a? *

Copy and paste relevant sections from the manuscript (include quotes in quotation marks "like this" to indicate direct quotes from your manuscript), or elaborate on this item by providing additional information not in the ms, or briefly explain why the item is not applicable/relevant for your study

Your answer

12a-i) Imputation techniques to deal with attrition / missing values

Imputation techniques to deal with attrition / missing values: Not all participants will use the intervention/comparator as intended and attrition is typically high in ehealth trials. Specify how participants who did not use the application or dropped out from the trial were treated in the statistical analysis (a complete case analysis is strongly discouraged, and simple imputation techniques such as LOCF may also be problematic [4]).

	1	2	3	4	5	
subitem not at all important	○	○	○	○	○	essential

Does your paper address subitem 12a-i? *

Copy and paste relevant sections from the manuscript (include quotes in quotation marks "like this" to indicate direct quotes from your manuscript), or elaborate on this item by providing additional information not in the ms, or briefly explain why the item is not applicable/relevant for your study

A two-period double crossover randomized-controlled study was employed. The double-crossover study design mitigates learning bias and has been previously shown to improve statistical power [43] Patients were randomized in a 1:1 ratio to receive either the ACoE or paper-based test first, then returned 1-6 weeks later to receive the other test. Inter-test duration was limited to 1-6 weeks to control for time-, medication-, or pathology-related cognitive changes to balance for learning bias while also minimizing in disease or mental state between tests [37]. Only patients receiving the ACE-3 were randomized to enable comparison of the cognitive evaluation of the ACoE to the ACE-3 (Supplementary Figure 1). No changes were made to the study design after initiation.

12b) Methods for additional analyses, such as subgroup analyses and adjusted analyses

Does your paper address CONSORT subitem 12b? *

Copy and paste relevant sections from the manuscript (include quotes in quotation marks "like this" to indicate direct quotes from your manuscript), or elaborate on this item by providing additional information not in the ms, or briefly explain why the item is not applicable/relevant for your study

A two-period double crossover randomized-controlled study was employed. The double-crossover study design mitigates learning bias and has been previously shown to improve statistical power [43] Patients were randomized in a 1:1 ratio to receive either the ACoE or paper-based test first, then returned 1-6 weeks later to receive the other test. Inter-test duration was limited to 1-6 weeks to control for time-, medication-, or pathology-related cognitive changes to balance for learning bias while also minimizing in disease or mental state between tests [37]. Only patients receiving the ACE-3 were randomized to enable comparison of the cognitive evaluation of the ACoE to the ACE-3 (Supplementary Figure 1). No changes were made to the study design after initiation.

X26) REB/IRB Approval and Ethical Considerations [recommended as subheading under "Methods"] (not a CONSORT item)

X26-i) Comment on ethics committee approval

	1	2	3	4	5	
subitem not at all important	○	○	○	○	○	essential

Does your paper address subitem X26-i?

Copy and paste relevant sections from the manuscript (include quotes in quotation marks "like this" to indicate direct quotes from your manuscript), or elaborate on this item by providing additional information not in the ms, or briefly explain why the item is not applicable/relevant for your study

Your answer

x26-ii) Outline informed consent procedures

Outline informed consent procedures e.g., if consent was obtained offline or online (how? Checkbox, etc.?), and what information was provided (see 4a-ii). See [6] for some items to be included in informed consent documents.

	1	2	3	4	5	
subitem not at all important	○	○	○	○	○	essential

Does your paper address subitem X26-ii?

Copy and paste relevant sections from the manuscript (include quotes in quotation marks "like this" to indicate direct quotes from your manuscript), or elaborate on this item by providing additional information not in the ms, or briefly explain why the item is not applicable/relevant for your study

Your answer

X26-iii) Safety and security procedures

Safety and security procedures, incl. privacy considerations, and any steps taken to reduce the likelihood or detection of harm (e.g., education and training, availability of a hotline)

	1	2	3	4	5	
subitem not at all important	○	○	○	○	○	essential

Does your paper address subitem X26-iii?

Copy and paste relevant sections from the manuscript (include quotes in quotation marks "like this" to indicate direct quotes from your manuscript), or elaborate on this item by providing additional information not in the ms, or briefly explain why the item is not applicable/relevant for your study

Your answer

RESULTS

13a) For each group, the numbers of participants who were randomly assigned, received intended treatment, and were analysed for the primary outcome
NPT: The number of care providers or centers performing the intervention in each group and the number of patients treated by each care provider in each center

Does your paper address CONSORT subitem 13a? *

Copy and paste relevant sections from the manuscript (include quotes in quotation marks "like this" to indicate direct quotes from your manuscript), or elaborate on this item by providing additional information not in the ms, or briefly explain why the item is not applicable/relevant for your study

132 patients were assessed for eligibility. 79 patients denied enrollment and 7 did not meet inclusion criteria. 46 patients were randomized, with 24 patients randomized to receive the ACoE first (Group 1) and 22 to receive the ACE-3 first (Group 2). 11 patients were lost to follow-up. 35 patients completed the study and were analyzed (Supplementary Figure 1). There were no adverse outcomes reported. The characteristics of each arm are available (Table 2).
We next evaluated for appropriate randomization. Cognitive scores were not significantly different between the two arms (Wilcoxon test, p = .59, Supplementary Figure 1). Nor were there significant differences in the number of patients in each arm (Chi-squared, p = .46).

13b) For each group, losses and exclusions after randomisation, together with reasons

Does your paper address CONSORT subitem 13b? (NOTE: Preferably, this is shown in a CONSORT flow diagram) *

Copy and paste relevant sections from the manuscript (include quotes in quotation marks "like this" to indicate direct quotes from your manuscript), or elaborate on this item by providing additional information not in the ms, or briefly explain why the item is not applicable/relevant for your study

132 patients were assessed for eligibility. 79 patients denied enrollment and 7 did not meet inclusion criteria. 46 patients were randomized, with 24 patients randomized to receive the ACoE first (Group 1) and 22 to receive the ACE-3 first (Group 2). 11 patients were lost to follow-up. 35 patients completed the study and were analyzed (Supplementary Figure 1). There were no adverse outcomes reported. The characteristics of each arm are available (Table 2).
We next evaluated for appropriate randomization. Cognitive scores were not significantly different between the two arms (Wilcoxon test, p = .59, Supplementary Figure 1). Nor were there significant differences in the number of patients in each arm (Chi-squared, p = .46).

13b-i) Attrition diagram

Strongly recommended: An attrition diagram (e.g., proportion of participants still logging in or using the intervention/comparator in each group plotted over time, similar to a survival curve) or other figures or tables demonstrating usage/dose/engagement.

	1	2	3	4	5	
subitem not at all important	O	O	O	O	O	essential

Does your paper address subitem 13b-i?

Copy and paste relevant sections from the manuscript or cite the figure number if applicable (include quotes in quotation marks "like this" to indicate direct quotes from your manuscript), or elaborate on this item by providing additional information not in the ms, or briefly explain why the item is not applicable/relevant for your study

Your answer

14a) Dates defining the periods of recruitment and follow-up

Does your paper address CONSORT subitem 14a? *

Copy and paste relevant sections from the manuscript (include quotes in quotation marks "like this" to indicate direct quotes from your manuscript), or elaborate on this item by providing additional information not in the ms, or briefly explain why the item is not applicable/relevant for your study

132 patients were assessed for eligibility. 79 patients denied enrollment and 7 did not meet inclusion criteria. 46 patients were randomized, with 24 patients randomized to receive the ACoE first (Group 1) and 22 to receive the ACE-3 first (Group 2). 11 patients were lost to follow-up. 35 patients completed the study and were analyzed (Supplementary Figure 1). There were no adverse outcomes reported. The characteristics of each arm are available (Table 2).
We next evaluated for appropriate randomization. Cognitive scores were not significantly different between the two arms (Wilcoxon test, p = .59, Supplementary Figure 1). Nor were there significant differences in the number of patients in each arm (Chi-squared, p = .46).

14a-i) Indicate if critical “secular events” fell into the study period

Indicate if critical “secular events” fell into the study period, e.g., significant changes in Internet resources available or “changes in computer hardware or Internet delivery resources”

	1	2	3	4	5	
subitem not at all important	○	○	○	○	○	essential

Does your paper address subitem 14a-i?

Copy and paste relevant sections from the manuscript (include quotes in quotation marks "like this" to indicate direct quotes from your manuscript), or elaborate on this item by providing additional information not in the ms, or briefly explain why the item is not applicable/relevant for your study

Your answer

14b) Why the trial ended or was stopped (early)

Does your paper address CONSORT subitem 14b? *

Copy and paste relevant sections from the manuscript (include quotes in quotation marks "like this" to indicate direct quotes from your manuscript), or elaborate on this item by providing additional information not in the ms, or briefly explain why the item is not applicable/relevant for your study

132 patients were assessed for eligibility. 79 patients denied enrollment and 7 did not meet inclusion criteria. 46 patients were randomized, with 24 patients randomized to receive the ACoE first (Group 1) and 22 to receive the ACE-3 first (Group 2). 11 patients were lost to follow-up. 35 patients completed the study and were analyzed (Supplementary Figure 1). There were no adverse outcomes reported. The characteristics of each arm are available (Table 2).

We next evaluated for appropriate randomization. Cognitive scores were not significantly different between the two arms (Wilcoxon test, p = .59, Supplementary Figure 1). Nor were there significant differences in the number of patients in each arm (Chi-squared, p = .46).

15) A table showing baseline demographic and clinical characteristics for each group

NPT: When applicable, a description of care providers (case volume, qualification, expertise, etc.) and centers (volume) in each group

Does your paper address CONSORT subitem 15? *

Copy and paste relevant sections from the manuscript (include quotes in quotation marks "like this" to indicate direct quotes from your manuscript), or elaborate on this item by providing additional information not in the ms, or briefly explain why the item is not applicable/relevant for your study

Your answer

15-i) Report demographics associated with digital divide issues

In ehealth trials it is particularly important to report demographics associated with digital divide issues, such as age, education, gender, social-economic status, computer/Internet/ehealth literacy of the participants, if known.

	1	2	3	4	5	
subitem not at all important	○	○	○	○	○	essential

Does your paper address subitem 15-i? *

Copy and paste relevant sections from the manuscript (include quotes in quotation marks "like this" to indicate direct quotes from your manuscript), or elaborate on this item by providing additional information not in the ms, or briefly explain why the item is not applicable/relevant for your study

132 patients were assessed for eligibility. 79 patients denied enrollment and 7 did not meet inclusion criteria. 46 patients were randomized, with 24 patients randomized to receive the ACoE first (Group 1) and 22 to receive the ACE-3 first (Group 2). 11 patients were lost to follow-up. 35 patients completed the study and were analyzed (Supplementary Figure 1). There were no adverse outcomes reported. The characteristics of each arm are available (Table 2).
We next evaluated for appropriate randomization. Cognitive scores were not significantly different between the two arms (Wilcoxon test, $p = .59$, Supplementary Figure 1). Nor were there significant differences in the number of patients in each arm (Chi-squared, $p = .46$).

16) For each group, number of participants (denominator) included in each analysis and whether the analysis was by original assigned groups

16-i) Report multiple “denominators” and provide definitions

Report multiple “denominators” and provide definitions: Report N’s (and effect sizes) “across a range of study participation [and use] thresholds” [1], e.g., N exposed, N consented, N used more than x times, N used more than y weeks, N participants “used” the intervention/comparator at specific pre-defined time points of interest (in absolute and relative numbers per group). Always clearly define “use” of the intervention.

	1	2	3	4	5	
subitem not at all important	○	○	○	○	○	essential

Does your paper address subitem 16-i? *

Copy and paste relevant sections from the manuscript (include quotes in quotation marks "like this" to indicate direct quotes from your manuscript), or elaborate on this item by providing additional information not in the ms, or briefly explain why the item is not applicable/relevant for your study

132 patients were assessed for eligibility. 79 patients denied enrollment and 7 did not meet inclusion criteria. 46 patients were randomized, with 24 patients randomized to receive the ACoE first (Group 1) and 22 to receive the ACE-3 first (Group 2). 11 patients were lost to follow-up. 35 patients completed the study and were analyzed (Supplementary Figure 1). There were no adverse outcomes reported. The characteristics of each arm are available (Table 2).

We next evaluated for appropriate randomization. Cognitive scores were not significantly different between the two arms (Wilcoxon test, $p = .59$, Supplementary Figure 1). Nor were there significant differences in the number of patients in each arm (Chi-squared, $p = .46$).

16-ii) Primary analysis should be intent-to-treat

Primary analysis should be intent-to-treat, secondary analyses could include comparing only “users”, with the appropriate caveats that this is no longer a randomized sample (see 18-i).

	1	2	3	4	5	
subitem not at all important	○	○	○	○	○	essential

Does your paper address subitem 16-ii?

Copy and paste relevant sections from the manuscript (include quotes in quotation marks "like this" to indicate direct quotes from your manuscript), or elaborate on this item by providing additional information not in the ms, or briefly explain why the item is not applicable/relevant for your study

Your answer

17a) For each primary and secondary outcome, results for each group, and the estimated effect size and its precision (such as 95% confidence interval)

Does your paper address CONSORT subitem 17a? *

Copy and paste relevant sections from the manuscript (include quotes in quotation marks "like this" to indicate direct quotes from your manuscript), or elaborate on this item by providing additional information not in the ms, or briefly explain why the item is not applicable/relevant for your study

132 patients were assessed for eligibility. 79 patients denied enrollment and 7 did not meet inclusion criteria. 46 patients were randomized, with 24 patients randomized to receive the ACoE first (Group 1) and 22 to receive the ACE-3 first (Group 2). 11 patients were lost to follow-up. 35 patients completed the study and were analyzed (Supplementary Figure 1). There were no adverse outcomes reported. The characteristics of each arm are available (Table 2).

We next evaluated for appropriate randomization. Cognitive scores were not significantly different between the two arms (Wilcoxon test, p = .59, Supplementary Figure 1). Nor were there significant differences in the number of patients in each arm (Chi-squared, p = .46).

17a-i) Presentation of process outcomes such as metrics of use and intensity of use

In addition to primary/secondary (clinical) outcomes, the presentation of process outcomes such as metrics of use and intensity of use (dose, exposure) and their operational definitions is critical. This does not only refer to metrics of attrition (13-b) (often a binary variable), but also to more continuous exposure metrics such as “average session length”. These must be accompanied by a technical description how a metric like a “session” is defined (e.g., timeout after idle time) [1] (report under item 6a).

	1	2	3	4	5	
subitem not at all important	○	○	○	○	○	essential

Does your paper address subitem 17a-i?

Copy and paste relevant sections from the manuscript (include quotes in quotation marks "like this" to indicate direct quotes from your manuscript), or elaborate on this item by providing additional information not in the ms, or briefly explain why the item is not applicable/relevant for your study

Your answer

17b) For binary outcomes, presentation of both absolute and relative effect sizes is recommended

Does your paper address CONSORT subitem 17b? *

Copy and paste relevant sections from the manuscript (include quotes in quotation marks "like this" to indicate direct quotes from your manuscript), or elaborate on this item by providing additional information not in the ms, or briefly explain why the item is not applicable/relevant for your study

132 patients were assessed for eligibility. 79 patients denied enrollment and 7 did not meet inclusion criteria. 46 patients were randomized, with 24 patients randomized to receive the ACoE first (Group 1) and 22 to receive the ACE-3 first (Group 2). 11 patients were lost to follow-up. 35 patients completed the study and were analyzed (Supplementary Figure 1). There were no adverse outcomes reported. The characteristics of each arm are available (Table 2).

We next evaluated for appropriate randomization. Cognitive scores were not significantly different between the two arms (Wilcoxon test, p = .59, Supplementary Figure 1). Nor were there significant differences in the number of patients in each arm (Chi-squared, p = .46).

18) Results of any other analyses performed, including subgroup analyses and adjusted analyses, distinguishing pre-specified from exploratory

Does your paper address CONSORT subitem 18? *

Copy and paste relevant sections from the manuscript (include quotes in quotation marks "like this" to indicate direct quotes from your manuscript), or elaborate on this item by providing additional information not in the ms, or briefly explain why the item is not applicable/relevant for your study

Your answer

18-i) Subgroup analysis of comparing only users

A subgroup analysis of comparing only users is not uncommon in ehealth trials, but if done, it must be stressed that this is a self-selected sample and no longer an unbiased sample from a randomized trial (see 16-iii).

	1	2	3	4	5	
subitem not at all important	○	○	○	○	○	essential

Does your paper address subitem 18-i?

Copy and paste relevant sections from the manuscript (include quotes in quotation marks "like this" to indicate direct quotes from your manuscript), or elaborate on this item by providing additional information not in the ms, or briefly explain why the item is not applicable/relevant for your study

Your answer

19) All important harms or unintended effects in each group

(for specific guidance see CONSORT for harms)

Does your paper address CONSORT subitem 19? *

Copy and paste relevant sections from the manuscript (include quotes in quotation marks "like this" to indicate direct quotes from your manuscript), or elaborate on this item by providing additional information not in the ms, or briefly explain why the item is not applicable/relevant for your study

132 patients were assessed for eligibility. 79 patients denied enrollment and 7 did not meet inclusion criteria. 46 patients were randomized, with 24 patients randomized to receive the ACoE first (Group 1) and 22 to receive the ACE-3 first (Group 2). 11 patients were lost to follow-up. 35 patients completed the study and were analyzed (Supplementary Figure 1). There were no adverse outcomes reported. The characteristics of each arm are available (Table 2).
We next evaluated for appropriate randomization. Cognitive scores were not significantly different between the two arms (Wilcoxon test, p = .59, Supplementary Figure 1). Nor were there significant differences in the number of patients in each arm (Chi-squared, p = .46).

19-i) Include privacy breaches, technical problems

Include privacy breaches, technical problems. This does not only include physical “harm” to participants, but also incidents such as perceived or real privacy breaches [1], technical problems, and other unexpected/unintended incidents. “Unintended effects” also includes unintended positive effects [2].

	1	2	3	4	5	
subitem not at all important	○	○	○	○	○	essential

Does your paper address subitem 19-i?

Copy and paste relevant sections from the manuscript (include quotes in quotation marks "like this" to indicate direct quotes from your manuscript), or elaborate on this item by providing additional information not in the ms, or briefly explain why the item is not applicable/relevant for your study

Your answer

19-ii) Include qualitative feedback from participants or observations from staff/researchers

Include qualitative feedback from participants or observations from staff/researchers, if available, on strengths and shortcomings of the application, especially if they point to unintended/unexpected effects or uses. This includes (if available) reasons for why people did or did not use the application as intended by the developers.

	1	2	3	4	5	
subitem not at all important	○	○	○	○	○	essential

Does your paper address subitem 19-ii?

Copy and paste relevant sections from the manuscript (include quotes in quotation marks "like this" to indicate direct quotes from your manuscript), or elaborate on this item by providing additional information not in the ms, or briefly explain why the item is not applicable/relevant for your study

Your answer

DISCUSSION

22) Interpretation consistent with results, balancing benefits and harms, and considering other relevant evidence

NPT: In addition, take into account the choice of the comparator, lack of or partial blinding, and unequal expertise of care providers or centers in each group

22-i) Restate study questions and summarize the answers suggested by the data, starting with primary outcomes and process outcomes (use)

Restate study questions and summarize the answers suggested by the data, starting with primary outcomes and process outcomes (use).

	1	2	3	4	5	
subitem not at all important	○	○	○	○	○	essential

Does your paper address subitem 22-i? *

Copy and paste relevant sections from the manuscript (include quotes in quotation marks "like this" to indicate direct quotes from your manuscript), or elaborate on this item by providing additional information not in the ms, or briefly explain why the item is not applicable/relevant for your study

Your answer

22-ii) Highlight unanswered new questions, suggest future research

Highlight unanswered new questions, suggest future research.

	1	2	3	4	5	
subitem not at all important	○	○	○	○	○	essential

Does your paper address subitem 22-ii?

Copy and paste relevant sections from the manuscript (include quotes in quotation marks "like this" to indicate direct quotes from your manuscript), or elaborate on this item by providing additional information not in the ms, or briefly explain why the item is not applicable/relevant for your study

Your answer

20) Trial limitations, addressing sources of potential bias, imprecision, and, if relevant, multiplicity of analyses

20-i) Typical limitations in ehealth trials

Typical limitations in ehealth trials: Participants in ehealth trials are rarely blinded. Ehealth trials often look at a multiplicity of outcomes, increasing risk for a Type I error. Discuss biases due to non-use of the intervention/usability issues, biases through informed consent procedures, unexpected events.

	1	2	3	4	5	
subitem not at all important	○	○	○	○	○	essential

Does your paper address subitem 20-i? *

Copy and paste relevant sections from the manuscript (include quotes in quotation marks "like this" to indicate direct quotes from your manuscript), or elaborate on this item by providing additional information not in the ms, or briefly explain why the item is not applicable/relevant for your study

Your answer

21) Generalisability (external validity, applicability) of the trial findings

NPT: External validity of the trial findings according to the intervention, comparators, patients, and care providers or centers involved in the trial

21-i) Generalizability to other populations

Generalizability to other populations: In particular, discuss generalizability to a general Internet population, outside of a RCT setting, and general patient population, including applicability of the study results for other organizations

	1	2	3	4	5	
subitem not at all important	◯	◯	◯	◯	◯	essential

Does your paper address subitem 21-i?

Copy and paste relevant sections from the manuscript (include quotes in quotation marks "like this" to indicate direct quotes from your manuscript), or elaborate on this item by providing additional information not in the ms, or briefly explain why the item is not applicable/relevant for your study

Your answer

21-ii) Discuss if there were elements in the RCT that would be different in a routine application setting

Discuss if there were elements in the RCT that would be different in a routine application setting (e.g., prompts/reminders, more human involvement, training sessions or other co-interventions) and what impact the omission of these elements could have on use, adoption, or outcomes if the intervention is applied outside of a RCT setting.

	1	2	3	4	5	
subitem not at all important	◯	◯	◯	◯	◯	essential

Does your paper address subitem 21-ii?

Copy and paste relevant sections from the manuscript (include quotes in quotation marks "like this" to indicate direct quotes from your manuscript), or elaborate on this item by providing additional information not in the ms, or briefly explain why the item is not applicable/relevant for your study

Your answer

OTHER INFORMATION

23) Registration number and name of trial registry

Does your paper address CONSORT subitem 23? *

Copy and paste relevant sections from the manuscript (include quotes in quotation marks "like this" to indicate direct quotes from your manuscript), or elaborate on this item by providing additional information not in the ms, or briefly explain why the item is not applicable/relevant for your study

Your answer

24) Where the full trial protocol can be accessed, if available

Does your paper address CONSORT subitem 24? *

Cite a Multimedia Appendix, other reference, or copy and paste relevant sections from the manuscript (include quotes in quotation marks "like this" to indicate direct quotes from your manuscript), or elaborate on this item by providing additional information not in the ms, or briefly explain why the item is not applicable/relevant for your study

A two-period double crossover randomized-controlled study was employed. The double-crossover study design mitigates learning bias and has been previously shown to improve statistical power [43] Patients were randomized in a 1:1 ratio to receive either the ACoE or paper-based test first, then returned 1-6 weeks later to receive the other test. Inter-test duration was limited to 1-6 weeks to control for time-, medication-, or pathology-related cognitive changes to balance for learning bias while also minimizing in disease or mental state between tests [37]. Only patients receiving the ACE-3 were randomized to enable comparison of the cognitive evaluation of the ACoE to the ACE-3 (Supplementary Figure 1). No changes were made to the study design after initiation.
Patients with and without cognitive complaints were recruited. Inclusion criterion was English fluency and being over age 18. English fluency was evaluated by the attending clinician. Exclusion criteria were acute medical conditions contributing to cognitive state, acute psychiatric disorders contributing to cognitive state, delirious states, or disabilities restricting ability to utilize screens, disabilities restricting ability to receive visual and auditory instructions, or developmental delay.

25) Sources of funding and other support (such as supply of drugs), role of funders

Does your paper address CONSORT subitem 25? *

Copy and paste relevant sections from the manuscript (include quotes in quotation marks "like this" to indicate direct quotes from your manuscript), or elaborate on this item by providing additional information not in the ms, or briefly explain why the item is not applicable/relevant for your study

A two-period double crossover randomized-controlled study was employed. The double-crossover study design mitigates learning bias and has been previously shown to improve statistical power [43] Patients were randomized in a 1:1 ratio to receive either the ACoE or paper-based test first, then returned 1-6 weeks later to receive the other test. Inter-test duration was limited to 1-6 weeks to control for time-, medication-, or pathology-related cognitive changes to balance for learning bias while also minimizing in disease or mental state between tests [37]. Only patients receiving the ACE-3 were randomized to enable comparison of the cognitive evaluation of the ACoE to the ACE-3 (Supplementary Figure 1). No changes were made to the study design after initiation.
Patients with and without cognitive complaints were recruited. Inclusion criterion was English fluency and being over age 18. English fluency was evaluated by the attending clinician. Exclusion criteria were acute medical conditions contributing to cognitive state, acute psychiatric disorders contributing to cognitive state, delirious states, or disabilities restricting ability to utilize screens, disabilities restricting ability to receive visual and auditory instructions, or developmental delay.

X27) Conflicts of Interest (not a CONSORT item)

X27-i) State the relation of the study team towards the system being evaluated

In addition to the usual declaration of interests (financial or otherwise), also state the relation of the study team towards the system being evaluated, i.e., state if the authors/evaluators are distinct from or identical with the developers/sponsors of the intervention.

	1	2	3	4	5	
subitem not at all important	○	○	○	○	○	essential

Does your paper address subitem X27-i?

Copy and paste relevant sections from the manuscript (include quotes in quotation marks "like this" to indicate direct quotes from your manuscript), or elaborate on this item by providing additional information not in the ms, or briefly explain why the item is not applicable/relevant for your study

Your answer

About the CONSORT EHEALTH checklist

As a result of using this checklist, did you make changes in your manuscript? *

- yes, major changes
- yes, minor changes
- no

What were the most important changes you made as a result of using this checklist?

Your answer

How much time did you spend on going through the checklist INCLUDING making changes in your manuscript *

Your answer

As a result of using this checklist, do you think your manuscript has improved? *

- ◯ yes
- ◉ no
- ◯ Other:

Would you like to become involved in the CONSORT EHEALTH group?

This would involve for example becoming involved in participating in a workshop and writing an "Explanation and Elaboration" document

- ◯ yes
- ◯ no
- ◯ Other:

Any other comments or questions on CONSORT EHEALTH

Your answer

STOP - Save this form as PDF before you click submit

To generate a record that you filled in this form, we recommend to generate a PDF of this page (on a Mac, simply select "print" and then select "print as PDF") before you submit it.

When you submit your (revised) paper to JMIR, please upload the PDF as supplementary file.

Don't worry if some text in the textboxes is cut off, as we still have the complete information in our database. Thank you!

Final step: Click submit !

Click submit so we have your answers in our database!

Submit Clear form

Never submit passwords through Google Forms.

This content is neither created nor endorsed by Google. - Contact form owner - Terms of Service - Privacy Policy

Does this form look suspicious? Report

Google Forms
